# Deoxygenative Coupling of CO with a Tetrametallic Magnesium Hydride Complex

**DOI:** 10.1002/anie.202319626

**Published:** 2024-02-29

**Authors:** Wenbang Yang, Andrew J. P. White, Mark R. Crimmin

**Affiliations:** ^1^ Department of Chemistry Imperial College London Molecular Sciences Research Hub Imperial College London 82 Wood Lane, White City London W12 0BZ UK

**Keywords:** Carbon Monoxide, Deoxygenation, Homologation, Magnesium, Hydride

## Abstract

Addition of CO to a tetrametallic magnesium hydride cluster results in both carbon‐carbon bond formation and deoxygenation to generate an acetaldehyde enolate [C_2_OH_3_]^−^ which remains coordinated to the cluster. To the best of our knowledge, this is the first example of formation of an isolable complex containing an [C_2_OH_3_]^−^ fragment from reaction of CO with a metal hydride, and the first example of CO homologation and deoxygenation at a main group metal. DFT studies suggest that key steps in the mechanism involve nucleophilic attack of an oxymethylene on a formyl ligand to generate an unstable [C_2_O_2_H_3_]^3−^ fragment, which undergoes subsequent deoxygenation.

## Graphical abstract

Addition of CO to a tetrametallic magnesium hydride cluster results in both carbon‐carbon bond formation and deoxygenation to generate an acetaldehyde enolate [C_2_OH_3_]^−^ which remains coordinated to the cluster. DFT studies suggest that key steps in the mechanism involve nucleophilic attack of an oxymethylene on a formyl ligand to generate an unstable [C_2_O_2_H_3_]^3−^ fragment, which undergoes subsequent deoxygenation.

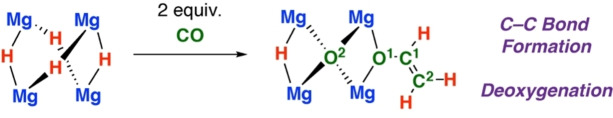

Reactions of carbon monoxide (CO) with homogeneous metal hydride complexes are of fundamental interest and direct relevance to the Fischer–Tropsch (F–T) process.[^1^, ^2^] In recent years, there has been growing interest in the use of multimetallic compounds in these reactions as they potentially model metal surfaces which contain multiple metal and hydride sites in proximity. These systems may also expose new cooperative mechanisms and modes of reactivity often not seen in their monometallic counterparts.^[3]^

Multimetallic systems are persistent across the very few transition metal systems that are capable of coupling *and* deoxygenating two or more CO units. Deoxygenation of CO is particularly challenging and is not well understood in homogeneous models of the F–T process. The CO bond has a homolytic bond dissociation energy of 257 kcal mol^−1^ and is one of the strongest chemical units known. Hou and co‐workers have reported that tetrametallic yttrium hydride complexes supported by cyclopentadienyl ligands react with CO to form CH_2_=CH_2_.^[4]^ Similarly, trimetallic yttrium complexes supported by tris(pyrazolyl)borate ligands react with CO to form a new propenolate ligand.^[5]^ Related reactions are known for group 4 complexes, with trimetallic titanium hydride clusters effecting the hydrodeoxygenative cyclotetramerization of CO to yield cyclobuten‐3,4‐diyl‐1,2‐diolate motifs.^[6]^ Common to these systems are the formation of strong, bridging M−O−M groups in the reaction products that likely provide a thermodynamic driving force for the deoxygenation step (Figure 1). While, to the best of our knowledge there are no examples of coupling and deoxygenation of CO with main group metals,^[7]^ the semi‐metals silicon and boron have both been used to such end. Namely, bis(silylene) complexes incorporating two low‐valent silicon centres have been shown to react with CO to form deoxygenated ketene motifs.[^8^, ^9^] Disilenide anions also react with CO to effect its coupling and deoxygenation.^[10]^ The conversion of CO into short chain linear alcohols and aldehydes can also be promoted by either dialkyl boranes or a combination of a trialkylborane and a suitable hydride source.[^11^, ^12^, ^13^]

**Figure 1:**
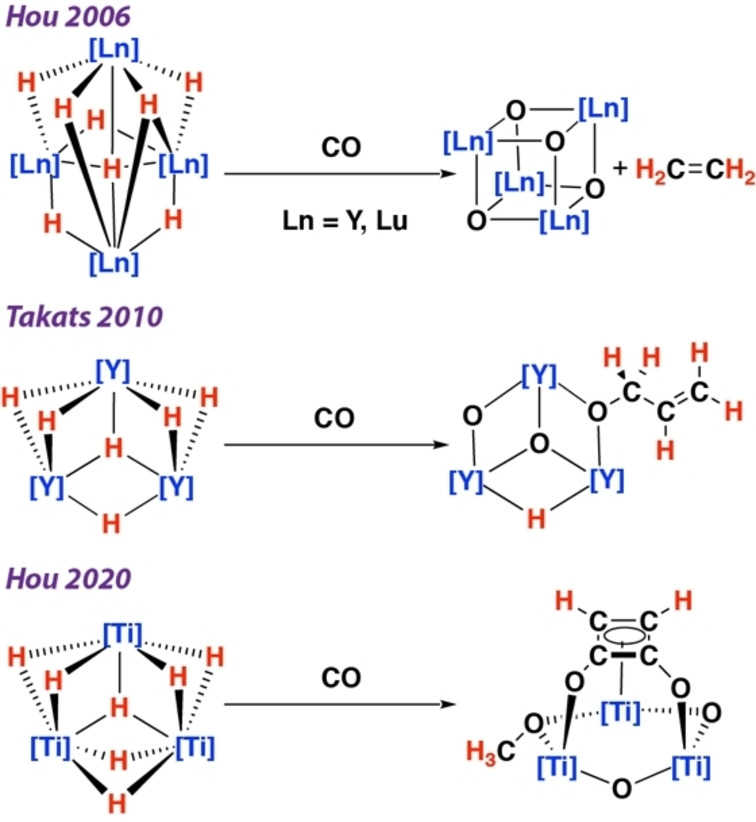
Examples of CO coupling and deoxygenation with metal hydrides. Non‐hydride and CO derived ligands have been omitted for clarity.

In this paper, we show that a tetrametallic magnesium hydride cluster is capable of coupling and deoxygenating CO to form an acetaldehyde fragment that remains bound to the cluster as an enolate. Using DFT calculations, we propose a mechanism in which the tetrameric Mg_4_ entity at the core of the cluster plays a key role in bond making and bond breaking. The discovery complements known examples of CO coupling with dimeric magnesium hydride reagents, which to date have provided no evidence for deoxygenation.[^14^, ^15^, ^16^]

The tetrametallic magnesium hydride cluster **1** was originally reported by Harder and co‐workers, related species have recently been reported by Kretschmer and co‐workers.[^17^, ^18^, ^19^] Reaction of **1** with CO (1 atm. 60 °C) led to selective formation of **2** (Figure 2a). **2** incorporates a new C_2_ fragment derived from the coupling and deoxygenation of CO. The new C_2_ fragment is best formulated as an enolate of acetaldehyde, formed from a 1 : 2 reaction stoichiometry and combination of three hydride equivalents with two molecules of CO. **2** is thermally stable up to 120 °C, and shows no sign of further deoxygenation to generate ethene.

**Figure 2:**
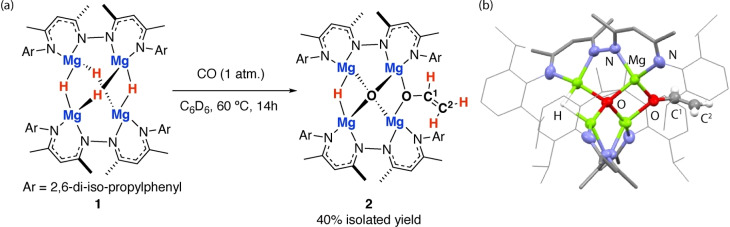
(a) Tetrameric magnesium hydride cluster **1** and its reaction with CO. (b) The crystal structure of **2**.

The reaction is under kinetic control and a single hydride site remains unreacted in **2**. **2** was characterized by a series of diagnostic resonances in the ^1^H and ^13^C NMR spectra. The three protons of the acetaldehyde enolate appear as distinct resonances at δ_H_=2.86 (d, *J*=13.6 Hz), 3.01 (d, *J*=6.0 Hz) and 4.83 (dd, *J*=13.6, 6.0 Hz) ppm with a coupling pattern diagnostic of an ABX spin‐system. HSQC and HMBC correlate these environments to ^13^C NMR resonances at δ_C_=93.1 and 148.8 ppm. The unreacted hydride of **2** is also clear from the ^1^H NMR spectrum appearing as a singlet at δ_H_=2.86 ppm. A similar C_2_ enolate has been suggested as an intermediate in the formation of CH_2_=CH_2_ from reaction Ln_4_H_8_ clusters with CO; the complex could not be isolated but was tentatively assigned at low temperature.^[4]^ The origin of CO as the carbon source for **2** was unambiguously confirmed by isotope labelling experiments. Repeating reactions of **1** with ^13^CO, allowed the isolation of ^**13**^ **C_2_‐2**, spectroscopic analysis of which show the two expected carbon‐environments in the ^13^C NMR, now with a mutual ^1^ *J*_C−C_ coupling of 78.3 Hz. ^1^ *J*_C−H_ ranging between 158.2–174.4 Hz are also apparent in the ^1^H NMR spectrum of ^**13**^ **C_2_‐2**.

In the solid state, **2** crystallises in a tetragonal space group (Figure 2b). The core of **2** is made up of a distorted tetrahedra of magnesium sites that bridge a central μ_4_ oxide ligand, formally derived from oxygen atom abstraction from CO. Each β‐diketiminate ligand is dinucleating and coordinates two adjacent Mg atoms along opposite edges of the tetrahedra. The rigid structure of the ligand is perfectly suited to align with the Mg−Mg separation of 3.3 Å in **2**. Of the remaining edges of the Mg_4_ tetrahedra one is bridged by a hydride ligand, another the C_2_ ligand from coupling and deoxygenation of CO, the rest remain open. The C^1^−C^2^ and C^1^−O^1^ bond lengths take values of 1.29(3) and 1.353(17) Å respectively and are consistent with the proposed formulation as an acetaldehyde enolate. The hydride and enolate sites are symmetry related, and there is positional disorder between these positions. Nevertheless, the structure is consistent with the solution characterization of **2**.

Kinetics on the reaction of **1** with CO were conducted across the 50–80 °C temperature range at 10 °C intervals. This analysis is complicated by the variable solubility of CO with temperature and modest conversion of **1** to **2**. Data can be fitted to 1^st^ order consumption of **1**. Eyring analysis gives ΔH^≠^=+7.4 kcal mol^−1^, ΔS^≠^=−55 cal mol^−1^ K^−1^ and ΔG^≠^ (298 K)=23.7 kcal mol^−1^.

Despite the observation that **2** is inert toward further reaction with CO, addition of alternative electrophiles demonstrate that the remaining hydride of this complex is still nucleophilic and is the preferential site for onwards reaction. Addition of **2** to benzophenone, benzonitrile, 2,6‐dimethylphenyl isocyanide, and tert‐butyl isocyanate led to the formation of **3 a**, **3 b**, **3 c** and **3 d** respectively (Figure 3). The acetaldehyde enolate remains intact throughout these reactions and no further C−C bond formation e.g. to form C_3_ chains is observed. **3 a** was characterised by a diagnostic resonance at δ_H_=5.39 ppm corresponding to the new methine environment of the carbinol anion, while **3 b** and **3 c** demonstrate a downfield resonance at δ_H_=8.17 ppm and δ_H_=7.98 ppm respectively assigned to the new metalated imine fragment. Single crystals of both **3 a** and **3 b** could be obtained. Data for **3 a** could not be suitably modelled due to extreme disorder. **3 b** crystallises in an orthorhombic point group, the structure is reminiscent of **2** but with the newly formed metalated imine bridging two magnesium sites in a κ^2^‐C,N binding mode (Figure 4). The acetaldehyde motif is again positionally disordered, in the major component, the C^1^−C^2^ and C^1^−O^1^ bond lengths take values of 1.295(14) and 1.350(9) Å respectively and are consistent with those found for **2**. Single crystals of **3 b** and **3 d** were not obtained however, the multinuclear NMR data are consistent with the proposed formulation. Addition of H_2_, PhSiH_3_, and HBpin to **2** gave no evidence for liberation of C_2_ fragment, through sigma‐bond metathesis reactions.

**Figure 3:**
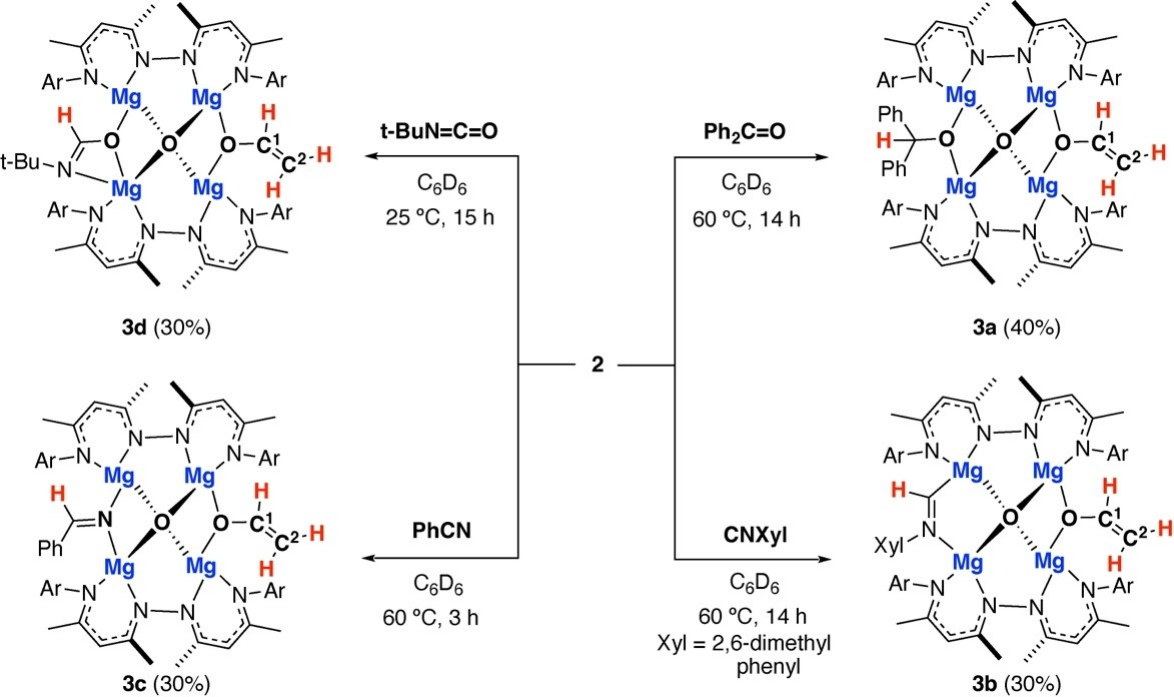
Reactions of **2** with Ph_2_CO, CNXyl (Xyl=2,6‐dimethylphenyl), PhCN, and t‐BuN=C=O. Ar=2,6‐di‐iso‐propylphenyl. Isolated yields in parentheses.

**Figure 4:**
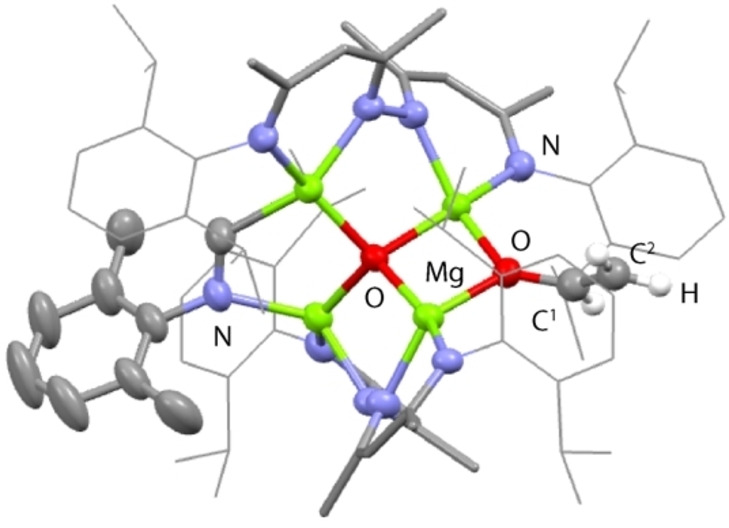
The crystal structure of **3b**.

To better understand the potential mechanism of formation for **2** a series of DFT calculations were conducted (Figure 5). A feasible pathway was calculated for formation of **2** from **1**+2CO. The calculated mechanism can be broadly separated into two phases. The first phase is initiated by 1,1‐insertion of CO into a Mg−H bond of **1** and forms **Int‐2** via an accessible transition state **TS‐1** (ΔG^≠^_298K_=23.5 kcal mol^−1^). **Int‐2** is an unstable intermediate and rotation of the newly formed formyl ligand by **TS‐2** (ΔG^≠^_298K_=25.5 kcal mol^−1^) leads to the thermodynamically more stable isomer **Int‐3** (ΔG^°^_298K_=+3.5 kcal mol^−1^) in which the ligand now bridges two magnesium sites in a κ^2^‐C,O coordination mode.

**Figure 5:**
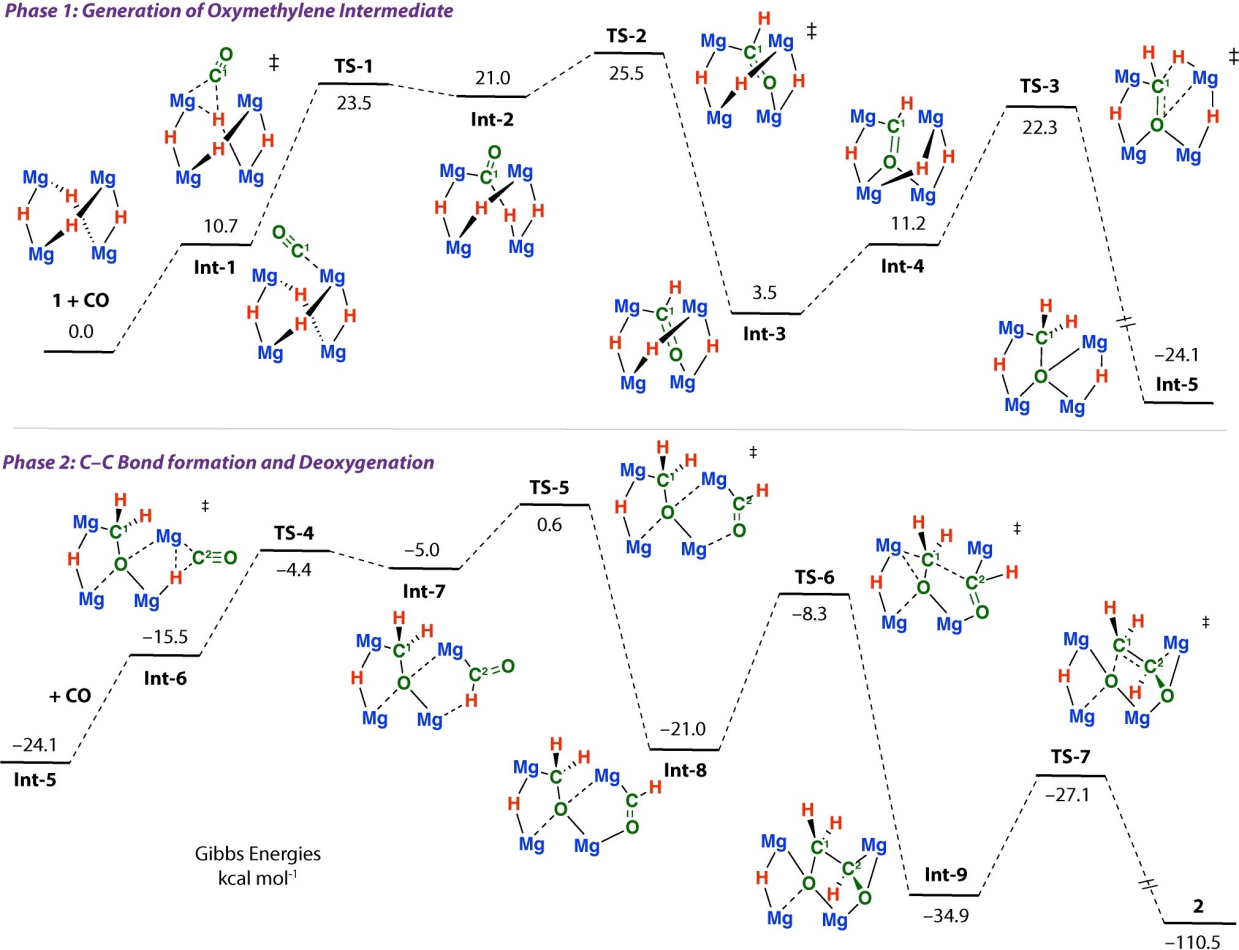
Proposed pathway for CO coupling and deoxygenation based on DFT calculations G09: ωB97X‐D/6‐311+G**/PCM (benzene)//ωB97X‐D/6‐31G**/6‐311+G*/SDDAll (Mg).

From **Int‐3**, reorganisation to **Int‐4**, followed by a second hydride transfer to the formyl ligand by **TS‐3** leads to the oxymethylene intermediate **Int‐5** (ΔG^≠^_298K_=22.3 kcal mol^−1^). **TS‐2** has the highest overall barrier and is expected to be rate‐limiting. In line with this prediction, monitoring reactions of **1** with CO as a function of time at 25 °C, provided no clear evidence for either formyl or oxymethylene complexes as reaction intermediates, suggesting that if formed they are generated only transiently. Despite the assumptions and limitations of the kinetics, the experimentally determined ΔG^≠^ (298 K) of 23.7 kcal mol^−1^ is in reasonable agreement with the barrier of predicted by the DFT model of 25.5 kcal mol^−1^.

The second phase of the calculated mechanism is initiated by association of CO with **Int‐5** to form **Int‐6**, followed by 1,1‐insertion into a remaining hydride site through **TS‐4** to generate another formyl ligand in **Int‐7**. Rotation from **Int‐7** through **TS‐5** again establishes a κ^2^‐C,O coordination mode in **Int‐8**.

Alternative pathways to generate **Int‐8** based on (i) initiating the reaction from the alternative hydride site of **1** and (ii) changing the order of the steps, were considered but found to be higher in energy than that presented in Figure 4.

**Int‐8** contains both oxymethylene and formyl ligands in proximity and is perfectly setup for carbon‐carbon bond formation. Intramolecular nucleophilic attack of the oxymethylene unit on the new formyl group occurs by **TS‐6** (local barrier, ΔG^≠^_298K_=+12.7 kcal mol^−1^) leading to **Int‐9**. **TS‐6** is templated by the cluster and during this transition state the oxygen atom of the nucleophilic oxymethylene ligand is coordinated to three magnesium sites, while the electrophilic formyl group is coordinated to the fourth.

For comparison, dimeric analogues of **1** typically form ethenediolate products by nucleophilic attack of a proposed oxymethylene intermediate on CO.[^14^, ^15^, ^16^] While this step is closely related to that described by **TS‐6**, the availability of an alternate, intramolecular electrophile (e.g. formyl group vs CO) leads to a new mode of reactivity. **Int‐9** contains an unusual trianionic [C_2_H_3_O_2_]^3−^ group, primed for fragmentation. Carbon‐oxygen bond breaking occurs from **Int‐9** through **TS‐7** (local barrier, ΔG^≠^_298K_=+7.8 kcal mol^−1^) and leads to the experimentally observed product **2**. This step occurs with transfer of electron density from the breaking C^1^−Mg bond to the forming C^1^=C^2^ π‐bond, and from C^2^−O^2^ bond to the O^2^ atom. NBO calculations are consistent with the flow of electron density. For example, along the potential energy surface from **Int‐9**→**TS‐7**→**2** the Wiberg Bond Indices (WBIs) for the C^1^−Mg bond (0.12→0.03→0.00) and C^2^−O^2^ bond (0.87→0.56→0.00) decrease, while those for the C^1^−C^2^‐bond increase (1.06→1.39→1.88). NPA charges show that the charge on C^1^ (−0.59→−0.49→0.16) dissipates as the reaction progresses and the C^1^−Mg bond is broken. At the same time, charge localises on O^2^ (−1.28→−1.47→−1.91) as electrons are moved toward the central oxygen atom of the cluster. An arrow‐pushing mechanism for the key deoxygenation step is presented in Figure 6. **TS‐7** occurs with minimal structural reorganisation, however the oxygen atom that is excised from the [C_2_H_3_O_2_]^3−^ fragment, i.e. the O^2^ atom, ends up bridging all four magnesium sites and converts from a μ_3_ to μ_4_ coordination mode as the step progresses.

**Figure 6:**
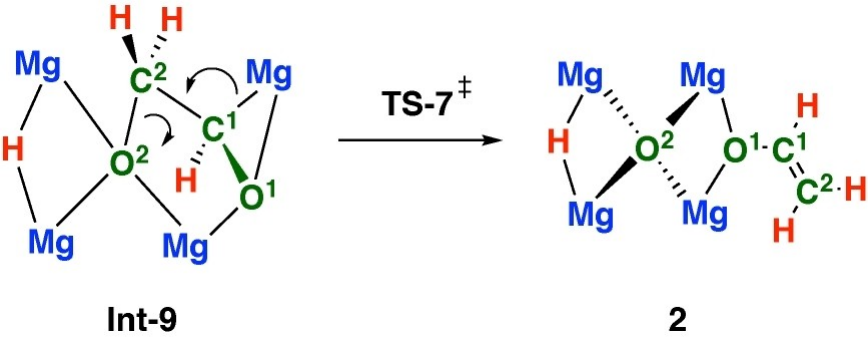
Electron‐flow in the deoxygenation step.

In summary, the calculations support the idea that **1** is pre‐organised to react with and deoxygenate CO. The controlled delivery of multiple hydrides (>2) to CO allows generation of both oxymethylene (−CH_2_O−) and formyl (−CHO) intermediates in the same molecule. The key carbon‐carbon bond forming step occurs by intramolecular nucleophilic attack of an oxymethylene on a formyl ligand generating a [C_2_H_3_O_2_]^3−^ fragment. This new pathway bypasses established reactivity on lower nuclearity (e.g. dimeric) s‐block hydride reagents with CO,[^14^, ^15^, ^16^, ^20^, ^21^, ^22^] which typically evolve from nucleophilic attack of an oxymethylene ligand on CO (rather than a formyl group) and lead to formation of ethenediolate or propanetriolate motifs. The cluster also plays an active role in the deoxygenation step; the Mg_4_ tetrahedra can coordinate to and polarise the newly [C_2_H_3_O_2_]^3−^ fragment, leading to a unique deoxygenation step. Deoxygenation involves carbon‐oxygen bond cleavage through flow of electrons from the periphery toward the centre of the cluster. The flexible coordination modes of the Mg_4_ unit and its ability to transition between μ^3^ and μ^4^ coordination environments likely facilitate this step.

## Supporting Information

Synthetic procedures, NMR spectra of all compounds, crystal structures of **2** and **3b**, crystallographic data, and computational methods (PDF). Cartesian coordinates of the DFT‐optimized structures (XYZ).

CCDC 2281296 (**2**) and 2312626 (**3b**) contain the supplementary crystallographic data for this paper. These data can be obtained free of charge via www.ccdc.cam.ac.uk/data_request/cif.

## Conflict of interests

The authors declare no conflict of interest.

## Supporting information

As a service to our authors and readers, this journal provides supporting information supplied by the authors. Such materials are peer reviewed and may be re‐organized for online delivery, but are not copy‐edited or typeset. Technical support issues arising from supporting information (other than missing files) should be addressed to the authors.

Supporting Information

## Data Availability

The data that support the findings of this study are available in the supplementary material of this article.
