# Supplementary material for: Deoxygenative Coupling of CO with a Tetrametallic Magnesium Hydride Complex
Source: Angew Chem Int Ed Engl. 2024 Feb 29;63(14):e202319626. doi: 10.1002/anie.202319626 (PMC11497278; doi:10.1002/anie.202319626)
Supplement: Supplementary file 1 — Supporting Information [file ANIE-63-e202319626-s001.pdf]

Angewandte Chemie
Eine Zeitschrift der Gesellschaft Deutscher Chemiker

Supporting Information

Deoxygenative Coupling of CO with a Tetrametallic Magnesium Hydride Complex

*W. Yang, A. J. P. White, M. R. Crimmin**

Supporting Information

Deoxygenative Coupling of CO with a Tetrametallic Magnesium Hydride Complex

Wenbang Yang, Andrew White and Mark R. Crimmin*

m.crimmin@imperial.ac.uk

Molecular Sciences Research Hub, Imperial College London, 82 Wood Lane, Shepherds Bush, W12 0BZ, UK.

Table of Contents

1) General Experimental **S3**

2) Experimental Methods **S4**

2.1) Preparation of Compounds S4

2.2) Preparation of ^{13}C Labelled Samples S15

3) Single Crystal X-ray Diffraction Data **S17**

4) DFT Studies **S21**

4.1) Computational methods S21

4.2) Comparison of Calculated and Solid-State Data S22

4.3) Calculated Reaction Pathway S25

4.4) Optimised structures S26

4.5) NBO Analysis of Some Reaction Intermediates and TS S32

4.6) QTAIM Analysis of TS7 .... S37

4.7) Other Possible Mechanism Pathway S38

5) Kinetic Analysis **S40**

5.1) Kinetics Experiments S37

6) NMR Spectra **S44**

7) IR Spectra **S49**

7) XYZ Coordinates **S50**

8) References **S90**

1) General experimental

Standard schlenk line and glovebox techniques were used for all manipulations under an inert atmosphere of dinitrogen or argon unless otherwise stated. NMR scale reactions were performed in J. Young NMR tubes. A MBraun Labmaster glovebox was employed, operating at <0.1 ppm O2 and <0.1 ppm H_2O.

Instruments: ^{1}H, ^{13}C NMR spectra were recorded on BRUKER 400 MHz or 500 MHz machines, and referenced against $SiMe_4$ (^{1}H, ^{13}C)). All peaks are referenced against residual solvent and values are quoted in ppm. Data were processed using the MestReNova software. Where needed, chemical shifts were assigned with the assistance of 2D NMR (COSY, HSQC, HMBC, DEPTQ) spectra. The coupling constants (J) are reported in hertz (Hz). The following abbreviations are used to define multiplicities: s (singlet), d (doublet), t (triplet), q (quadruplet), hept. (heptet), dd (doublet of doublets), ddd (doublet of doublets of doublets), dt (doublet of triplets), td (triplet of doublets), m (multiplet).

Single crystal X-ray data was obtained on Agilent Diffraction Xcalibur PX Ultra A and Xcalibur 3 E diffractometers, and the structures were refined using the SHELXTL, SHELX-97, and SHELX-2013 program systems.

Elemental analyses were performed by Elemental Labs (https://www.elementallab.co.uk/). Due to the small quantities of materials involved and their sensitive nature, elemental analysis was not attempted on compounds **3a-d**.

Chemicals: Solvents were dried over activated alumina from a solvent purification system (SPS) based upon the Grubbs design and de-gassed before use. Glassware was dried for >6 h prior to use at 120 °C. Benzene-d_6, fluorobenzene and trifluoro-toluene were de-gassed and stored over 3 Å molecular sieves before use. All reagents were acquired from Sigma Aldrich (Merck), Fluorochem, or VWR and used without further purification unless specified. CO was purchased from BOC Ltd and used as received. $[NN\text{-}(MgH)_2]_2$ (**1**, NN = $\{NC(Me)CH(Me)CN\text{-}2,6\text{-}i\text{-}Pr_2C_6H_3\}_2$[S1] was prepared by a literature procedure.

2) Experimental Methods

2.1 Preparation of Compounds

Preparation of 2

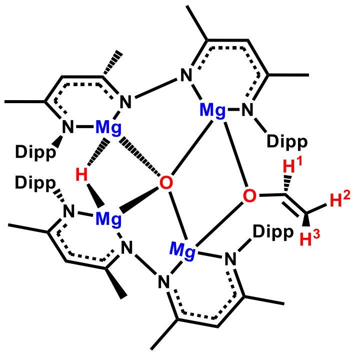

In a glovebox, **1** (20 mg, 0.018 mmol, 1 equiv) was dissolved in C_6D_6 (0.6 mL) and transferred to a J. Young NMR tube. The headspace of the NMR tube was evacuated, CO gas (~1.5 bar, about 0.135 mmol, 3.78 mg) was introduced into the NMR tube and the reaction mixture was heated at 60 °C for 14 hours. A ^{1}H NMR spectrum was taken at this time point and showed the full conversion of **1** to **2**. The J. Young NMR tube was returned to the glovebox, the solvent was removed under vacuum and the crude residue dissolved in a 1 mL Et_2O/n-pentane (1:1 v: v) mixture. The solution was filtered into a 4 mL vial and then placed in the glovebox freezer (–35 °C) for 2 days. Bright yellow crystals (**2**) were successfully obtained. The filtrated crystals were washed with cold n-pentane (3 x 1mL) and then dried in vacuo. **Yield: 17 mg,** 0.014 mmol, 40%.

^{1}H NMR (C_6D_6, 298 K, 400 MHz) δ: 7.13 – 7.00 (overlapping signals, 12H, Ar***H***), 4.83 (dd, $^3J_{H-H}$ = 13.7, 5.7 Hz, 1H, OC***H¹***=CH²H³), 4.63 (s, 2H, C***H***{C(CH₃)}₂), 4.57 (s, 2H, C***H***{C(CH₃)}₂), 3.21 (hept, $^3J_{H-H}$ = 7.0 Hz, 2H, C***H***(CH₃)₂), 3.18 (hept, $^3J_{H-H}$ = 7.0 Hz, 2H, C***H***(CH₃)₂), 3.08 (hept, $^3J_{H-H}$ = 6.9 Hz, 2H, C***H***(CH₃)₂), 3.01 (d, $^3J_{H-H}$ = 5.6 Hz, 1H, OCH¹=C***H²***H³), 2.95 (hept, $^3J_{H-H}$ = 6.9 Hz, 2H, C***H***(CH₃)₂), 2.86 (d, $^3J_{H-H}$ = 13.4 Hz, 1H, OCH¹=CH²***H³***), 2.86 (s, 1H, Mg-***H***-Mg), 1.79 (s, 6H, NC(C***H₃***)), 1.70 (s, 6H, NC(C***H₃***)), 1.64 (s, 6H, NC(C***H₃***)), 1.63 (s, 6H, NC(C***H₃***)), 1.46 (d, $^3J_{H-H}$ = 7.0 Hz, 6H, CH(C***H₃***)(CH₃)), 1.34 (d, $^3J_{H-H}$ = 6.8 Hz, 6H, CH(C***H₃***)(CH₃)), 1.32 (d, $^3J_{H-H}$ = 6.8 Hz, 6H, CH(C***H₃***)(CH₃)), 1.26 (d, $^3J_{H-H}$ = 6.9 Hz, 6H, CH(C***H₃***)(CH₃)), 1.17 (d, $^3J_{H-H}$ = 6.9 Hz, 12H, CH(C***H₃***)(CH₃)), 1.15 (d, $^3J_{H-H}$ = 7.0 Hz, 6H, CH(C***H₃***)(CH₃)), 0.73 (d, $^3J_{H-H}$ = 6.9 Hz, 6H, CH(C***H₃***)(CH₃)).

^{13}C NMR (C_6D_6, 298 K, 101 MHz) δ: 167.4 (2x N***C***(CH₃)), 167.2 (2x N***C***(CH₃)), 166.7 (2x N***C***(CH₃)), 166.2 (2x N***C***(CH₃)), 148.9 (O***C***H¹=CH²H³), 147.1 (Ar-***C***), 145.7 (Ar-***C***), 142.6 (Ar-***C***), 142.6 (Ar-***C***), 141.5 (Ar-***C***), 140.8 (Ar-***C***), 125.0 (Ar-***C***H), 124.8 (Ar-***C***H), 124.5 (Ar-***C***H), 123.9 (Ar-***C***H), 123.1

(Ar-***C***H), 122.5 (Ar-***C***H), 93.5 (OCH[1]=***C***H[2]H[3]), 93.3 (2x ***C***H{C(CH$_3$)}$_2$), 92.0 (2x **C**H{C(CH$_3$)}$_2$), 29.5 (2x ***C***H(CH$_3$)$_2$), 28.5 (2x ***C***H(CH$_3$)$_2$), 28.4 (2x ***C***H(CH$_3$)$_2$), 28.1 (2x ***C***H(CH$_3$)$_2$), 25.9 (4x CH(***C***H$_3$)(CH$_3$)), 24.4 (2x CH(***C***H$_3$)(CH$_3$)), 24.2 (2x CH(***C***H$_3$)(CH$_3$)), 24.0 (2x NC(***C***H$_3$)), 23.9 (2x NC(***C***H$_3$)), 23.8 (2x CH(***C***H$_3$)(CH$_3$)), 23.5 (2x CH(***C***H$_3$)(CH$_3$)), 23.2 (2x CH(***C***H$_3$)(CH$_3$)), 23.0 (2x CH(***C***H$_3$)(CH$_3$)), 20.1 (2x NC(***C***H$_3$)), 19.3 (2x NC(***C***H$_3$)). Some Ar***C*** resonances are overlapping and cannot be observed.

IR (ATR, cm^{-1}): 2956(s), 2922(m), 2866(m), 1625 (m), 1540(s), 1510(s), 1456(w), 1434(m), 1413(w), 1372(s), 1358(s), 1340(m), 1312(s), 1284(m), 1252(s).

Anal. Calc. ($C_{74}H_{110}Mg_4N_8O_3$): C, 70.71; H, 8.82; N, 8.91. Found: C, 70.63; H, 8.44; N, 8.67. The CHN results match the expected values for **2-Et$_2$O**.

Preparation of 2-$^{13}C_2$

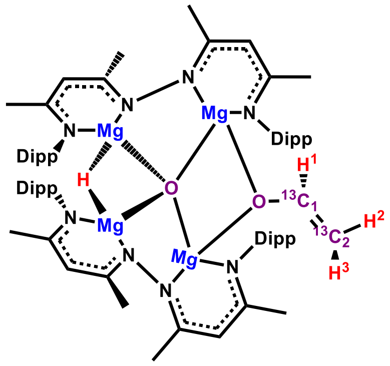

In a glovebox, **1** (20 mg, 0.018 mmol, 1 equiv) was dissolved in C_6D_6 (0.6 mL) and transferred to a J. Young NMR tube. The headspace of the NMR tube was evacuated, ^{13}CO gas (~1.5 bar, about 0.135 mmol, 3.78 mg) was introduced into the NMR tube and the reaction mixture was heated at 60 °C for 14 hours. ^1H NMR, ^{13}C NMR and some 2D (COSY, HSQC, HMBC, DEPTQ) spectra were taken at this time point and showed the full conversion of **1** to **2-$^{13}C_2$**.

^1H NMR (C_6D_6, 298 K, 400 MHz) δ: 7.13 – 7.00 (overlapping signals, 12H, Ar***H***), 4.81 (dddd, *J* = 174.4, 13.2, 13.2, 5.8 Hz, 1H, $O^{13}C_1\boldsymbol{H^1}{=}^{13}C_2H^2H^3$), 4.63 (s, 2H, C***H***{C(CH$_3$)}$_2$), 4.57 (s, 2H, C***H***{C(CH$_3$)}$_2$), 3.21 (hept, $^3J_{H\text{-}H}$ = 7.0 Hz, 2H, C***H***(CH$_3$)$_2$), 3.18 (hept, $^3J_{H\text{-}H}$ = 7.0 Hz, 2H, C***H***(CH$_3$)$_2$), 3.08 (hept, $^3J_{H\text{-}H}$ = 6.9 Hz, 2H, C***H***(CH$_3$)$_2$), 3.00 (ddd, *J* = 160.8, 6.0, 5.8 Hz, 1H, $O^{13}C_1H^1{=}^{13}C_2\boldsymbol{H^2}H^3$), 2.95 (hept, $^3J_{H\text{-}H}$ = 6.9 Hz, 2H, C***H***(CH$_3$)$_2$), 2.86 (s, 1H, Mg-***H***-Mg), 2.87 (ddd, *J* = 153.2, 13.2, 6.0 Hz 1H, $O^{13}C_1H^1{=}^{13}C_2H^2\boldsymbol{H^3}$), 1.79 (s, 6H, NC(C***H$_3$***)), 1.70 (s, 6H, NC(C***H$_3$***)), 1.64 (s, 6H, NC(C***H$_3$***)), 1.63 (s, 6H, NC(C***H$_3$***)), 1.46 (d, $^3J_{H\text{-}H}$ = 7.0 Hz, 6H, CH(C***H$_3$***)(CH$_3$)), 1.34 (d, $^3J_{H\text{-}H}$ = 3.3 Hz, 6H, CH(C***H$_3$***)(CH$_3$)), 1.32 (d, $^3J_{H\text{-}H}$ = 3.2 Hz, 6H, CH(C***H$_3$***)(CH$_3$)), 1.26 (d, $^3J_{H\text{-}H}$ = 6.9 Hz, 6H, CH(C***H$_3$***)(CH$_3$)), 1.17 (d, $^3J_{H\text{-}H}$ = 6.9 Hz, 12H, CH(C***H$_3$***)(CH$_3$)), 1.15 (d, $^3J_{H\text{-}H}$ = 7.0 Hz, 6H, CH(C***H$_3$***)(CH$_3$)), 0.73 (d, $^3J_{H\text{-}H}$ = 6.9 Hz, 6H, CH(C***H$_3$***)(CH$_3$)).

^{13}C NMR (C_6D_6, 298 K, 101 MHz) δ: 167.4 (2x N***C***(CH$_3$)), 167.2 (2x N***C***(CH$_3$)), 166.7 (2x N***C***(CH$_3$)), 166.2 (2x N***C***(CH$_3$)), 148.55 (d, $^1J_{C\text{-}C}$ = 78.3 Hz, $O\boldsymbol{^{13}C_1}H^1{=}^{13}C_2H^2H^3$), 147.1 (Ar-***C***), 145.7 (Ar-***C***), 142.6 (Ar-***C***), 142.6 (Ar-***C***), 141.5 (Ar-***C***), 140.8 (Ar-***C***), 125.0 (Ar-***C***H), 124.8 (Ar-***C***H), 124.5 (Ar-***C***H), 123.9 (Ar-***C***H), 123.1 (Ar-***C***H), 122.5 (Ar-***C***H), 93.49 (d, $^1J_{C\text{-}C}$ = 78.9 Hz, $O^{13}C_1H^1{=}\boldsymbol{^{13}C_2}H^2H^3$), 93.3 (2x ***C***H{C(CH$_3$)}$_2$), 92.0 (2x ***C***H{C(CH$_3$)}$_2$), 29.5 (2x ***C***H(CH$_3$)$_2$), 28.5 (2x ***C***H(CH$_3$)$_2$), 28.4 (2x ***C***H(CH$_3$)$_2$), 28.1 (2x ***C***H(CH$_3$)$_2$), 25.9 (4x CH(***C***H$_3$)(CH$_3$)), 24.4 (2x CH(***C***H$_3$)(CH$_3$)), 24.2 (2x CH(***C***H$_3$)(CH$_3$)), 24.0 (2x NC(***C***H$_3$)), 23.9 (2x NC(***C***H$_3$)), 23.8 (2x CH(***C***H$_3$)(CH$_3$)), 23.5 (2x CH(***C***H$_3$)(CH$_3$)), 23.2 (2x CH(***C***H$_3$)(CH$_3$)), 23.0 (2x CH(***C***H$_3$)(CH$_3$)), 20.1 (2x NC(***C***H$_3$)), 19.3 (2x NC(***C***H$_3$)). Some Ar***C*** resonances are overlapping and cannot be observed.

Preparation of 3a

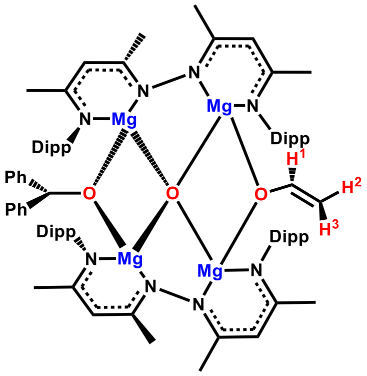

In a glovebox, **2** (20 mg, 0.017 mmol, 1 eq) and benzophenone (3.1 mg, 0.017 mmol, 1eq) were dissolved in C_6D_6 (0.6 mL) and transferred to a J. Young NMR tube. The reaction mixture was heated at 60 °C for 14 hours. A ^{1}H NMR spectrum was taken at this time point and showed the full conversion of **2** to **3a**. The J. Young NMR tube was returned to the glovebox, the reaction mixture was removed under vacuum and then solved in 1 mL Et_2O/n-pentane (1:1 v: v) mixture. The solution was filtered into a 4 mL vial and then placed in the glovebox freezer (–35 °C) for about 2 days. Yellow powder (**3a**) was successfully obtained. The filtrated powder was washed with cold n-pentane (3 x 1mL) and then dried in vacuo. (Several attempts were made to grow crystals in different solvents, but the crystal quality did not meet the test requirements due to weak diffraction.) **Yield: 9 mg,** 0.007 mmol, 40%.

^{1}H NMR (C_6D_6, 298 K, 400 MHz) δ: 7.13 – 6.73 (overlapping signals, 22H, Ar***H***), 5.39 (s, 1H, OC***H***(C_6H_5)$_2$), 4.94 (dd, $^3J_{H\text{-}H}$ = 13.7, 5.8 Hz, 1H, OC***H***^{1}=CH^{2}H^3), 4.77 (s, 1H, C***H***{C(CH$_3$)}$_2$), 4.75 (s, 1H, C***H***{C(CH$_3$)}$_2$), 4.68 (s, 1H, C***H***{C(CH$_3$)}$_2$), 4.67 (s, 1H, C***H***{C(CH$_3$)}$_2$), 3.30 (hept, $^3J_{H\text{-}H}$ = 6.9 Hz, 2H, C***H***(CH$_3$)$_2$), 3.28 (hept, $^3J_{H\text{-}H}$ = 6.9 Hz, 2H, C***H***(CH$_3$)$_2$), 3.24 (hept, $^3J_{H\text{-}H}$ = 6.9 Hz, 2H, C***H***(CH$_3$)$_2$), 3.20 (hept, $^3J_{H\text{-}H}$ = 6.9 Hz, 2H, C***H***(CH$_3$)$_2$), 3.14 (hept, $^3J_{H\text{-}H}$ = 6.9 Hz, 2H, C***H***(CH$_3$)$_2$), 3.12 (hept, $^3J_{H\text{-}H}$ = 6.9 Hz, 2H, C***H***(CH$_3$)$_2$), 2.97 (hept, $^3J_{H\text{-}H}$ = 6.9 Hz, 2H, C***H***(CH$_3$)$_2$), 2.96 (hept, $^3J_{H\text{-}H}$ = 6.9 Hz, 2H, C***H***(CH$_3$)$_2$), 2.93 (d, $^3J_{H\text{-}H}$ = 13.4 Hz, 1H, OCH^1=CH^2***H***^{3}), 2.87 (d, $^3J_{H\text{-}H}$ = 5.7 Hz, 1H, OCH^1=C***H***^{2}H^3), 2.00 (s, 3H, NC(C***H$_3$***)), 1.95 (s, 3H, NC(C***H$_3$***)), 1.89 (s, 3H, NC(C***H$_3$***)), 1.86 (s, 3H, NC(C***H$_3$***)), 1.72 (s, 3H, NC(C***H$_3$***)), 1.69 (s, 3H, NC(C***H$_3$***)), 1.59 (s, 3H, NC(C***H$_3$***)), 1.49 (s, 3H, NC(C***H$_3$***)), 1.49 (d, $^3J_{H\text{-}H}$ = 6.9 Hz, 3H, CH(C***H$_3$***)(CH$_3$)), 1.46 (d, $^3J_{H\text{-}H}$ = 6.9 Hz, 3H, CH(C***H$_3$***)(CH$_3$)), 1.33 (d, $^3J_{H\text{-}H}$ = 6.9 Hz, 3H, CH(C***H$_3$***)(CH$_3$)), 1.31 (d, $^3J_{H\text{-}H}$ = 6.9 Hz, 3H, CH(C***H$_3$***)(CH$_3$)), 1.28 (d, $^3J_{H\text{-}H}$ = 6.9 Hz, 3H, CH(C***H$_3$***)(CH$_3$)), 1.27 (d, $^3J_{H\text{-}H}$ = 6.9 Hz, 3H, CH(C***H$_3$***)(CH$_3$)), 1.25 (d, $^3J_{H\text{-}H}$ = 6.9 Hz, 3H, CH(C***H$_3$***)(CH$_3$)), 1.22 (d, $^3J_{H\text{-}H}$ = 7.0 Hz, 3H, CH(C***H$_3$***)(CH$_3$)), 1.21 (d, $^3J_{H\text{-}H}$ = 7.0 Hz, 3H, CH(C***H$_3$***)(CH$_3$)), 1.20 (d, $^3J_{H\text{-}H}$ = 7.0 Hz, 3H, CH(C***H$_3$***)(CH$_3$)), 1.19 (d, $^3J_{H\text{-}H}$ = 7.0 Hz, 3H, CH(C***H$_3$***)(CH$_3$)), 1.18 (d, $^3J_{H\text{-}H}$ = 7.0 Hz, 3H, CH(C***H$_3$***)(CH$_3$)), 1.16 (d, $^3J_{H\text{-}H}$ = 7.0 Hz, 3H, CH(C***H$_3$***)(CH$_3$)), 0.99 (d, $^3J_{H\text{-}H}$ = 6.9 Hz, 3H, CH(C***H$_3$***)(CH$_3$)), 0.78 (d, $^3J_{H\text{-}H}$ = 6.9 Hz, 3H, CH(C***H$_3$***)(CH$_3$)), 0.56 (d, $^3J_{H\text{-}H}$ = 6.9 Hz, 3H, CH(C***H$_3$***)(CH$_3$)).

^{13}C NMR (C_6D_6, 298 K, 101 MHz) δ: 169.3 (NC(**C**H_3)), 168.9 (NC(**C**H_3)), 167.1 (NC(**C**H_3)), 167.0 (NC(**C**H_3)), 166.9 (NC(**C**H_3)), 166.6 (NC(**C**H_3)), 165.3 (NC(**C**H_3)), 165.2 (NC(**C**H_3)), 149.4 (Ar-**C**), 148.5 (Ar-**C**), 148.3 (O**C**H^1=CH^2H^3), 147.8 (Ar-**C**), 147.5 (Ar-**C**), 146.0 (Ar-**C**), 145.0 (Ar-**C**), 144.0 (Ar-**C**), 143.1 (Ar-**C**), 143.0 (Ar-**C**), 142.8 (Ar-**C**), 142.5 (Ar-**C**), 141.5 (Ar-**C**), 141.3 (Ar-**C**), 141.1 (Ar-**C**), 127.2 (Ar-**C**H), 127.1 (Ar-**C**H), 126.3 (Ar-**C**H), 125.7 (Ar-**C**H), 125.5 (Ar-**C**H), 124.9 (Ar-**C**H), 124.8 (Ar-**C**H), 124.7 (Ar-**C**H), 124.4 (Ar-**C**H), 124.1 (Ar-**C**H), 123.8 (Ar-**C**H), 123.6 (Ar-**C**H), 123.2 (Ar-**C**H), 123.0 (Ar-**C**H), 122.9 (Ar-**C**H), 94.3 (**C**H{C(CH_3)}$_2$), 94.1 (**C**H{C(CH_3)}$_2$), 93.9 (**C**H{C(CH_3)}$_2$), 93.7 (**C**H{C(CH_3)}$_2$), 93.2 (OCH^1=**C**H^2H^3), 75.7 (O**C**H(C_6H_5)$_2$), 29.7 (**C**H(CH_3)$_2$), 29.1 (**C**H(CH_3)$_2$), 28.6 (**C**H(CH_3)$_2$), 28.6 (**C**H(CH_3)$_2$), 28.3 (**C**H(CH_3)$_2$), 28.1 (**C**H(CH_3)$_2$), 28.0 (**C**H(CH_3)$_2$), 27.9 (**C**H(CH_3)$_2$), 26.5 (NC(**C**H_3)), 26.0 (NC(**C**H_3)), 25.4 (CH(**C**H_3)(CH_3)), 25.1 (NC(**C**H_3)), 25.0 (CH(**C**H_3)(CH_3)), 24.9 (CH(**C**H_3)(CH_3)), 24.8 (CH(**C**H_3)(CH_3)), 24.7 (NC(**C**H_3)), 24.6 (CH(**C**H_3)(CH_3)), 24.6 (CH(**C**H_3)(CH_3)), 24.5 (CH(**C**H_3)(CH_3)), 24.4 (CH(**C**H_3)(CH_3)), 24.3 (CH(**C**H_3)(CH_3)), 24.2 (CH(**C**H_3)(CH_3)), 23.9 (CH(**C**H_3)(CH_3)), 23.7 (CH(**C**H_3)(CH_3)), 23.6 (CH(**C**H_3)(CH_3)), 23.5 (CH(**C**H_3)(CH_3)), 23.3 (CH(**C**H_3)(CH_3)), 22.7 (CH(**C**H_3)(CH_3)), 20.3 (NC(**C**H_3)), 20.2 (NC(**C**H_3)), 19.5 (NC(**C**H_3)), 19.0 (NC(**C**H_3)). Some Ar***CH*** and resonances are overlapping and cannot be observed.

Preparation of 3b

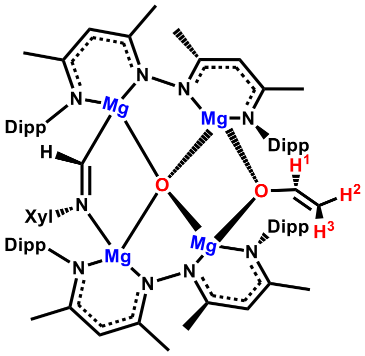

In a glovebox, **2** (20 mg, 0.017 mmol, 1 eq) and 2-isocyano-1,3-dimethylbenzene (2.2 mg, 0.017 mmol, 1eq) were dissolved in C_6D_6 (0.6 mL) and transferred to a J. Young NMR tube. The reaction mixture was heated at 60 °C for 14 hours. A ^{1}H NMR spectrum was taken at this time point and showed the full conversion of **2** to **3b**. The J. Young NMR tube was returned to the glovebox, the reaction mixture was removed under vacuum and then solved in 1 mL Et_2O/n-pentane (1:1 v: v) mixture. The solution was filtered into a 4 mL vial and then placed in the glovebox freezer (–35 °C) for about 2 days. Yellow powder (**3b**) was successfully obtained. The filtrated powder was washed with cold n-pentane (3 x 1mL) and then dried in vacuo. **Yield: 7 mg,** 0.005 mmol, 30%. Few bright yellow crystals for X-ray can be obtained from 1 mL Et_2O/n-pentane (2:1 v: v) mixture.

^{1}H NMR (C_6D_6, 298 K, 400 MHz) δ: 8.17 (s, 1H, C***H***=NXyl), 7.13 – 7.03 (overlapping signals, 6H, Ar***H***), 6.88 (dd, $^3J_{H-H}$ = 6.5, 2.8 Hz, 1H, Ar***H***), 6.81 (d, $^3J_{H-H}$ = 7.7 Hz, 1H, Ar***H***), 6.73 – 6.69 (overlapping signals, 4H, Ar***H***), 6.67 $^3J_{H-H}$ = 4.6 Hz, 1H, Ar***H***), 6.63 (t, $^3J_{H-H}$ = 7.7 Hz, 1H, Ar***H***), 6.57 (d, $^3J_{H-H}$ = 7.6 Hz, 1H, Ar***H***), 5.06 (dd, $^3J_{H-H}$ = 13.6, 5.7 Hz, 1H, OC$\boldsymbol{H^1}$=CH^2H^3), 4.78 (s, 1H, C***H***$\{C(CH_3)\}_2$), 4.76 (s, 1H, C***H***$\{C(CH_3)\}_2$), 4.76 (s, 1H, C***H***$\{C(CH_3)\}_2$), 4.70 (s, 1H, C***H***$\{C(CH_3)\}_2$), 3.35 (hept, $^3J_{H-H}$ = 6.8 Hz, 2H, C***H***$(CH_3)_2$), 3.29 (hept, $^3J_{H-H}$ = 6.8 Hz, 2H, C***H***$(CH_3)_2$), 3.25 (hept, $^3J_{H-H}$ = 6.8 Hz, 2H, C***H***$(CH_3)_2$), 3.15 (hept, $^3J_{H-H}$ = 6.8 Hz, 2H, C***H***$(CH_3)_2$), 3.13 (hept, $^3J_{H-H}$ = 6.8 Hz, 2H, C***H***$(CH_3)_2$), 3.12 (hept, $^3J_{H-H}$ = 6.8 Hz, 2H, C***H***$(CH_3)_2$), 3.11 (hept, $^3J_{H-H}$ = 6.8 Hz, 2H, C***H***$(CH_3)_2$), 2.96 (d, $^3J_{H-H}$ = 13.8 Hz, 1H, OCH^1=C$H^2\boldsymbol{H^3}$), 2.92 (d, $^3J_{H-H}$ = 5.7 Hz, 1H, OCH^1=C$\boldsymbol{H^2}H^3$), 2.90 (hept, $^3J_{H-H}$ = 6.8 Hz, 2H, C***H***$(CH_3)_2$), 2.07 (s, 3H, NXyl-C$\boldsymbol{H_3}$), 1.90 (s, 3H, NC(C$\boldsymbol{H_3}$)), 1.88 (s, 3H, NC(C$\boldsymbol{H_3}$)), 1.87 (s, 3H, NC(C$\boldsymbol{H_3}$)), 1.86 (s, 3H, NC(C$\boldsymbol{H_3}$)), 1.77 (s, 3H, NC(C$\boldsymbol{H_3}$)), 1.75 (s, 3H, NC(C$\boldsymbol{H_3}$)), 1.71 (s, 3H, NC(C$\boldsymbol{H_3}$)), 1.60 (s, 3H, NC(C$\boldsymbol{H_3}$)), 1.56 (d, $^3J_{H-H}$ = 6.9 Hz, 3H, CH(C$\boldsymbol{H_3}$)(CH_3)), 1.50 (d, $^3J_{H-H}$ = 6.8 Hz, 3H, CH(C$\boldsymbol{H_3}$)(CH_3)), 1.41 (d, $^3J_{H-H}$ = 7.0 Hz, 3H, CH(C$\boldsymbol{H_3}$)(CH_3)), 1.38 (d, $^3J_{H-H}$ = 6.9 Hz, 3H, CH(C$\boldsymbol{H_3}$)(CH_3)), 1.36 (d, $^3J_{H-H}$ = 6.8 Hz, 3H, CH(C$\boldsymbol{H_3}$)(CH_3)), 1.29 (d, $^3J_{H-H}$ = 6.9 Hz, 3H, CH(C$\boldsymbol{H_3}$)(CH_3)), 1.27 (d, $^3J_{H-H}$ = 6.8 Hz, 3H, CH(C$\boldsymbol{H_3}$)(CH_3)), 1.24 (d, $^3J_{H-H}$ = 6.9 Hz, 3H, CH(C$\boldsymbol{H_3}$)(CH_3)), 1.23 (d, $^3J_{H-H}$ = 6.9 Hz, 3H, CH(C$\boldsymbol{H_3}$)(CH_3)), 1.21 (d, $^3J_{H-H}$ = 6.9 Hz, 3H, CH(C$\boldsymbol{H_3}$)(CH_3)), 1.19 (d, $^3J_{H-H}$ = 6.9 Hz, 3H, CH(C$\boldsymbol{H_3}$)(CH_3)), 1.16 (d, $^3J_{H-H}$ = 6.9 Hz, 3H,

CH(C***H*₃**)(CH$_3$)), 1.15 (d, $^3J_{H-H}$ = 6.9 Hz, 6H, CH(C***H*₃**)(CH$_3$)), 1.11 (d, $^3J_{H-H}$ = 6.8 Hz, 3H, CH(C***H*₃**)(CH$_3$)), 1.04 (s, 3H, NXyl-C***H*₃**), 0.29 (d, $^3J_{H-H}$ = 6.8 Hz, 3H, CH(C***H*₃**)(CH$_3$)).

^{13}C NMR (C_6D_6, 298 K, 101 MHz) δ: 168.8 (N***C***(CH$_3$)), 168.6 (N***C***(CH$_3$)), 167.4 (N***C***(CH$_3$)), 166.3 (N***C***(CH$_3$)), 166.3 (N***C***(CH$_3$)), 165.73 (N***C***(CH$_3$)), 165.5 (N***C***(CH$_3$)), 164.9 (N***C***(CH$_3$)), 157.5 (***C***H=NXyl), 148.6 (O***C***H^1=CH^{2}H^3), 148.4 (Ar-***C***), 148.4 (Ar-***C***), 147.7 (Ar-***C***), 143.5 (Ar-***C***), 143.2 (Ar-***C***), 141.7 (Ar-***C***), 141.6 (Ar-***C***), 141.6 (Ar-***C***), 141.3 (Ar-***C***), 140.4 (Ar-***C***), 129.5 (Ar-***C***H), 129.2 (Ar-***C***H), 126.9 (Ar-***C***H), 124.9 (Ar-***C***H), 124.8 (Ar-***C***H), 124.7 (Ar-***C***H), 124.6 (Ar-***C***H), 124.5 (Ar-***C***H), 124.4 (Ar-***C***H), 124.3 (Ar-***C***H), 123.6 (Ar-***C***H), 123.2 (Ar-***C***H), 123.1 (Ar-***C***H), 122.9 (Ar-***C***H), 122.8 (Ar-***C***H), 94.5 (***C***H{C(CH$_3$)}$_2$), 94.1 (***C***H{C(CH$_3$)}$_2$), 94.0 (***C***H{C(CH$_3$)}$_2$), 93.9 (***C***H{C(CH$_3$)}$_2$), 93.1 (OCH^1=***C***H^{2}H^3), 29.6 (***C***H(CH$_3$)$_2$), 29.4 (***C***H(CH$_3$)$_2$), 29.3 (***C***H(CH$_3$)$_2$), 29.0 (***C***H(CH$_3$)$_2$), 28.5 (***C***H(CH$_3$)$_2$), 28.0 (***C***H(CH$_3$)$_2$), 27.8 (2x ***C***H(CH$_3$)$_2$), 26.0 (NC(***C***H$_3$)), 25.8 (NC(***C***H$_3$)), 25.3 (NC(***C***H$_3$)), 25.2 (NC(***C***H$_3$)), 24.9 (CH(***C***H$_3$)(CH$_3$)), 24.8 (CH(***C***H$_3$)(CH$_3$)), 24.7 (CH(***C***H$_3$)(CH$_3$)), 24.6 (CH(***C***H$_3$)(CH$_3$)), 24.6 (CH(***C***H$_3$)(CH$_3$)), 24.5 (CH(***C***H$_3$)(CH$_3$)), 24.4 (CH(***C***H$_3$)(CH$_3$)), 24.2 (CH(***C***H$_3$)(CH$_3$)), 24.1 (CH(***C***H$_3$)(CH$_3$)), 24.0 (CH(***C***H$_3$)(CH$_3$)), 23.9 (CH(***C***H$_3$)(CH$_3$)), 23.8 (CH(***C***H$_3$)(CH$_3$)), 23.8 (CH(***C***H$_3$)(CH$_3$)), 23.6 (CH(***C***H$_3$)(CH$_3$)), 22.7 (CH(***C***H$_3$)(CH$_3$)), 22.6 (CH(***C***H$_3$)(CH$_3$)), 20.2 (NXyl-***C***H$_3$), 19.9 (NC(***C***H$_3$)), 19.7 (NC(***C***H$_3$)), 19.5 (NC(***C***H$_3$)), 18.6 (NC(***C***H$_3$)), 17.8 (NXyl-***C***H$_3$). Some Ar***CH*** and resonances are overlapping and cannot be observed.

Preparation of 3c

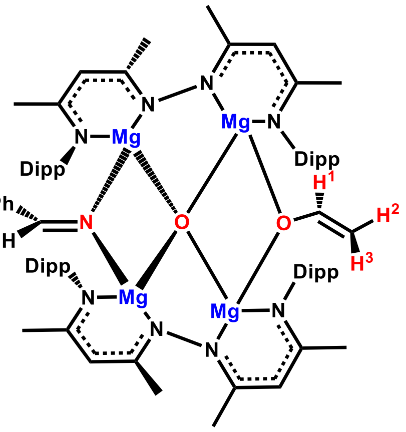

In a glovebox, **2** (20 mg, 0.017 mmol, 1 eq) and benzonitrile (1.8 mg, 0.018 mmol, 1.05 eq) were dissolved in C_6D_6 (0.6 mL) and transferred to a J. Young NMR tube. The reaction mixture was heated at 60 °C for 3 hours. A ^{1}H NMR spectrum was taken at this time point and showed the full conversion of **2** to **3c**. The J. Young NMR tube was returned to the glovebox, the reaction mixture was removed under vacuum and then solved in 1 mL Et_2O/n-pentane (1:5 v: v) mixture. The solution was filtered into a 4 mL vial and then placed in the glovebox freezer (–35 °C) for about 5 days. Yellow powder (**3c**) was successfully obtained. The filtrated powder was washed with cold n-pentane (3 x 1mL) and then dried in vacuo. (Several attempts were made to grow crystals in different solvents, but the crystal quality did not meet the test requirements due to weak diffraction.) **Yield: 7 mg,** 0.005 mmol, 30%.

^{1}H NMR (C_6D_6, 298 K, 400 MHz) δ: 7.98 (s, 1H, N=C***H***Ph), 7.09 – 7.06 (overlapping signals, 6H, Ar***H***), 7.05 – 7.00 (overlapping signals, 6H, Ar***H***), 6.94 (m, 1H, Ar***H***), 6.88 – 6.84 (m, 2H, Ar***H***), 6.82 – 6.79 (m, 2H, Ar***H***), 5.02 (dd, $^3J_{H\text{-}H}$ = 13.8, 5.6 Hz, 1H, OC***H***^{1}=CH^{2}H^3), 4.75 (s, 2H, C***H***{C(CH$_3$)}$_2$), 4.71 (s, 2H, C***H***{C(CH$_3$)}$_2$), 3.22 (hept, $^3J_{H\text{-}H}$ = 7.0 Hz, 2H, C***H***(CH$_3$)$_2$), 3.17 (hept, $^3J_{H\text{-}H}$ = 7.0 Hz, 2H, C***H***(CH$_3$)$_2$), 3.06 (hept, $^3J_{H\text{-}H}$ = 6.9 Hz, 2H, C***H***(CH$_3$)$_2$), 2.96 (d, $^3J_{H\text{-}H}$ = 13.6 Hz, 1H, OCH^1=CH^2***H***^{3}), 2.94 (hept, $^3J_{H\text{-}H}$ = 6.9 Hz, 2H, C***H***(CH$_3$)$_2$), 2.93 (d, $^3J_{H\text{-}H}$ = 5.4 Hz, 1H, OCH^1=C***H***^{2}H^3), 1.98 (s, 6H, NC(C***H***$_{3}$)), 1.92 (s, 6H, NC(C***H***$_{3}$)), 1.71 (s, 6H, NC(C***H***$_{3}$)), 1.66 (s, 6H, NC(C***H***$_{3}$)), 1.54 (d, $^3J_{H\text{-}H}$ = 6.8 Hz, 6H, CH(C***H***$_{3}$)(CH$_3$)), 1.28 (d, $^3J_{H\text{-}H}$ = 7.0 Hz, 6H, CH(C***H***$_{3}$)(CH$_3$)), 1.26 (d, $^3J_{H\text{-}H}$ = 7.0 Hz, 6H, CH(C***H***$_{3}$)(CH$_3$)), 1.22 (d, $^3J_{H\text{-}H}$ = 7.0 Hz, 6H, CH(C***H***$_{3}$)(CH$_3$)), 1.21 (d, $^3J_{H\text{-}H}$ = 6.8 Hz, 6H, CH(C***H***$_{3}$)(CH$_3$)), 1.19 (d, $^3J_{H\text{-}H}$ = 6.8 Hz, 6H, CH(C***H***$_{3}$)(CH$_3$)), 1.14 (d, $^3J_{H\text{-}H}$ = 6.8 Hz, 6H, CH(C***H***$_{3}$)(CH$_3$)), 0.77 (d, $^3J_{H\text{-}H}$ = 6.8 Hz, 6H, CH(C***H***$_{3}$)(CH$_3$)).

^{13}C NMR (C_6D_6, 298 K, 101 MHz) δ: 176.3 (N=***C***HPh), 167.6 (N***C***(CH$_3$)), 167.4 (N***C***(CH$_3$)), 165.9 (N***C***(CH$_3$)), 165.4 (N***C***(CH$_3$)), 148.9 (O***C***H^1=CH^{2}H^3), 147.9 (Ar-***C***), 147.8 (Ar-***C***), 143.0 (Ar-***C***), 142.1 (Ar-***C***), 141.2 (Ar-***C***), 141.0 (Ar-***C***), 140.0 (Ar-***C***), 129.9 (Ar-***C***H), 124.7 (Ar-***C***H), 124.5 (Ar-***C***H), 124.4 (Ar-***C***H), 123.8 (Ar-***C***H), 118.9 (Ar-***C***H), 112.6 (Ar-***C***H), 93.5 (***C***H{C(CH$_3$)}$_2$), 93.3

(***C***H{C(CH_3)}$_2$), 93.2 (OCH^1=***C***H^{2}H^3), 29.7 (***C***H(CH_3)$_2$), 29.4 (***C***H(CH_3)$_2$), 28.1 (***C***H(CH_3)$_2$), 28.0 (***C***H(CH_3)$_2$), 25.1 (CH(***C***H$_3$)(CH_3)), 24.9 (CH(***C***H$_3$)(CH_3)), 24.6 (NC(***C***H$_3$)), 24.4 (NC(***C***H$_3$)), 24.3 (CH(***C***H$_3$)(CH_3)), 24.2 (CH(***C***H$_3$)(CH_3)), 24.1 (CH(***C***H$_3$)(CH_3)), 23.7 (CH(***C***H$_3$)(CH_3)), 23.5 (CH(***C***H$_3$)(CH_3)), 22.7 (CH(***C***H$_3$)(CH_3)), 19.9 (NC(***C***H$_3$)), 19.8 (NC(***C***H$_3$)).

Preparation of 3d

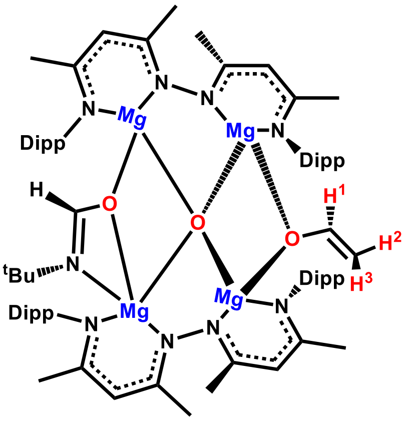

In a glovebox, **2** (20 mg, 0.017 mmol, 1 eq) and tert-butyl isocyanate (21.8 mg, 0.018 mmol, 1.05 eq) were dissolved in C_6D_6 (0.6 mL) and transferred to a J. Young NMR tube. The reaction mixture was stored at 25 °C for 15 hours. A ^{1}H NMR spectrum was taken at this time point and showed the full conversion of **2** to **3d**. The J. Young NMR tube was returned to the glovebox, the reaction mixture was removed under vacuum and then solved in 1 mL Et_2O/n-pentane (1:5 v: v) mixture. The solution was filtered into a 4 mL vial and then placed in the glovebox freezer (–35 °C) for about 2 days. Yellow powder (**3d**) was successfully obtained. The filtrated powder was washed with cold n-pentane (3 x 1mL) and then dried in vacuo. (Several attempts were made to grow crystals in different solvents, but the crystal quality did not meet the test requirements due to weak diffraction.) **Yield: 7 mg,** 0.005 mmol, 30%.

^{1}H NMR (C_6D_6, 298 K, 400 MHz) δ: 7.13 – 6.99 (overlapping signals, 12H, Ar***H***), 6.98 (s, 1H, NC***H***O), 5.45 (dd, $^3J_{H\text{-}H}$ = 13.7, 5.7 Hz, 1H, OC***H¹***=CH²H³), 5.17 (s, 1H, C***H***{C(CH$_3$)}$_2$), 4.76 (s, 1H, C***H***{C(CH$_3$)}$_2$), 4.72 (s, 1H, C***H***{C(CH$_3$)}$_2$), 4.62 (s, 1H, C***H***{C(CH$_3$)}$_2$), 3.55 (hept, $^3J_{H\text{-}H}$ = 6.7 Hz, 1H, C***H***(CH$_3$)$_2$), 3.54 (hept, $^3J_{H\text{-}H}$ = 6.7 Hz, 1H, C***H***(CH$_3$)$_2$), 3.39 (hept, $^3J_{H\text{-}H}$ = 6.7 Hz, 1H, C***H***(CH$_3$)$_2$), 3.38 (hept, $^3J_{H\text{-}H}$ = 6.7 Hz, 1H, C***H***(CH$_3$)$_2$), 3.36 (hept, $^3J_{H\text{-}H}$ = 6.7 Hz, 1H, C***H***(CH$_3$)$_2$), 3.35 (hept, $^3J_{H\text{-}H}$ = 6.7 Hz, 1H, C***H***(CH$_3$)$_2$), 3.08 (hept, $^3J_{H\text{-}H}$ = 6.7 Hz, 1H, C***H***(CH$_3$)$_2$), 2.96 (hept, $^3J_{H\text{-}H}$ = 6.7 Hz, 1H, C***H***(CH$_3$)$_2$), 2.98 (d, $^3J_{H\text{-}H}$ = 5.7 Hz, 1H, OCH¹=C***H²***H³), 2.58 (d, $^3J_{H\text{-}H}$ = 13.8 Hz, 1H, OCH¹=CH²***H³***), 2.29 (s, 3H, NC(C***H₃***)), 2.00 (s, 3H, NC(C***H₃***)), 1.93 (s, 3H, NC(C***H₃***)), 1.92 (s, 3H, NC(C***H₃***)), 1.87 (s, 3H, NC(C***H₃***)), 1.77 (s, 3H, NC(C***H₃***)), 1.72 (s, 3H, NC(C***H₃***)), 1.68 (d, $^3J_{H\text{-}H}$ = 7.1 Hz, 3H, CH(C***H₃***)(CH$_3$)), 1.62 (s, 3H, NC(C***H₃***)), 1.54 (d, $^3J_{H\text{-}H}$ = 7.0 Hz, 3H, CH(C***H₃***)(CH$_3$)), 1.45 (s, 9H, C(C***H₃***)$_3$), 1.42 (d, $^3J_{H\text{-}H}$ = 6.8 Hz, 3H, CH(C***H₃***)(CH$_3$)), 1.36 (d, $^3J_{H\text{-}H}$ = 7.2 Hz, 3H, CH(C***H₃***)(CH$_3$)), 1.34 (d, $^3J_{H\text{-}H}$ = 7.0 Hz, 3H, CH(C***H₃***)(CH$_3$)), 1.30 (d, $^3J_{H\text{-}H}$ = 7.0 Hz, 3H, CH(C***H₃***)(CH$_3$)), 1.30 (d, $^3J_{H\text{-}H}$ = 7.0 Hz, 3H, CH(C***H₃***)(CH$_3$)), 1.28 (d, $^3J_{H\text{-}H}$ = 6.8 Hz, 3H, CH(C***H₃***)(CH$_3$)), 1.24 (d, $^3J_{H\text{-}H}$ = 6.9 Hz, 3H, CH(C***H₃***)(CH$_3$)), 1.17 (d, $^3J_{H\text{-}H}$ = 6.9 Hz, 3H, CH(C***H₃***)(CH$_3$)), 1.15 (d, $^3J_{H\text{-}H}$ = 6.8 Hz, 3H, CH(C***H₃***)(CH$_3$)), 1.13 (d, $^3J_{H\text{-}H}$ = 6.8 Hz, 3H, CH(C***H₃***)(CH$_3$)), 1.10 (d, $^3J_{H\text{-}H}$ = 7.0 Hz, 3H, CH(C***H₃***)(CH$_3$)), 1.08 (d, $^3J_{H\text{-}H}$ = 7.0 Hz, 3H, CH(C***H₃***)(CH$_3$)).

^{13}C NMR (101 MHz, C_6D_6, 298 K) δ: 175.7 (N***C***HO), 169.3 (N***C***(CH$_3$)), 168.5 (N***C***(CH$_3$)), 167.4

(N***C***(CH_3)), 166.9 (N***C***(CH_3)), 164.9 (N***C***(CH_3)), 164.1 (N***C***(CH_3)), 163.7 (N***C***(CH_3)), 161.2 (N***C***(CH_3)), 156.0 (Ar-***C***), 151.5 (O***C***H^1=CH^2H^3), 150.0 (Ar-***C***), 146.7 (Ar-***C***), 146.2 (Ar-***C***), 145.6 (Ar-***C***), 144.2 (Ar-***C***), 142.8 (Ar-***C***), 142.7 (Ar-***C***), 141.9 (Ar-***C***), 141.2 (Ar-***C***), 140.8 (Ar-***C***), 137.3 (Ar-***C***), 136.0 (Ar-***C***), 135.5 (Ar-***C***), 129.8 (Ar-***C***H), 125.2 (Ar-***C***H), 124.8 (Ar-***C***H), 124.5 (Ar-***C***H), 124.4 (Ar-***C***H), 124.1 (Ar-***C***H), 124.0 (Ar-***C***H), 123.9 (Ar-***C***H), 123.5 (Ar-***C***H), 123.4 (Ar-***C***H), 123.2 (Ar-***C***H), 94.1 (***C***H{C(CH_3)}$_2$), 92.5 (***C***H{C(CH_3)}$_2$), 92.3 (***C***H{C(CH_3)}$_2$), 91.1 (OCH^1=***C***H^2H^3), 69.0 (***C***H{C(CH_3)}$_2$), 53.9 ((CH_3)***C***NCHO), 31.2 (***C***H(CH_3)$_2$), 28.9 (***C***H(CH_3)$_2$), 28.1 (***C***H(CH_3)$_2$), 27.9 (***C***H(CH_3)$_2$), 27.8 (***C***H(CH_3)$_2$), 27.7 (***C***H(CH_3)$_2$), 27.5 (***C***H(CH_3)$_2$), 26.1 (***C***H(CH_3)$_2$), 25.5 (NC(***C***H_3)), 25.0 (NC(***C***H_3)), 24.9 (NC(***C***H_3)), 24.9 (NC(***C***H_3)), 24.8 (CH(***C***H_3)(CH_3)), 24.7 (CH(***C***H_3)(CH_3)), 24.7 (CH(***C***H_3)(CH_3)), 24.6 (CH(***C***H_3)(CH_3)), 24.6 (CH(***C***H_3)(CH_3)), 24.5 (CH(***C***H_3)(CH_3)), 24.3 (CH(***C***H_3)(CH_3)), 23.9 (CH(***C***H_3)(CH_3)), 23.8 (CH(***C***H_3)(CH_3)), 23.6 (CH(***C***H_3)(CH_3)), 23.4 (CH(***C***H_3)(CH_3)), 23.3 (CH(***C***H_3)(CH_3)), 23.2 (CH(***C***H_3)(CH_3)), 22.7 (CH(***C***H_3)(CH_3)), 22.6 (CH(***C***H_3)(CH_3)), 20.7 (CH(***C***H_3)(CH_3)), 20.7 (NC(***C***H_3)), 20.2 (NC(***C***H_3)), 19.5 (NC(***C***H_3)), 18.1 (NC(***C***H_3)), 14.3 ((C***H***$_3$)CNCHO). Some Ar***CH*** and resonances are overlapping and cannot be observed.

2.2 Preparation of ^{13}C Labelled Samples

In order to unequivocally assign chemical shifts for the carbon environments, ^{13}C labelled samples of **2-$^{13}C_2$** was synthesized.

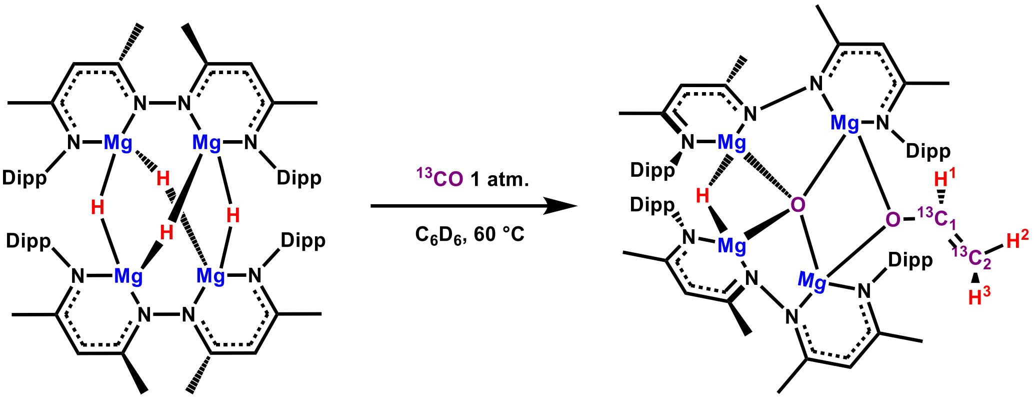

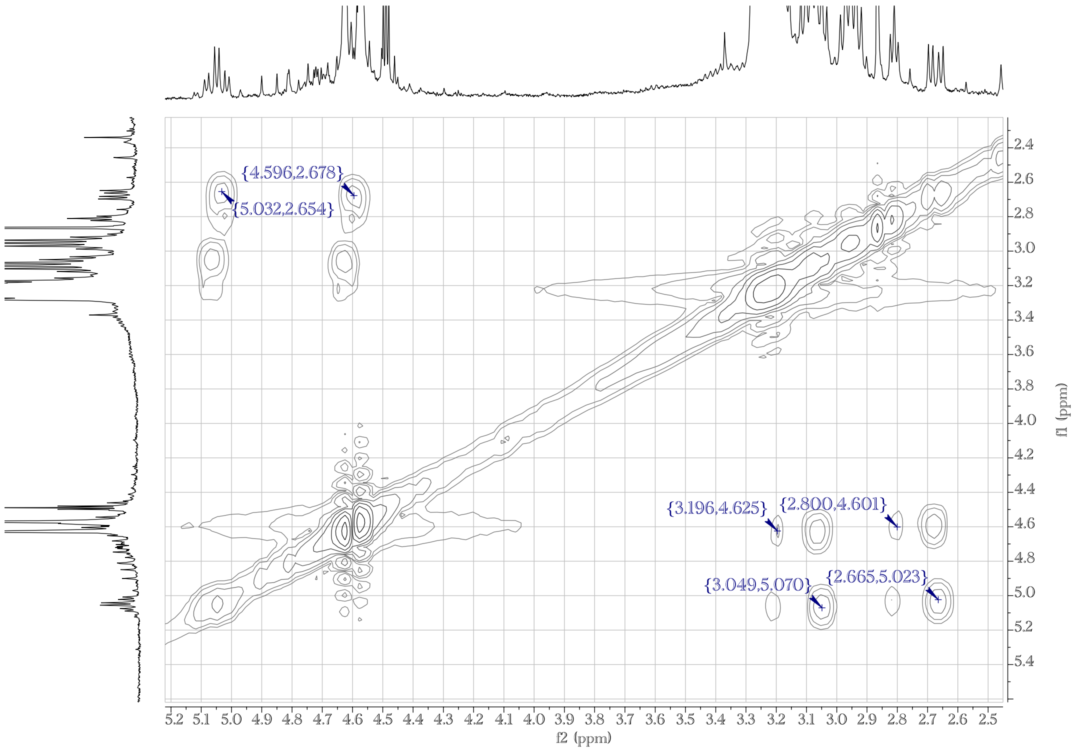

Figure S1. ^{1}H-^{1}H COSY spectrum of **2-$^{13}C_2$** with key ^{1}H resonances associated with C^1 and C^2.

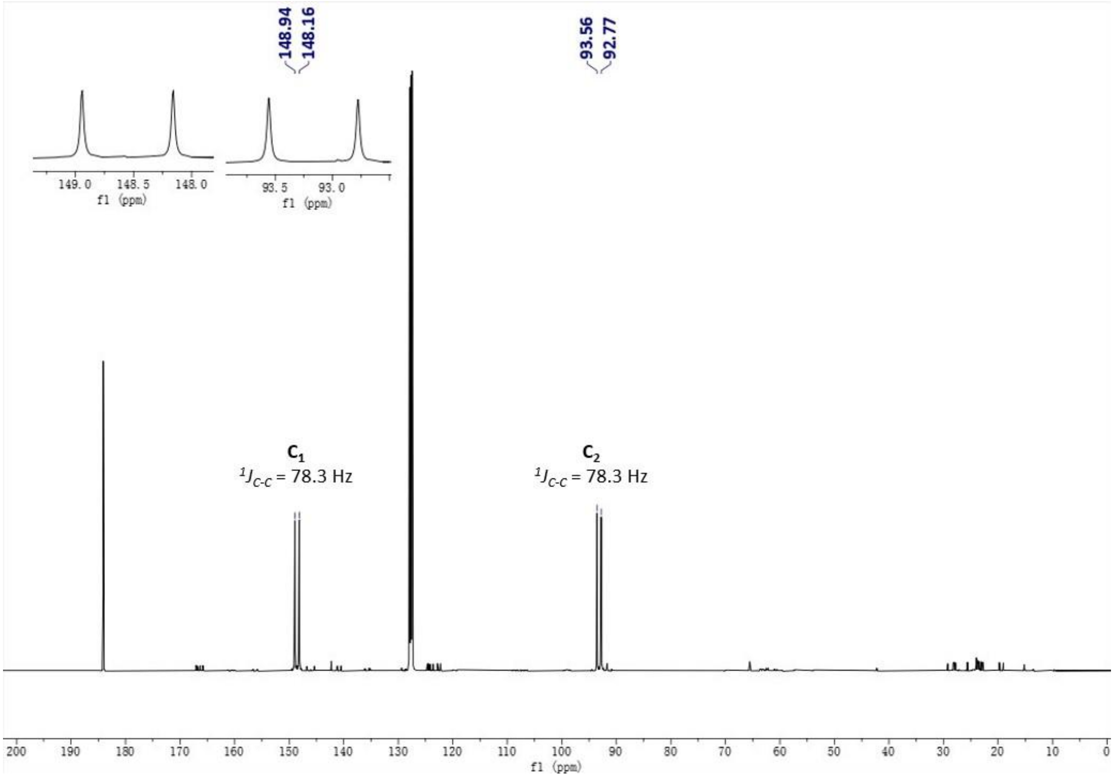

Figure S2. $^{13}C\{^1H\}$ spectrum of **2-$^{13}C_2$** with key ^{13}C resonances of C_1 and C_2.

3) Single Crystal X-ray Diffraction Data

- **The X-ray crystal structure of 2**

Crystal data for **2**: $C_{70}H_{100}Mg_4N_8O_2 \cdot C_4H_{10}O$, M = 1256.93, tetragonal, $P4_32_12$ (no. 96), $a = b$ = 15.5191(3), c = 31.4382(10) Å, V = 7571.7(4) Å^3, Z = 4 [C_2 symmetry], D_c = 1.103 g cm^{-3}, μ(Cu-Kα) = 0.817 mm^{-1}, T = 173 K, yellow blocks, Agilent Xcalibur PX Ultra A diffractometer; 7597 independent measured reflections (R_{int} = 0.1033), F^2 refinement,[S2-S4] R_1(obs) = 0.0644, wR_2(all) = 0.1784, 6785 independent observed absorption-corrected reflections [$|F_o| > 4\sigma(|F_o|)$, completeness to θ_{full}(67.7°) = 100%], 492 parameters. The structure was modelled as a two component racemic twin [Flack parameter x^+ = +0.21(9)]. CCDC 2281296.

The structure of **2** was found to sit across a C_2 axis that passes through O40 and bisects the Mg1···Mg1A and Mg2···Mg2A vectors. As a consequence the H1 hydrogen atom and the O41-based O–C(H)=CH_2 group are inherently disordered, and so the unique sites were refined at 50% occupancy. The C(H)=CH_2 portion of the latter was found to be further disordered. Two orientations were identified of *ca*. 28 and 22% occupancy, their geometries were optimised, the thermal parameters of adjacent atoms were restrained to be similar, and all of atoms were refined isotropically (O41 itself was refined anisotropically). The two unique 2,6-diisopropylphenyl groups (based on C11- and C23-) were both found to be disordered, and two orientations were identified in each case of *ca*. 53:47 and 73:27% occupancy respectively. The geometries of each pair of orientations were optimised, the thermal parameters of adjacent atoms were restrained to be similar, and only the non-hydrogen atoms of the major occupancy orientations were refined anisotropically (those of the minor occupancy orientations were refined isotropically). The O50-based included diethylether solvent molecule was found to be disordered across a C_2 axis, and two unique orientations were identified of *ca*. 37 and 13% occupancy (with two further orientations of the same occupancies being generated by operation of the C_2 axis). The geometries of the two unique orientations were optimised, the thermal parameters of adjacent atoms were restrained to be similar, and all of the atoms were refined isotropically. The H1 bridging hydride atom was located from a ΔF map and refined freely, but as its site is approximately the same as O41 transformed by the C_2 axis, it should be treated with more than usual caution.

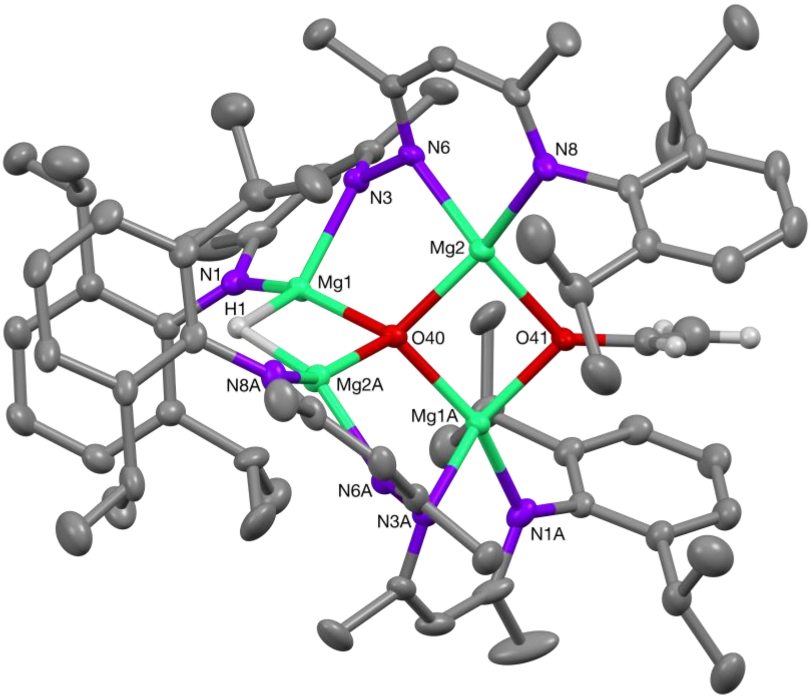

Fig. S3. The crystal structure of **2** (20% probability ellipsoids).

- **The X-ray crystal structure of 3b**

Crystal data for **3b**: $C_{79}H_{108}Mg_4N_9O_2{\cdot}C_4H_{10}O$, M = 1387.10, orthorhombic, $P2_12_12_1$ (no. 19), a = 15.8951(3), b = 17.8150(4), c = 29.6707(6) Å, V = 8401.9(3) Å^3, Z = 4, D_c = 1.097 g cm^{-3}, μ(Cu-Kα) = 0.783 mm^{-1}, T = 173 K, yellow blocks, Agilent Xcalibur PX Ultra A diffractometer; 33049 independent measured reflections (R_{int} = 0.0628), F^2 refinement,[S2-S4] R_1(obs) = 0.0508, wR_2(all) = 0.1156, 18703 independent observed absorption-corrected reflections [$|F_o| > 4\sigma(|F_o|)$, completeness to θ_{full}(67.7°) = 99.3%], 919 parameters. The absolute structure of **3b** was determined by use of the Flack parameter [x^+ = –0.06(3)]. CCDC 2312626.

The crystals of **3b** were found to be badly twinned. The major orientation in the crystal studied was found to use only *ca*. 33% of the observed spots, the second orientation *ca*. 31%, the third *ca*. 10% and the fourth *ca*. 7% (and this still left *ca*. 19% unindexed). The best results came from modelling just the first two orientations, using *ca*. 64% of the spots in total. This resulted in a two component twin in a *ca*. 47:53 ratio, with the two lattices related by the approximate twin law [0.98 0.00 0.10 –0.01 1.00 0.02 –0.35 –0.07 0.98]. The C17-based isopropyl group, the C63-based 2,6-diisopropylphenyl moiety, and the O81-based O–C(H)=CH_2 ligand were all found to be disordered, and in each case two orientations were identified, of *ca*. 61:39, 65:35 and 72:28% occupancy respectively. The geometries of each pair of orientations were optimised, the thermal parameters of adjacent atoms were restrained to be similar, and only the non-hydrogen atoms of the major occupancy orientations were refined anisotropically (those of the minor occupancy orientations were refined isotropically). The included solvent was found to be highly disordered, and the best approach to handling this diffuse electron density was found to be the SQUEEZE routine of PLATON.[S5] This suggested a total of 179 electrons per unit cell, equivalent to 44.8 electrons per complex. Before the use of SQUEEZE the solvent most resembled diethylether ($C_4H_{10}O$, 42 electrons), though the difference between this and the other solvent used (pentane) is very slight. One diethylether molecule corresponds to 42 electrons, so this was used as the solvent present. As a result, the atom list for the asymmetric unit is low by $C_4H_{10}O$ (and that for the unit cell low by $C_{16}H_{40}O_4$) compared to what is actually presumed to be present.

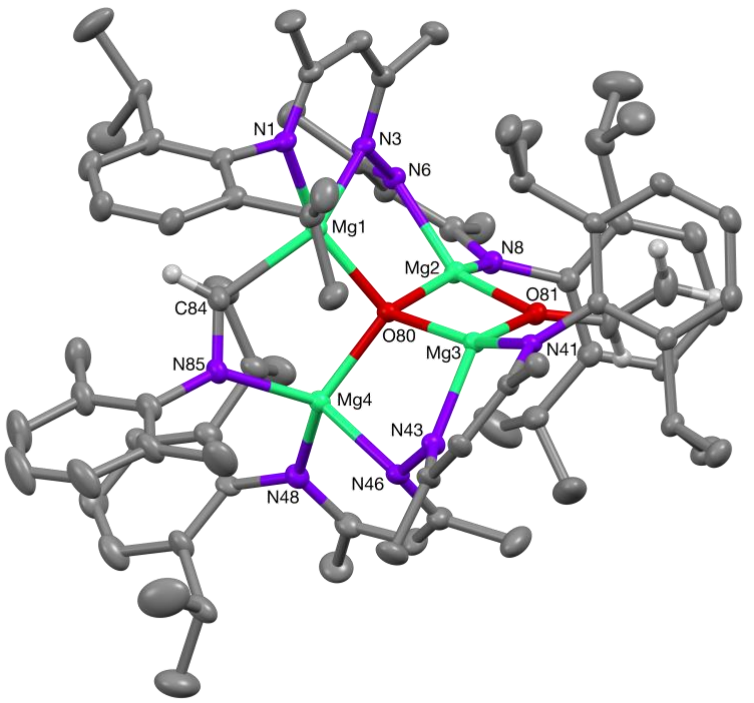

Fig. S4 The crystal structure of **3b** (20% probability ellipsoids).

4) DFT Studies

4.1. Computational methods

DFT calculations were performed using Gaussian 09 (Revision D.01) using an ultrafine integration grid (int=ultrafine).[S6] Geometry optimisations and frequency calculations were performed using the ωB97X-D density functionals with SDDAll (Mg), 6-31G** (C, H) and 6-311+G* (N, O) basis set. Frequency analyses for all stationary points were performed using the enhanced criteria to confirm the nature of the structures as either minima (no imaginary frequency) or transition states (only one imaginary frequency). The electronic energies of the optimised geometries were calculated using the ωB97X-D functional with 6-311+G** basis sets for all atoms with solvent corrections (PCM, benzene, ε = 2.2706). The Gibbs free energy correction from the frequency calculation was added to this electronic energy to generate Gibbs free energy values for the calculated stationary points.

Intrinsic reaction coordinate (IRC) calculations were used to connect transition states and minima located on the potential energy surface allowing a full energy profile (calculated at 298.15 K, 1 atm.) of the reaction to be constructed.

NBO analysis was performed at the ωB97X/6-311+G** level for all atoms with solvent corrections (PCM, benzene, ε = 2.2706) by using NBO 6.0.[S7] QTAIM analysis was conducted using the AIMAll software. [S8-S9]

Compound **1** possesses stereochemistry. The dinucleating ligand contains an axis along the N–N bond. When bound to the cluster this can create a chiral axis, reminiscent of that found in BINAP. With two ligands bound there is the possibility to generate a series of distinct stereoisomers. While it has been previously shown that epimerisation and interconversion of these isomer is facile for **1**,[S1] we did not assume this was the case throughout the mechanism in the reaction with CO. Rather a single stereoisomer is used consistently through the pathway, with **2** used as an input geometry for the model.

4.2. Comparison of Calculated and Solid-State Data

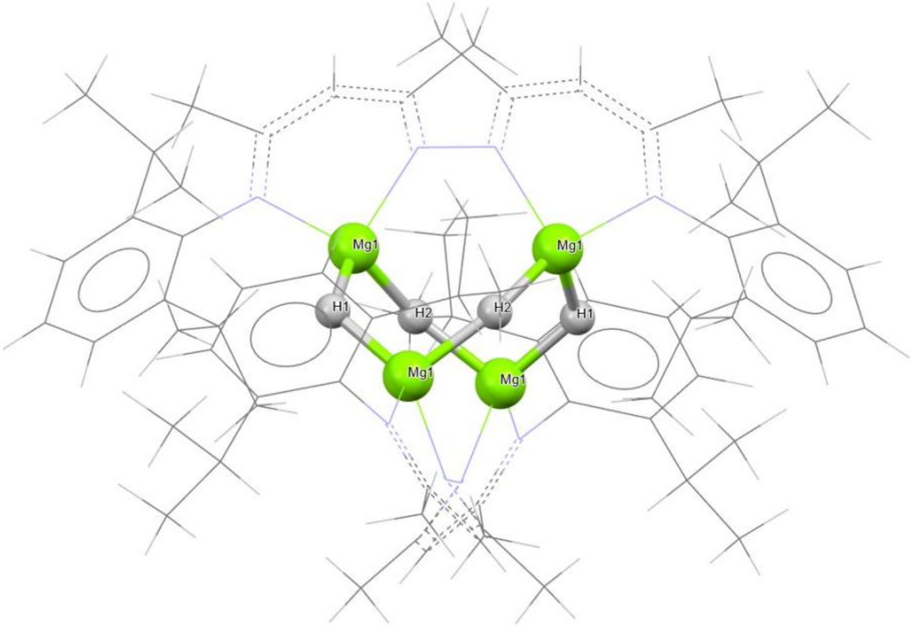

	1		Maximum Error
	Exp.	Calc.	
**Mg_1-H_1**	1.771	1.824	3.0%
**Mg_1-H_2**	1.845	1.834	0.6%
**Mg_1---Mg_1**	3.492	3.420	2.0%
**Mg_1---Mg_1**	3.030	3.026	0.1%
**$\angle Mg_1$-H_1–Mg_1**	117.66	112.13	4.7%
**$\angle Mg_1$-H_2–Mg_1**	152.71	157.30	3.0%

Table S1. Comparison of Calculated and Solid-State Data of **1**

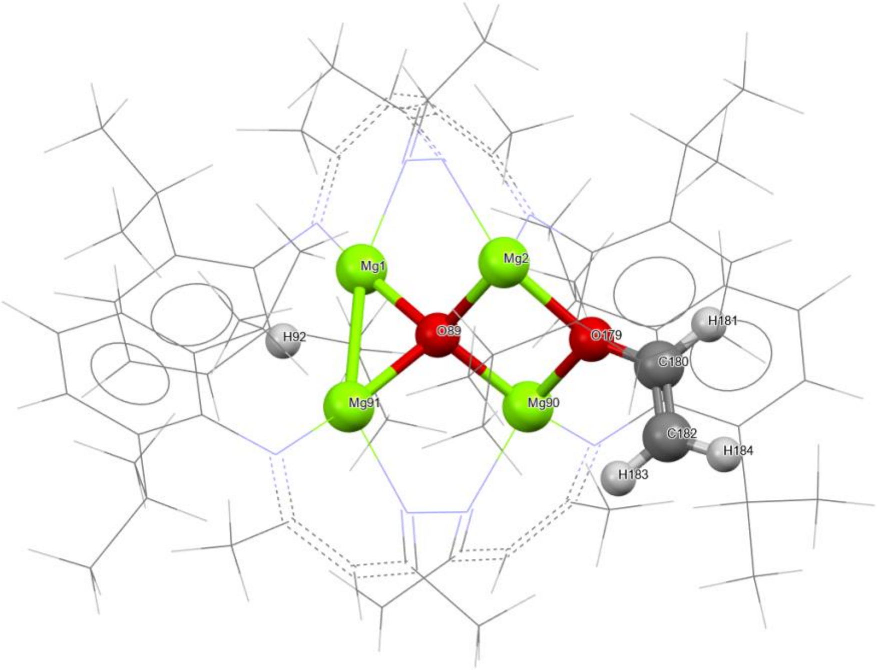

	2		Maximum Error
	**Exp.**	**Calc.**	
**Mg_{91}-H_{92}**	1.744	1.869	7.2%
**Mg_{1}-H_{92}**	1.958	1.875	4.2%
**Mg_{91}-O_{89}**	1.995	1.999	0.2%
**Mg_{1}-O_{89}**	1.997	1.988	0.5%
**Mg_{90}-O_{89}**	1.997	2.007	0.5%
**Mg_{2}-O_{89}**	1.995	2.000	0.3%
**Mg_{90}-O_{179}**	1.943	1.984	2.1%
**Mg_{2}-O_{179}**	2.021	2.004	0.8%
**C_{180}-O_{179}**	1.353	1.345	0.6%
**C_{180}-C_{182}**	1.288	1.336	3.7%
**$\angle Mg_{91}$-H_{92}–Mg_{1}**	100.96	97.47	3.5%
**$\angle Mg_{91}$-O_{89}–Mg_{1}**	91.48	89.78	1.9%
**$\angle Mg_{90}$-O_{89}–Mg_{2}**	91.48	95.05	3.9%
**$\angle Mg_{90}$-O_{179}–Mg_{2}**	92.30	95.65	3.6%
**$\angle Mg_{90}$-O_{179}–C_{180}**	121.65	131.69	8.3%
**$\angle Mg_{2}$-O_{179}–C_{180}**	138.73	133.97	3.4%
**$\angle O_{179}$-C_{180}–C_{182}**	121.46	124.35	2.4%

Table S2. Comparison of Calculated and Solid-State Data of **1**

4.3. Calculated Reaction Pathway

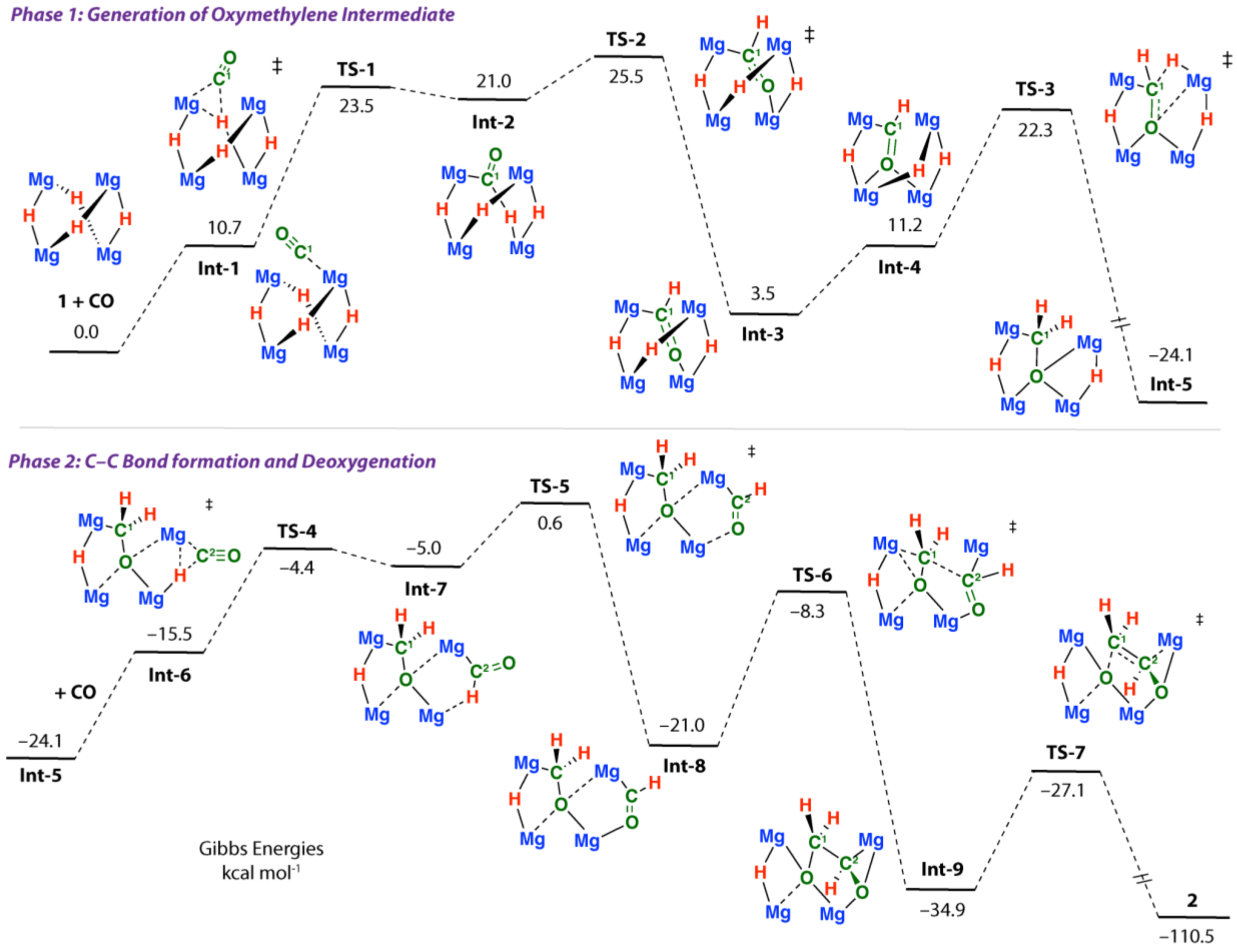

Figure S5. Calculated the reaction pathway of forming compound **2**. All energies in kcal mol^{-1}

4.4. Optimised structures

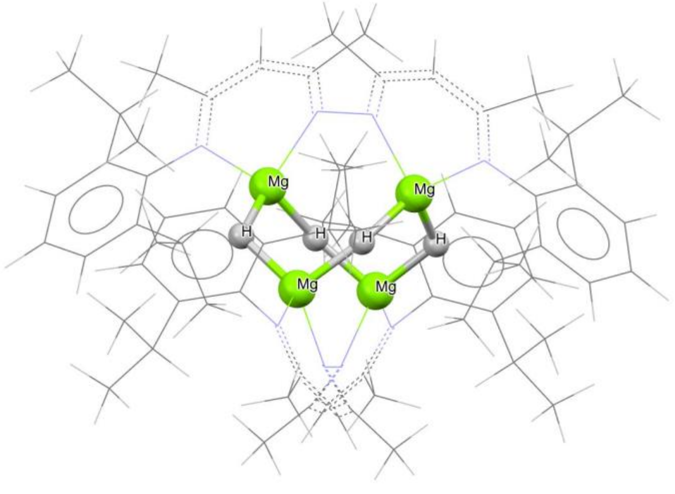

1

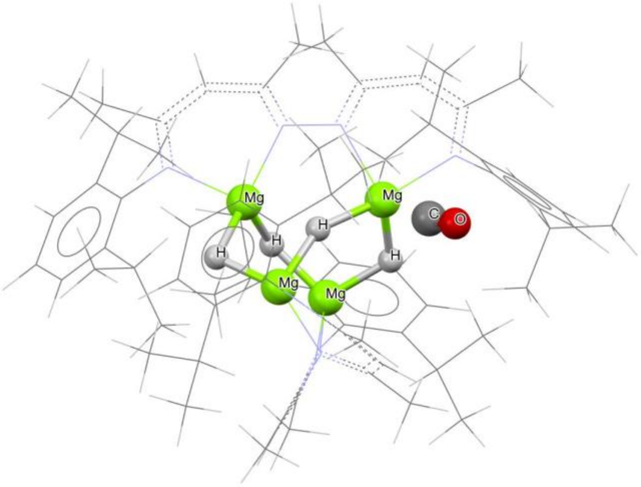

INT-1

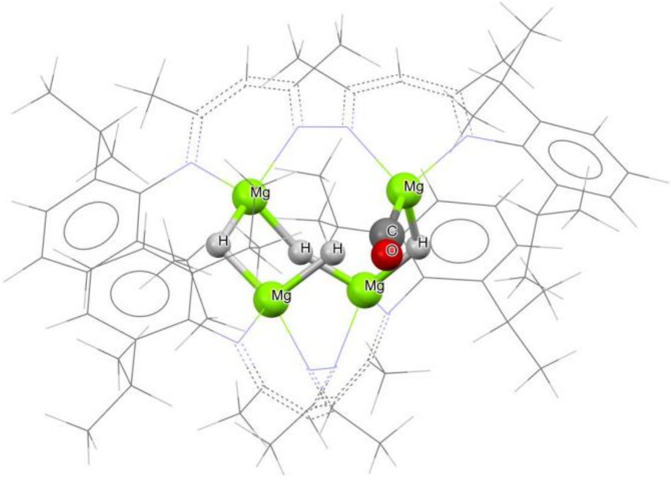

TS1

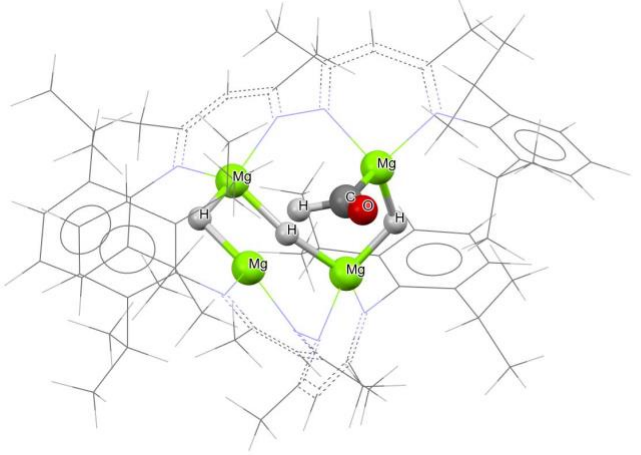

INT-2

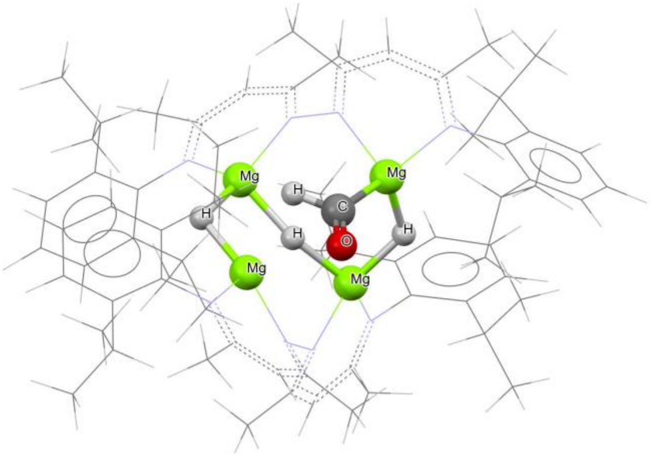

TS2

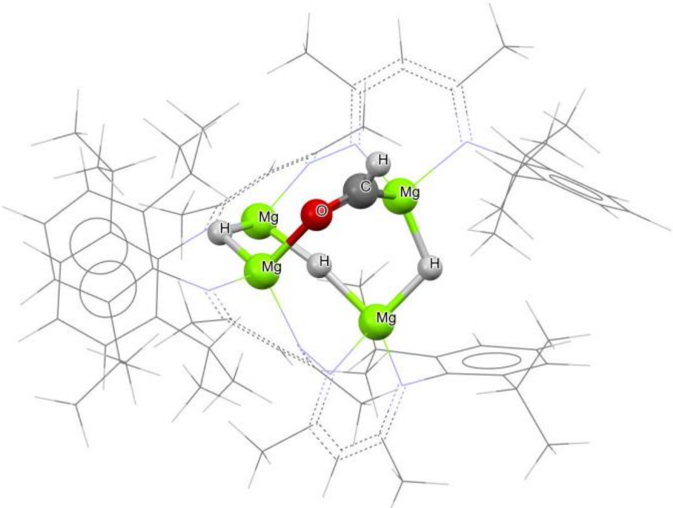

INT-3

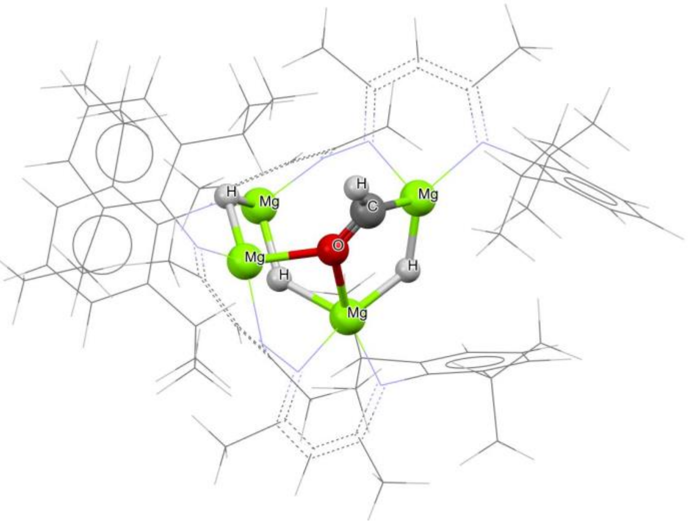

INT-4

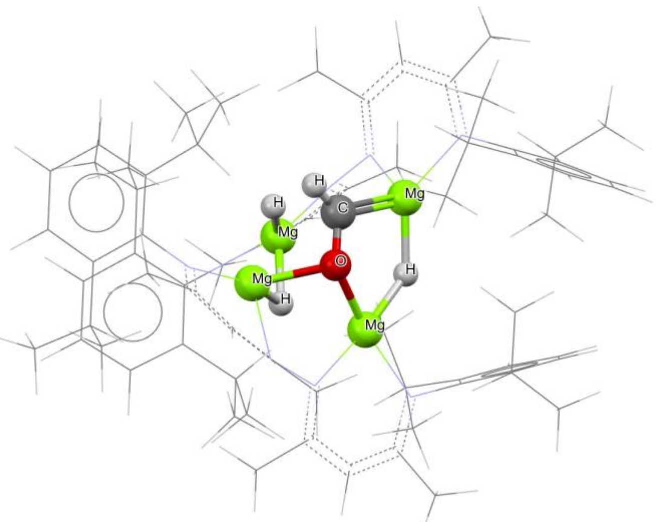

TS3

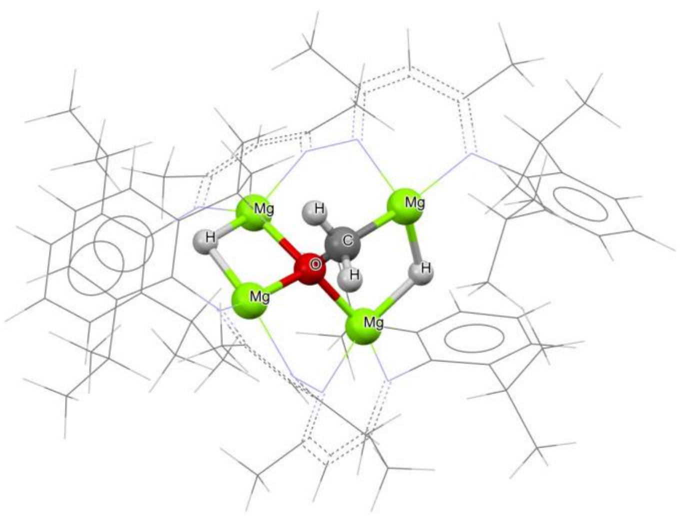

INT-5

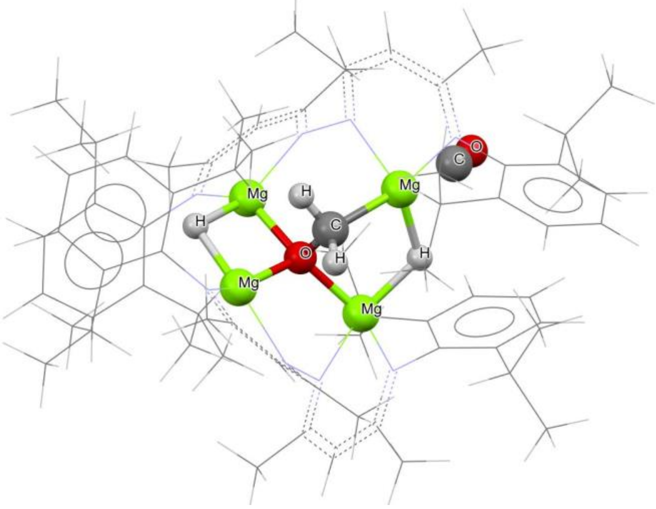

INT-6

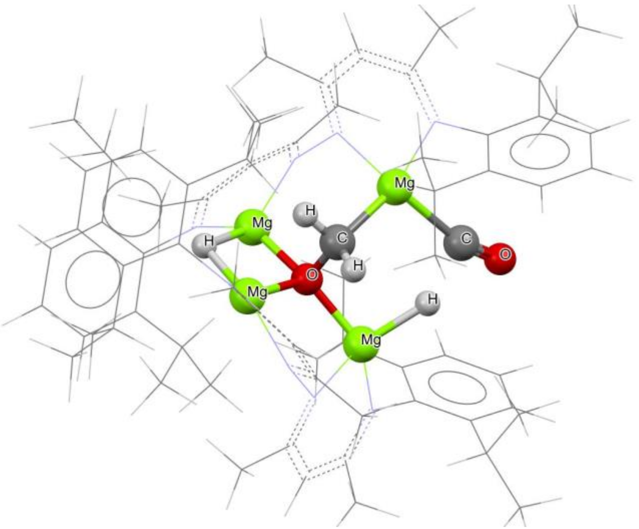

TS4

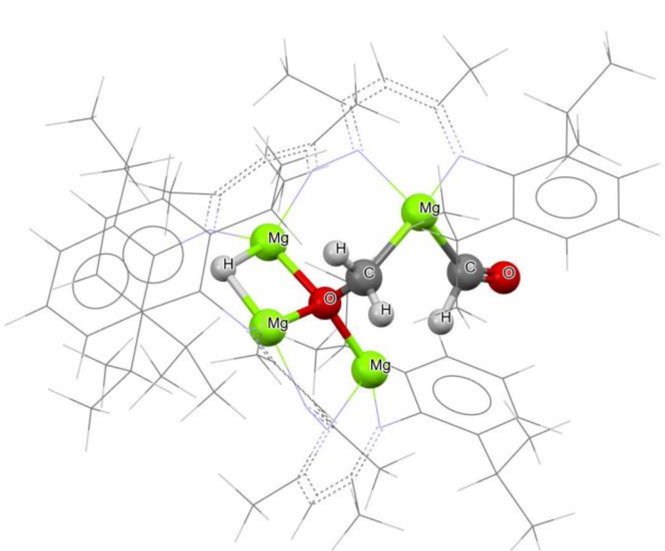

INT-7

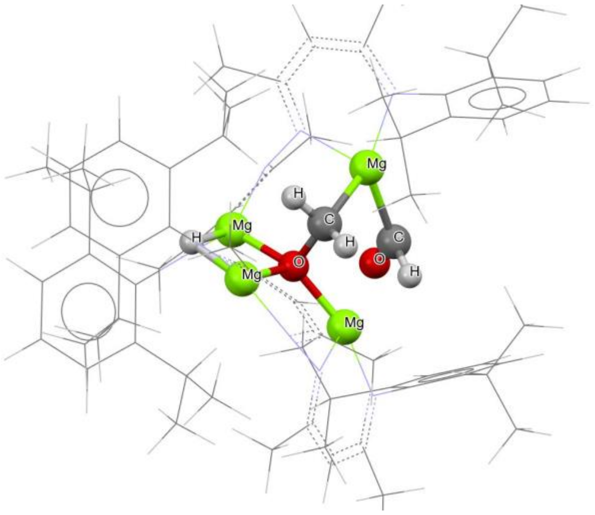

TS5

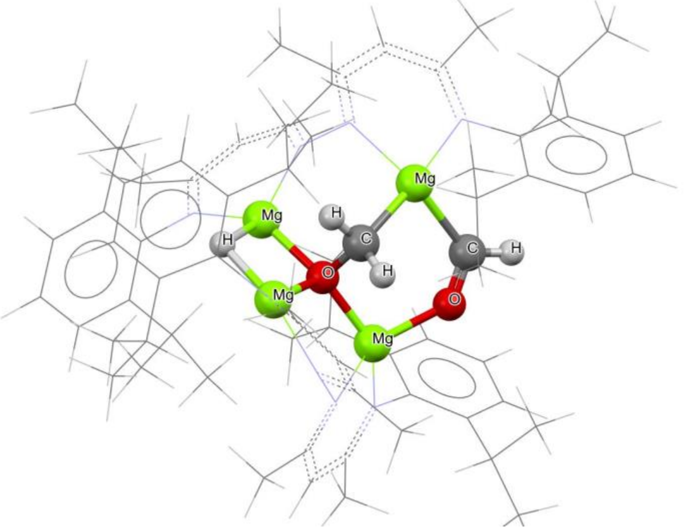

INT-8

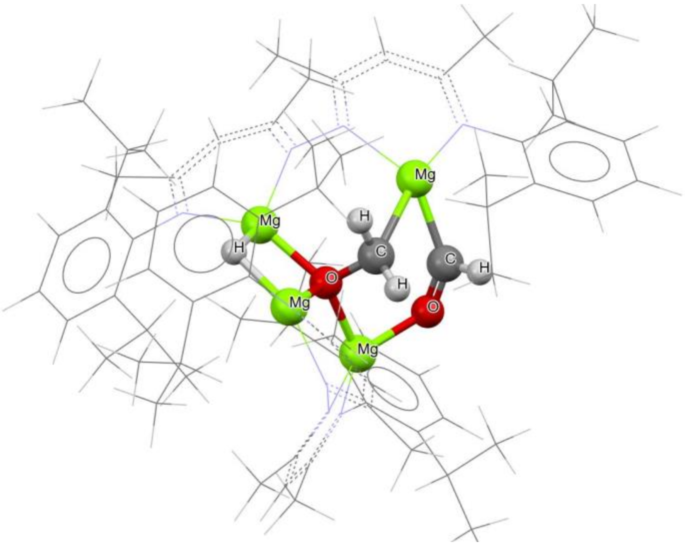

TS6

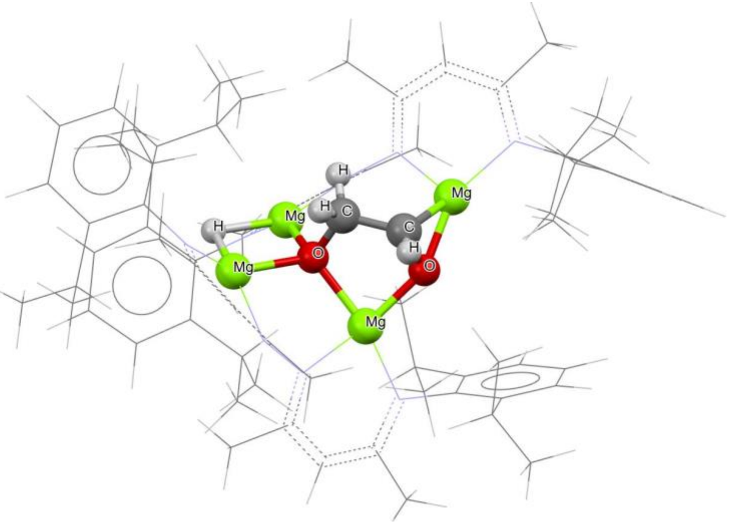

INT-9

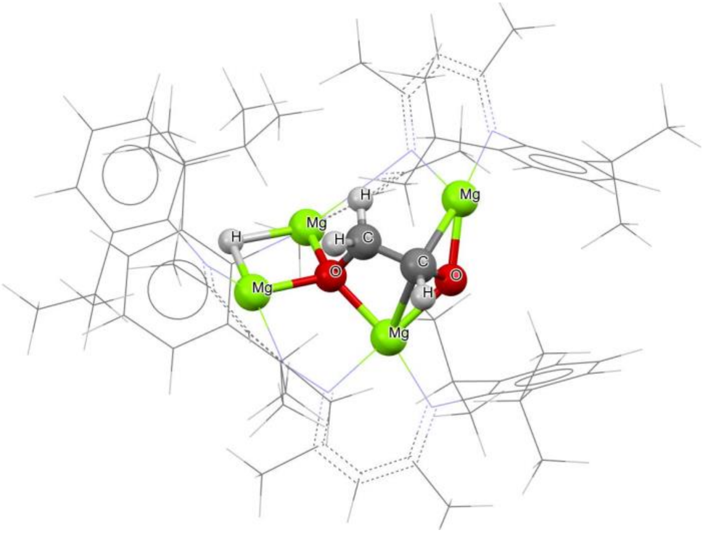

TS7

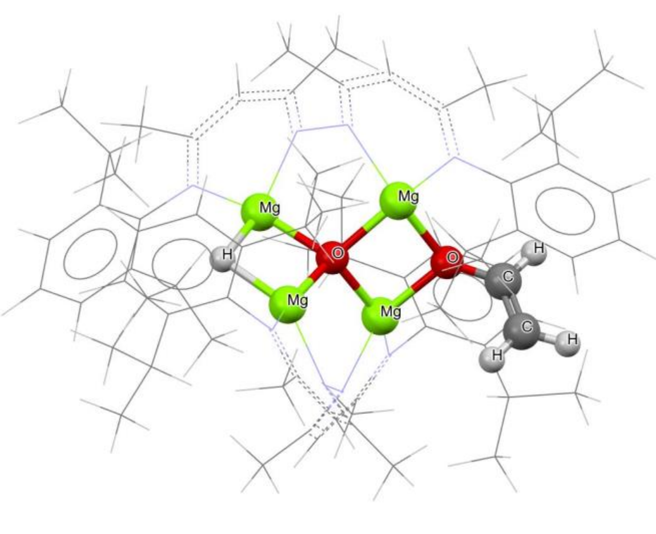

2

4.5. NBO Analysis of Some Reaction Intermediates and TS

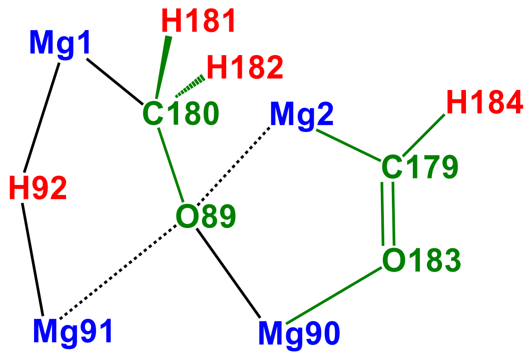

INT-8

Atom	No	Charge
Mg1	1	1.77
Mg2	2	1.67
O89	89	-1.29
Mg90	90	1.83
Mg91	91	1.75
H92	92	-0.79
C179	179	-0.06
C180	180	-0.84
H181	181	0.14
H182	182	0.16
O183	183	-0.83
H184	184	0.08

Table S3. NPA change data for **INT-8**

Wiberg bond index matrix	
Mg1 - C180	0.015
Mg2 - O89	0.005
Mg2 - C179	0.219
Mg90 - O89	0.174
Mg90 - O183	0.215
Mg91 - O89	0.170
C179 - H184	0.971
C179 - O183	1.279
C180 - O89	0.936
C180 - H181	0.968
C180 - H182	0.968

Table S4. WBI data for **INT-8**

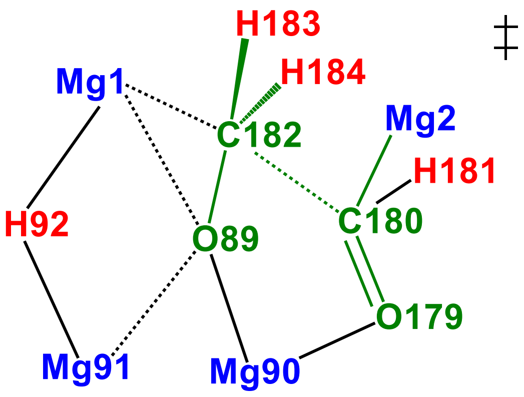

TS6

Atom	No	Charge
Mg1	1	1.77
Mg2	2	1.72
O89	89	-1.26
Mg90	90	1.82
Mg91	91	1.74
H92	92	-0.79
O179	179	-0.99
C180	180	-0.25
H181	181	0.10
C182	182	-0.61
H183	183	0.16
H184	184	0.18

Table S5. NPA change data for **TS6**

Wiberg bond index matrix	
Mg1 - C182	0.010
Mg1 - O89	0.040
Mg2 - C180	0.226
Mg90 - O179	0.284
Mg90 - O89	0.216
Mg91 - O89	0.187
C180 - H181	0.935
C180 - O179	1.307
C180 - C182	0.479
C182 - O89	0.904
C182 - H183	0.917
C182 - H184	0.927

Table S6. WBI data for **TS6**

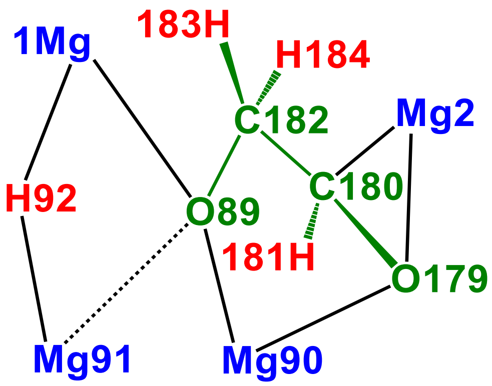

INT-9

Atom	No	Charge
Mg1	1	1.77
Mg2	2	1.80
O89	89	-1.28
Mg90	90	1.85
Mg91	91	1.75
H92	92	-0.79
O179	179	-1.24
C180	180	-0.59
H181	181	0.15
C182	182	-0.11
H183	183	0.15
H184	184	0.17

Table S7. NPA change data for **INT-9**

Wiberg bond index matrix	
Mg1 - C89	0.035
Mg2 - O179	0.050
Mg2 - C180	0.117
Mg90 - O89	0.029
Mg90 - O179	0.034
Mg91- O89	0.032
C180 - H181	0.927
C180 - O179	0.918
C180 - C182	1.057
C182 - O89	0.868
C182 - H183	0.914
C182 - H184	0.919

Table S8. WBI data for **INT-9**

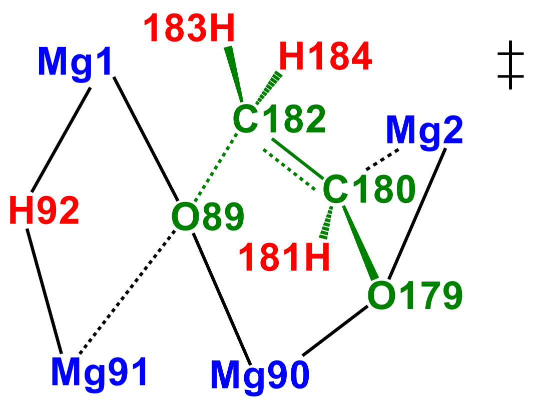

TS7

Atom	No	Charge
Mg1	1	1.76
Mg2	2	1.84
O89	89	-1.47
Mg90	90	1.84
Mg91	91	1.75
H92	92	-0.79
O179	179	-1.18
C180	180	-0.48
H181	181	0.18
C182	182	-0.22
H183	183	0.16
H184	184	0.21

Table S9. NPA change data for **TS7**

Wiberg bond index matrix	
Mg1 - C89	0.040
Mg2 - O179	0.050
Mg2 - C180	0.029
Mg90 - O89	0.035
Mg90 - O179	0.038
Mg91- O89	0.033
C180 - H181	0.905
C180 - O179	0.941
C180 - C182	1.392
C182 - O89	0.558
C182 - H183	0.898
C182 - H184	0.911

Table S10. WBI data for **TS7**

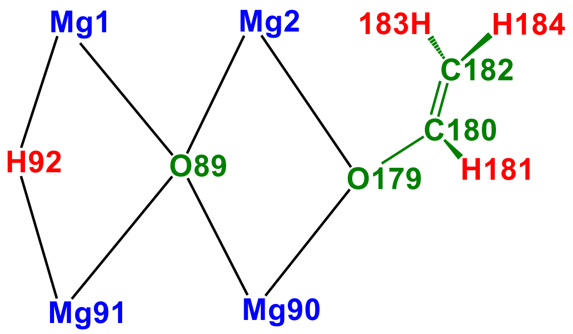

2

Atom	No	Charge
Mg1	1	1.77
Mg2	2	1.86
O89	89	-1.91
Mg90	90	1.86
Mg91	91	1.76
H92	92	-0.78
O179	179	-1.14
C180	180	0.18
H181	181	0.17
C182	182	-0.54
H183	183	0.18
H184	184	0.21

Table S11. NPA change data for **2**

Wiberg bond index matrix	
Mg1 - O89	0.044
Mg2 - O89	0.024
Mg2 - O179	0.031
Mg90 - O89	0.028
Mg90 - O179	0.036
Mg91 - O89	0.044
C180 - H181	0.910
C180 - O179	1.040
C180 - C182	1.880
C182 - H183	0.936
C182 - H184	0.934

Table S12. WBI data for **2**

4.6. QTAIM Analysis of TS7

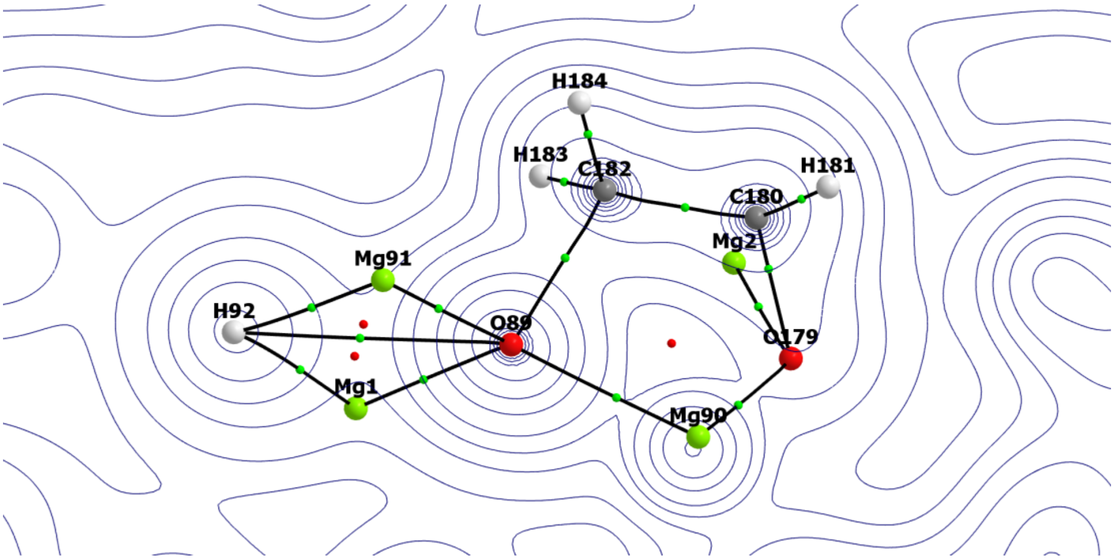

Figure S6. QTAIM molecular graphs for **TS7**

Atoms	r (r) (e bohr^{-3})	∇^2 r (r) (e bohr^{-5})	Ellipticity
C180 - C182	0.289	-0.733	0.319
C180 - H181	0.269	-0.865	0.139
C182 - H183	0.282	-0.963	0.040
C182 - H184	0.278	-0.953	0.032
Mg1 - O89	0.052	0.414	0.055
Mg1 - H92	0.031	0.123	0.128
Mg2 - O179	0.059	0.488	0.015
Mg90 - O179	0.046	0.359	0.017
Mg91 – H92	0.034	0.138	0.094
O179 - C180	0.233	-0.390	0.320
O89 - C182	0.126	0.069	0.083
O89 - Mg90	0.048	0.371	0.036
O89 - Mg91	0.051	0.405	0.049
O89 - H92	0.025	0.039	0.729

Table S13. QTAIM data for **TS7**

4.7 Other Possible Mechanism Pathway

In addition to the reaction pathways shown in Figure S4, Figure S6 considered the reaction pathways resulting from the sequence of CO attacking Mg-H at different position. Figure S7 considered the reaction pathway resulting from the sequence of the second hydride transfer to the formyl ligand.[a]

4.7.1: Possible Mechanism Pathway One：

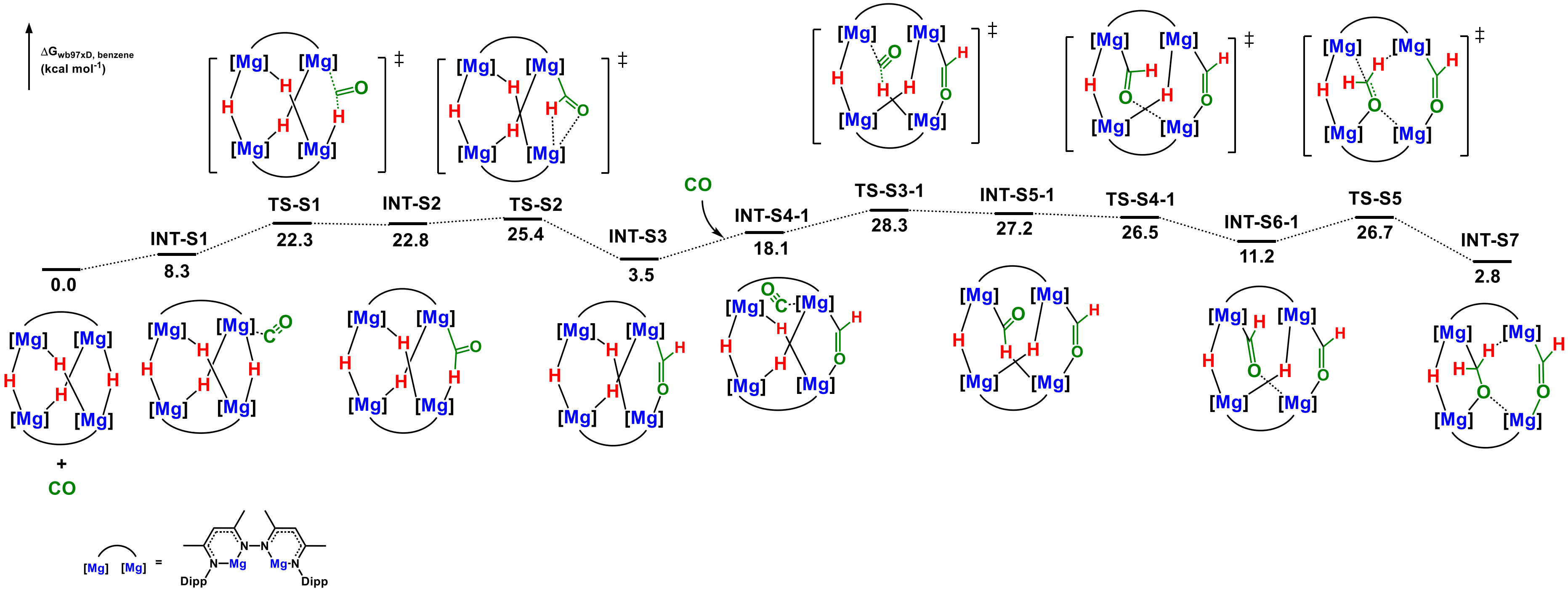

Figure S7. Calculated possible reaction pathway of forming compound **2**. All energies in kcal mol^{-1}

4.7.2: Possible Mechanism Pathway Two :

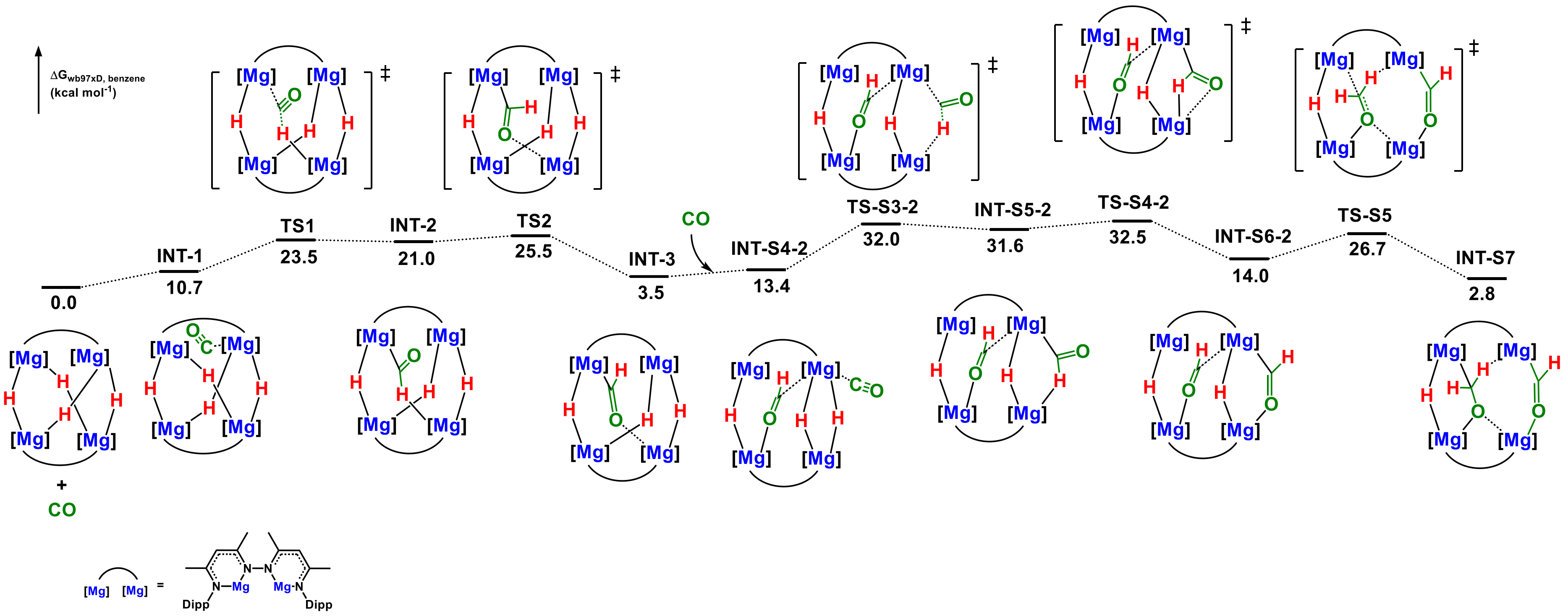

Figure S8. Calculated possible reaction pathway of forming compound **2**. All energies in kcal mol^{-1}

---

[a] Different possible reaction mechanism from INT-S1 to INT-S7 were reported in Figure S7 and Figure S8. The reaction pathway from INT-8 to product 2 is the same as Figure S5.

5) Kinetic Analysis

5.1. Kinetics Experiments

Reactions were monitored in situ in a Bruker 500 MHz machine. The formation of **2** was monitored by quantitative ^{1}H-NMR spectroscopy in the presence of an internal standard (capillary: ferrocene in C6D6, concentration: 0.007 M).

In a N_2 filled glovebox, a 0.1 M stock solution of **1** (40 mg, 0.035 mmol) was prepared in C_6D_6 (2.50 mL), and aliquoted into four J Young NMR tubes with capillaries. (Since the solubility of 1 in C_6D_6 is small at 298K but can be completely dissolved at 333K, $\ln([1]/[1]_0)$ of the test data is selected for analysis and calculation.) The J Young NMR tubes were freeze pumped under vacuum and then filled with CO (~1.5 bar). After that, the NMR tubes were inserted into a preheated NMR spectrometer (323 – 353K).

Four rate constants were determined in the temperature range (323 – 353K) using the initial rates method, by determining the gradient in the linear section of a concentration vs time plot for each temperature (data was plotted to 40 % conversion). A plot of $\ln([1]/[1]_0)$ against 1/T allowed calculation of the thermodynamic parameters using the Eyring equation.

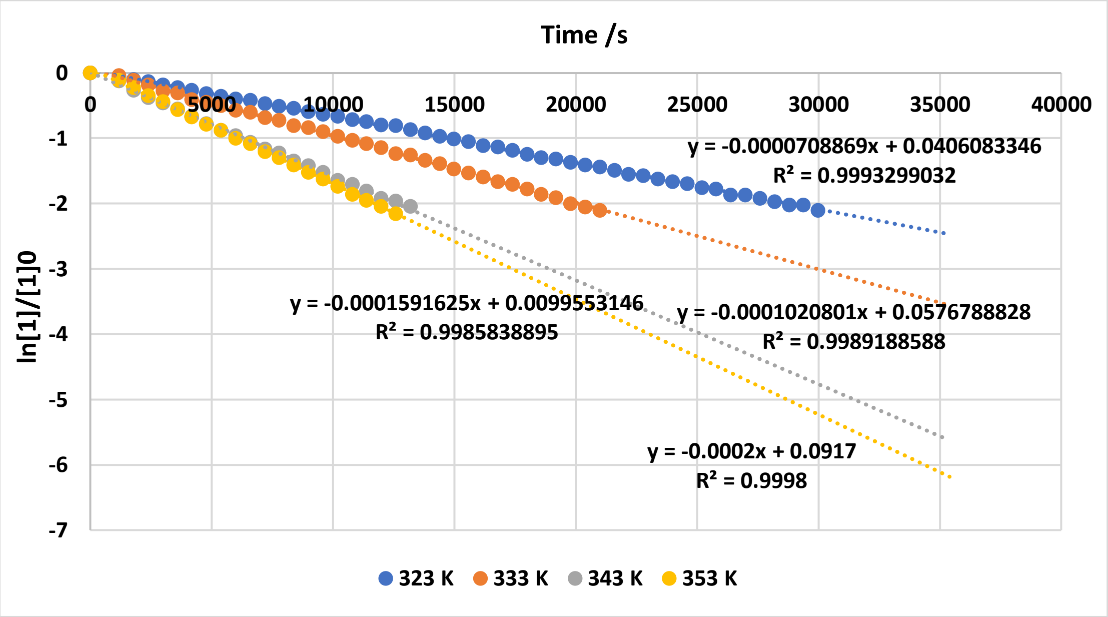

Figure S9. Kinetic data for **1** with CO (323 – 353K).

Temp/K	1/T	k (S^{-1})	Ln(k/T)
323	0.003096	0.0000709000	-15.331892
333	0.003003	0.0001020000	-14.99868
343	0.0029155	0.0001591625	-14.583315
353	0.0028329	0.0002000000	-14.383661

Table S14. Table of constants derived from kinetic experiments

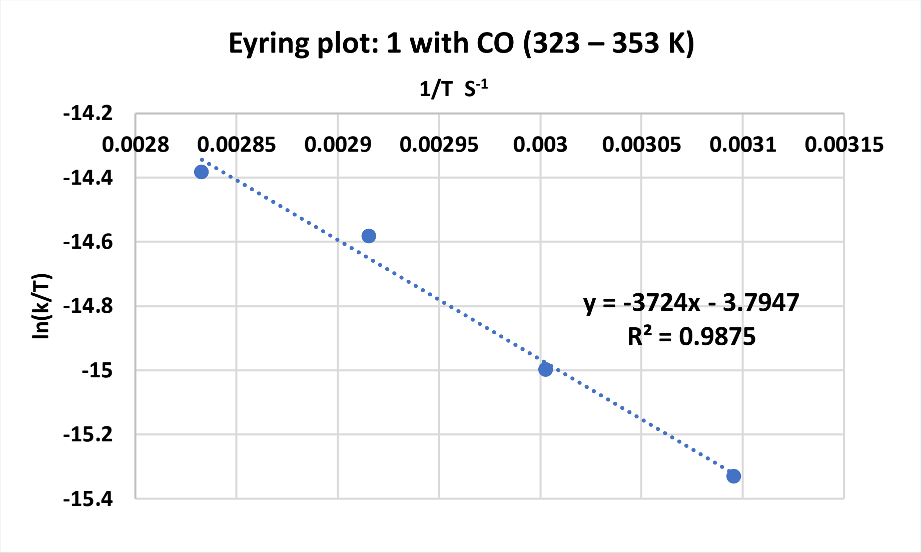

Figure S10. Eyring plot of data from Table S12

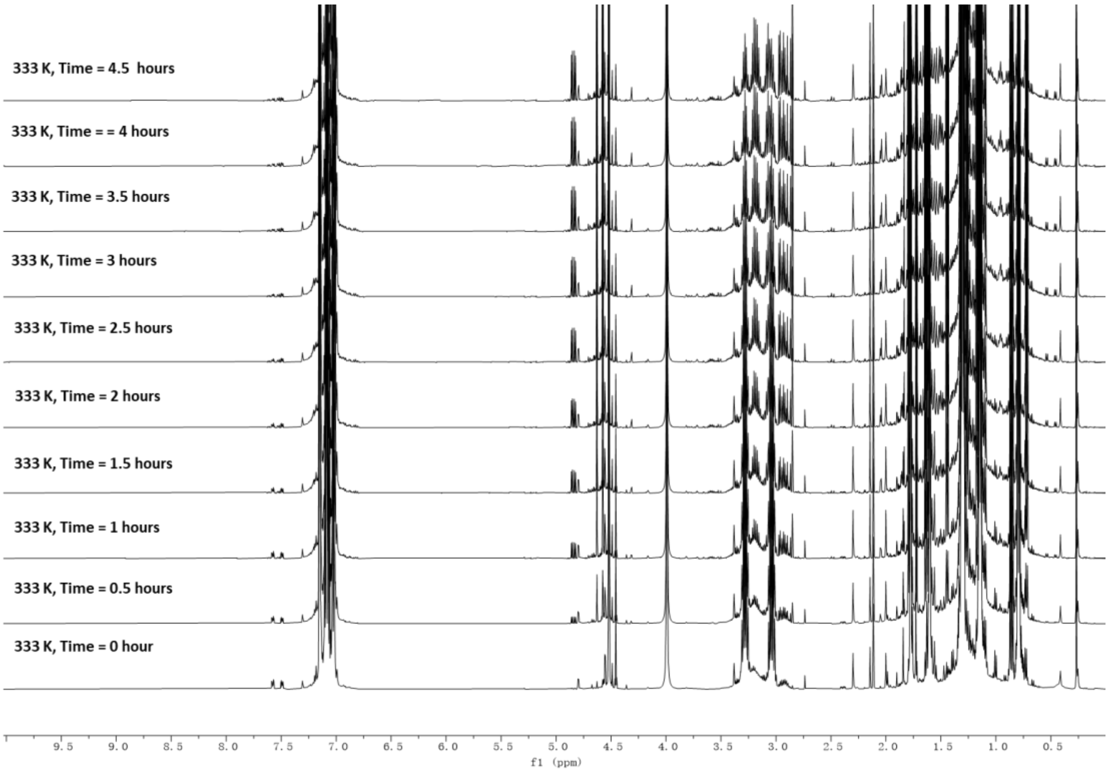

Figure S11. NMR spectrums from kinetic experiments

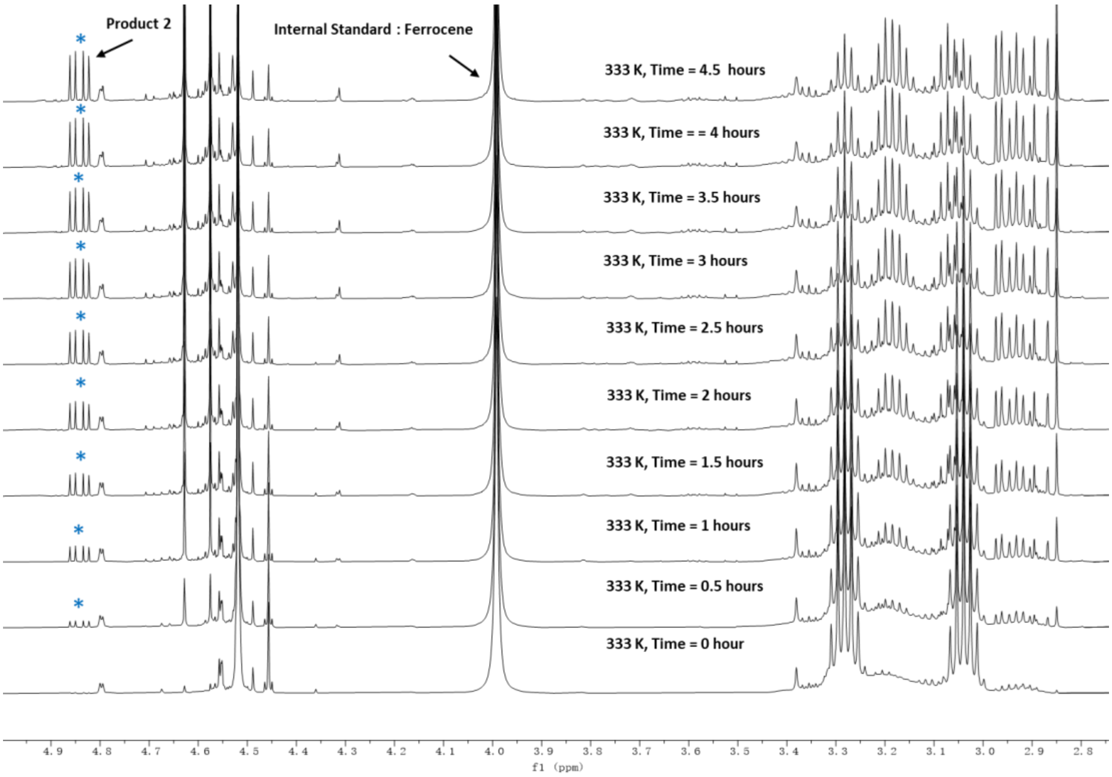

Figure S12. NMR spectrums from kinetic experiments. ("*" is the peak of product **2**.)

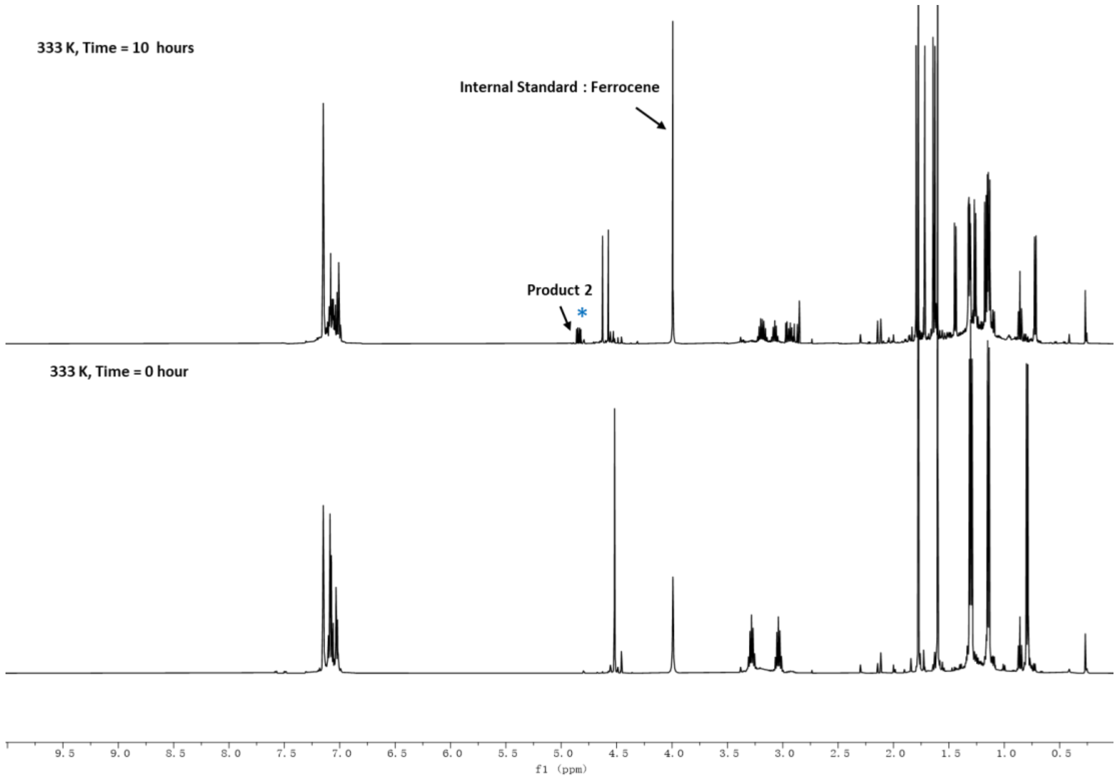

Figure S13. NMR spectrums from kinetic experiments. ("*" is the peak of product **2**.)

6) NMR Spectra

^1H NMR (C_6D_6, 400 MHz) of **2**: ("*"is the solvent peak of Et_2O, "*"is the solvent peak of pentane.)

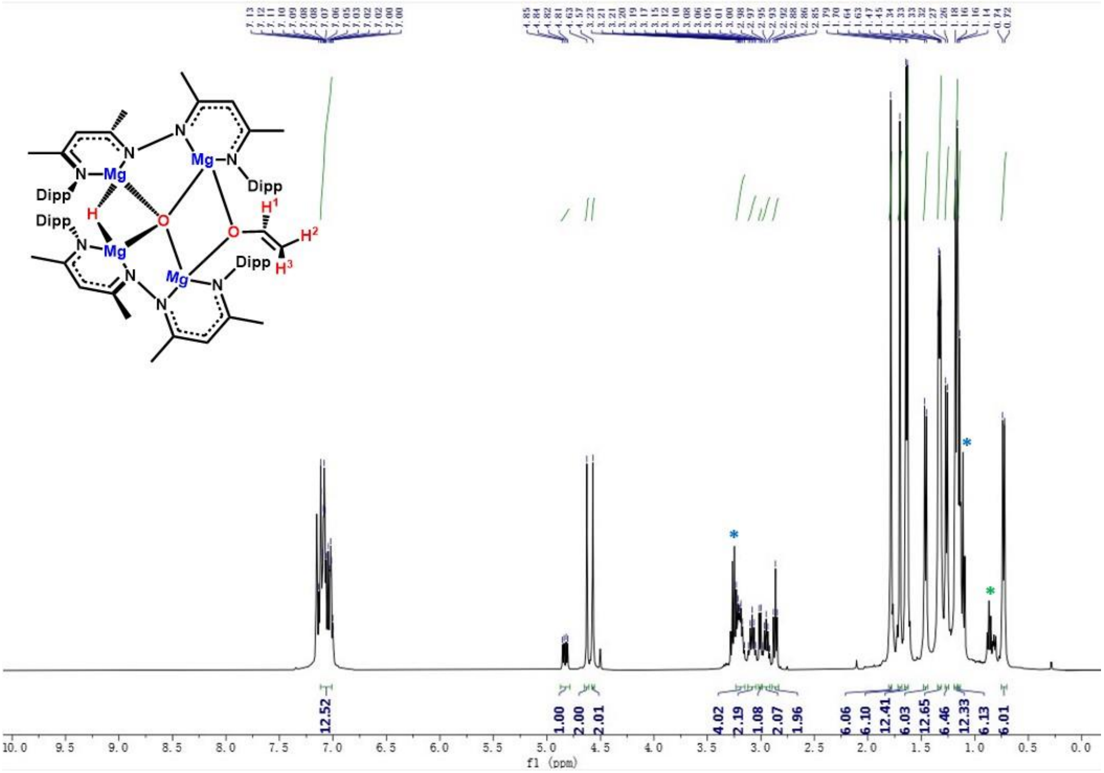

^{13}C NMR (C_6D_6, 101 MHz) of **2**:

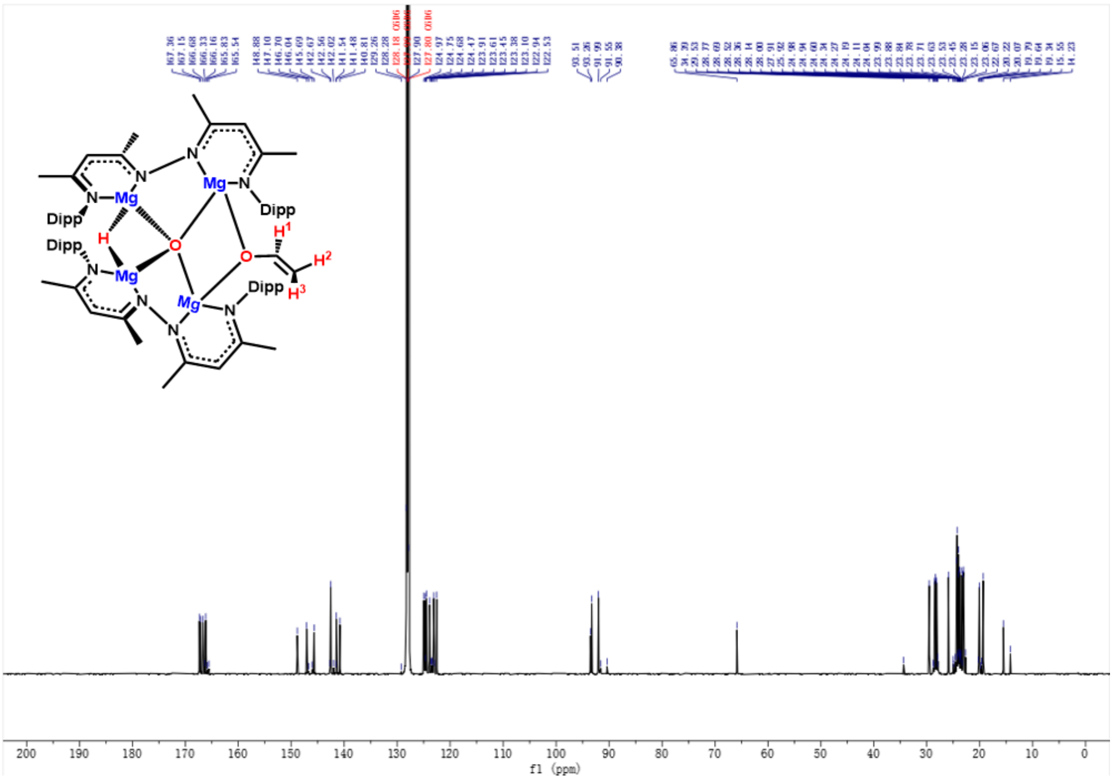

^{1}H NMR (C_6D_6, 400 MHz) of **3a**: ("*"is the solvent peak of Et_2O.)

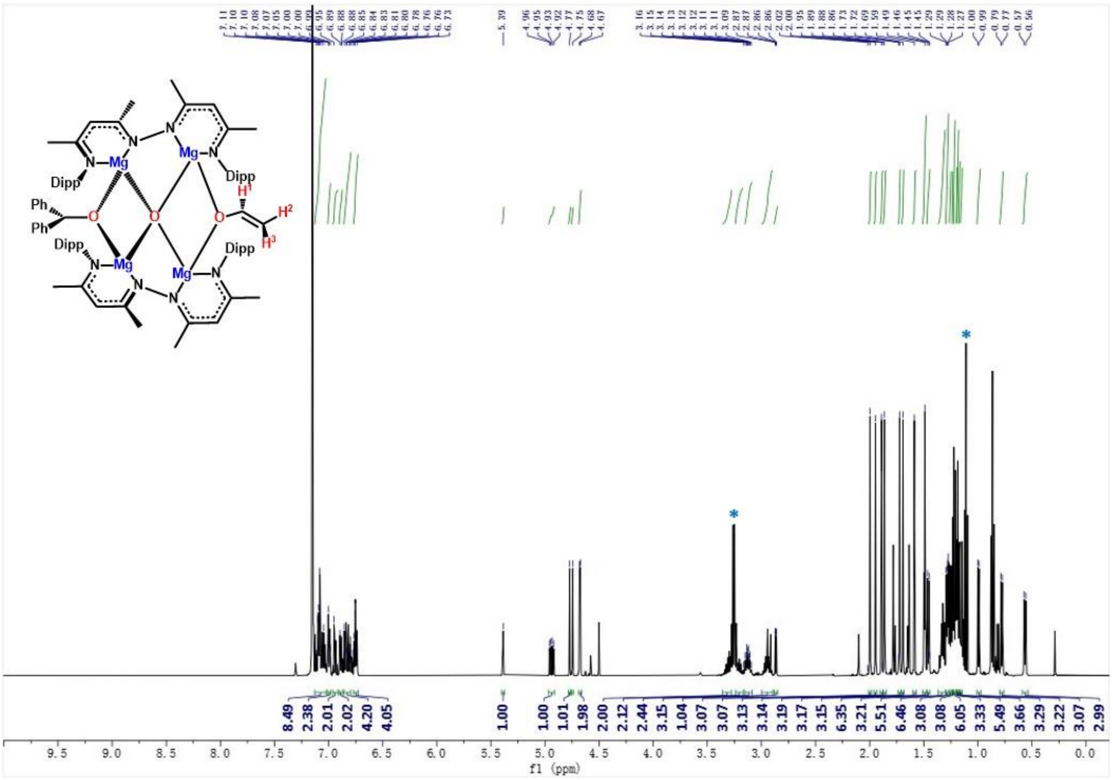

^{13}C NMR (C_6D_6, 101 MHz) of **3a**:

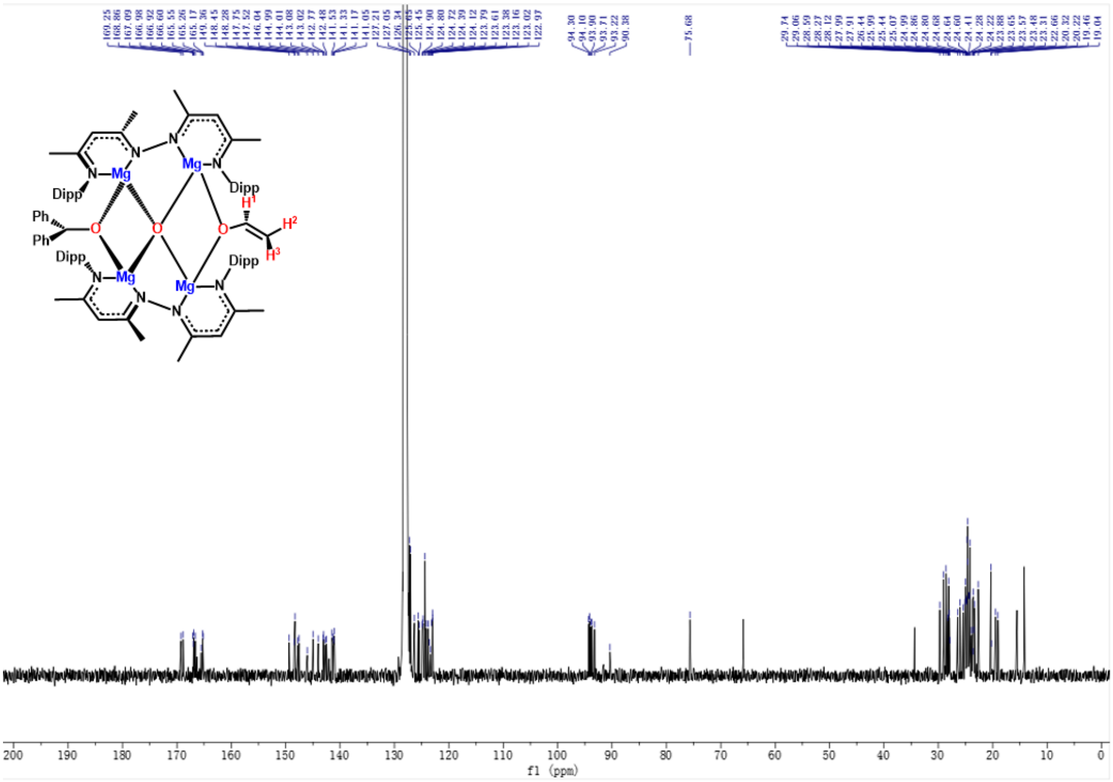

^{1}H NMR (C_6D_6, 400 MHz) of **3b**: (“*”is the solvent peak of Et_2O, “*”is the solvent peak of pentane, “*”is some impurities peaks.)

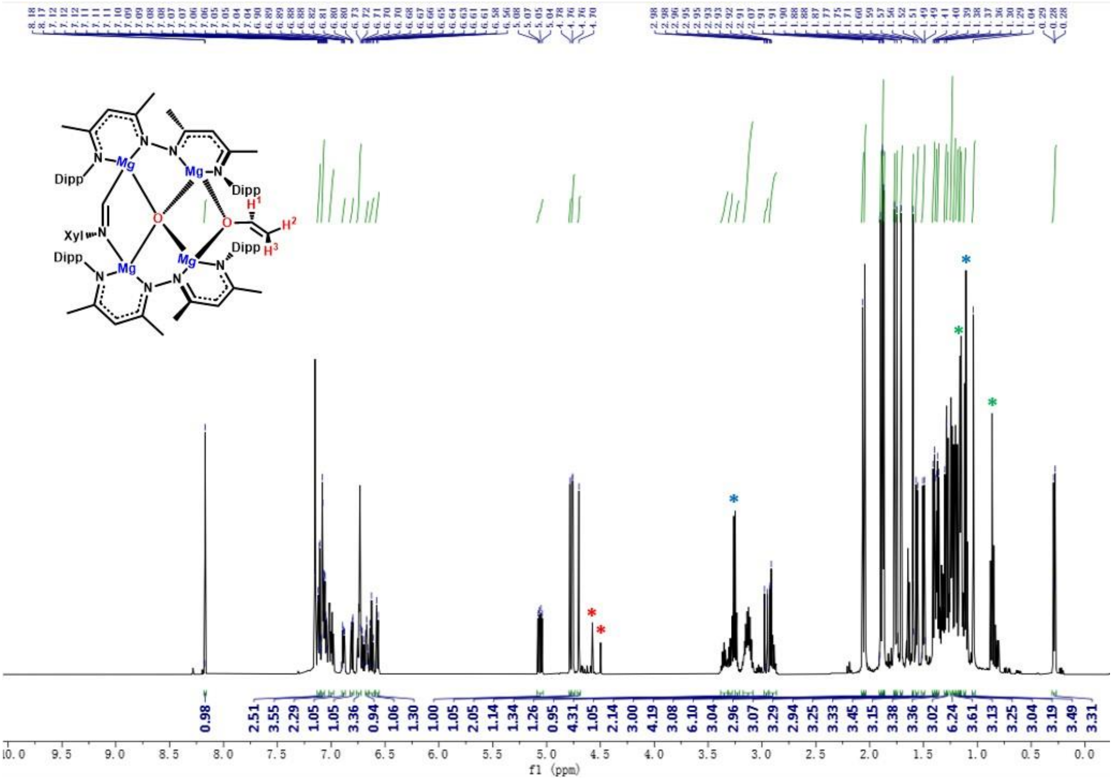

^{13}C NMR (C_6D_6, 101 MHz) of **3b**:

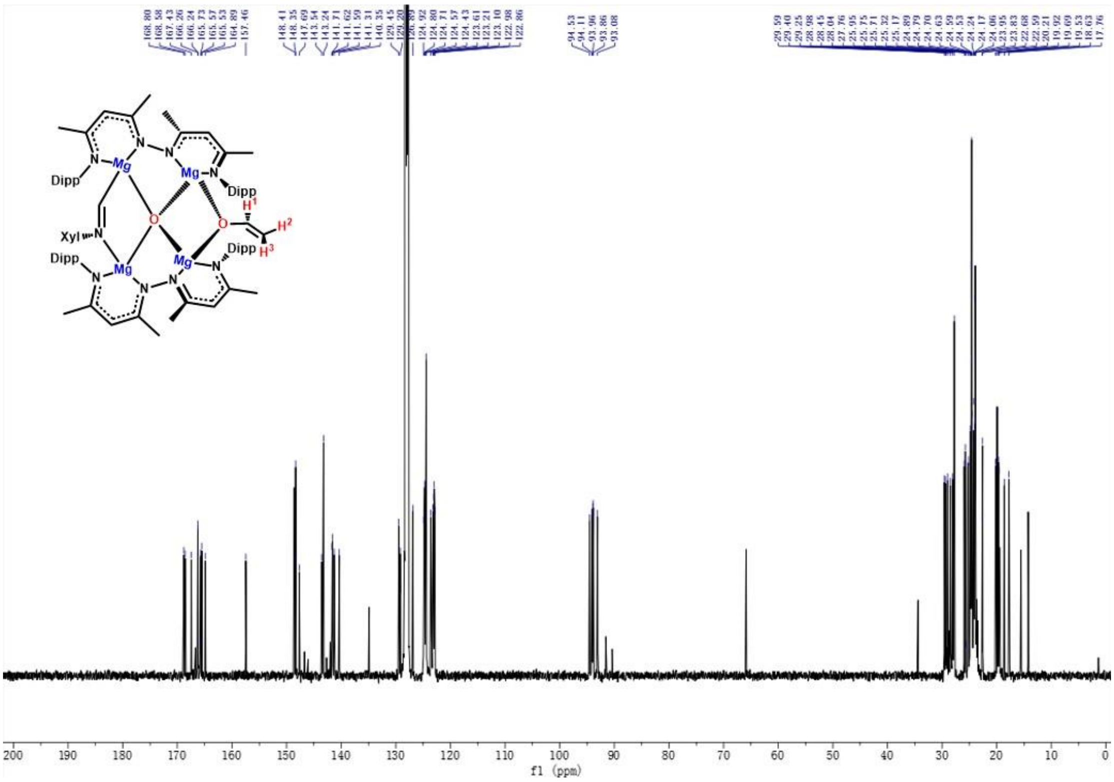

^{1}H NMR (C_6D_6, 400 MHz) of **3c**: ("*"is the impurity peak of PhCN, "*"is the solvent peak of Et_2O.)

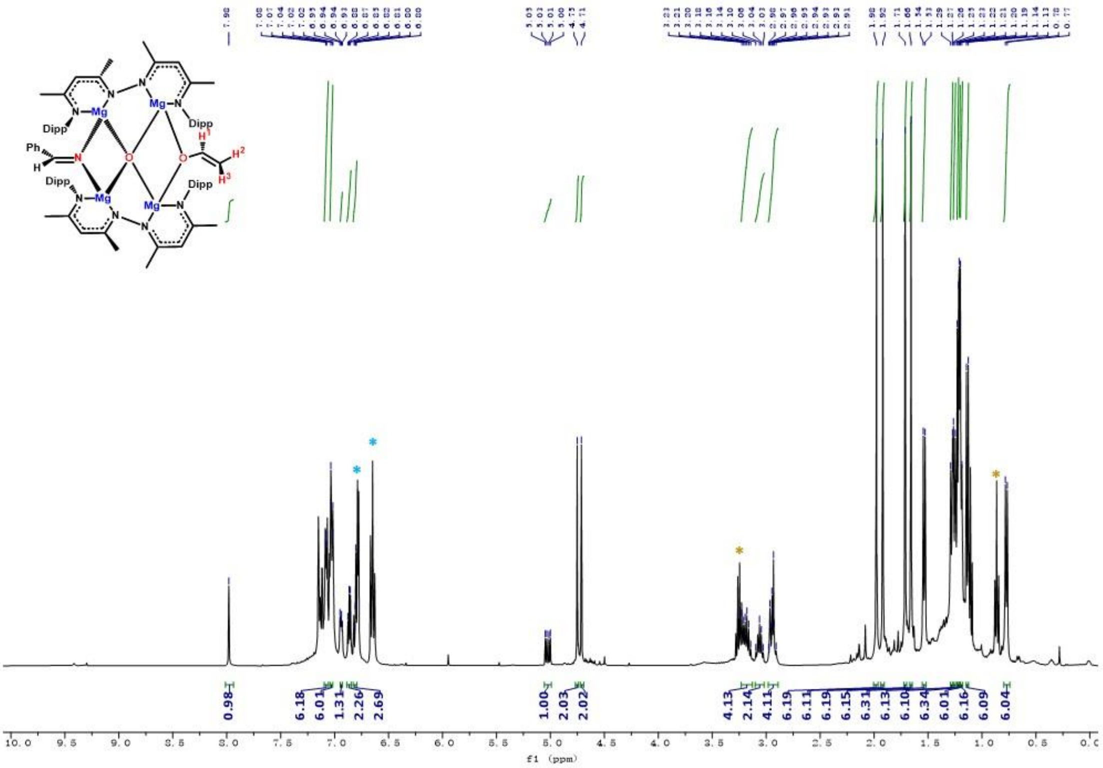

^{13}C NMR (C_6D_6, 101 MHz) of **3c**:

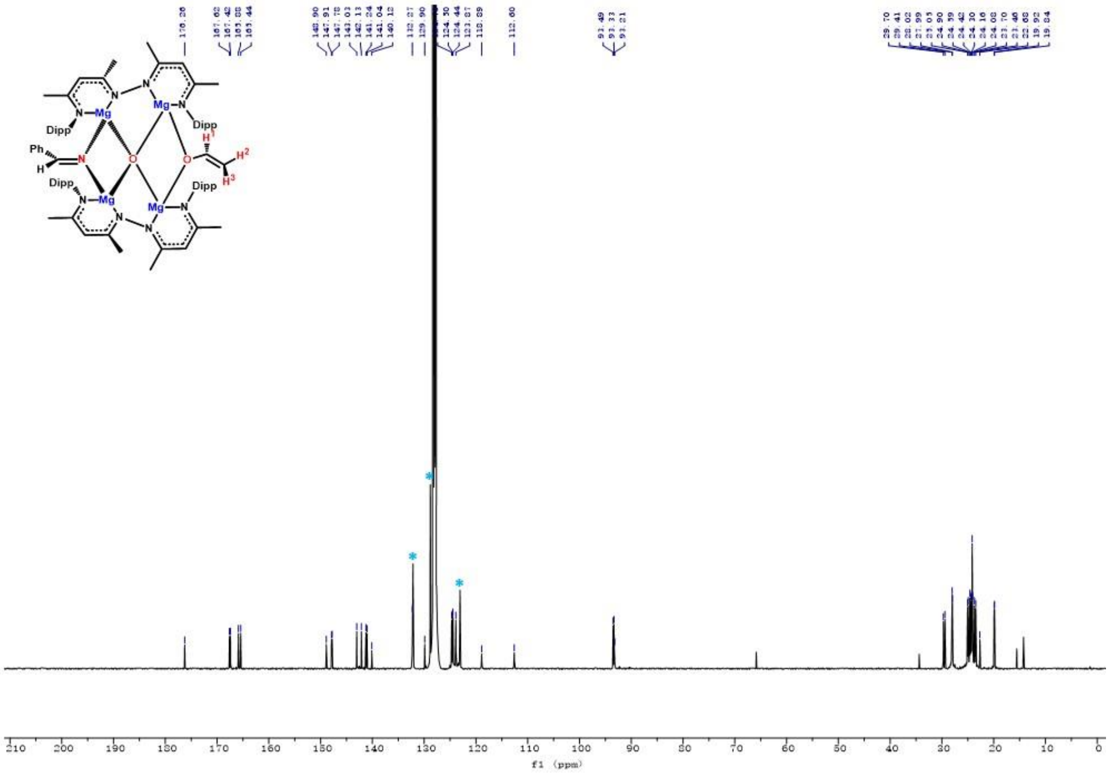

^{1}H NMR (C_6D_6, 400 MHz) of **3d**: ("*"is the impurity peak of ^{t}BuNCO.)

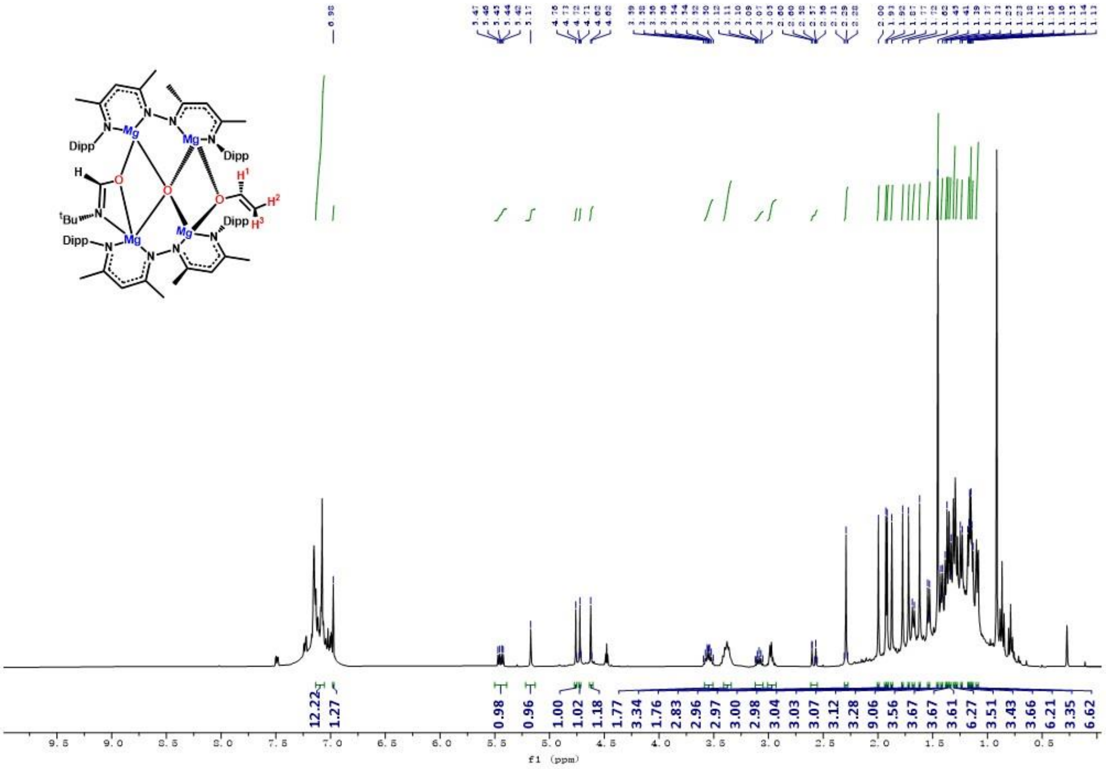

^{13}C NMR (C_6D_6, 101 MHz) of **3d**:

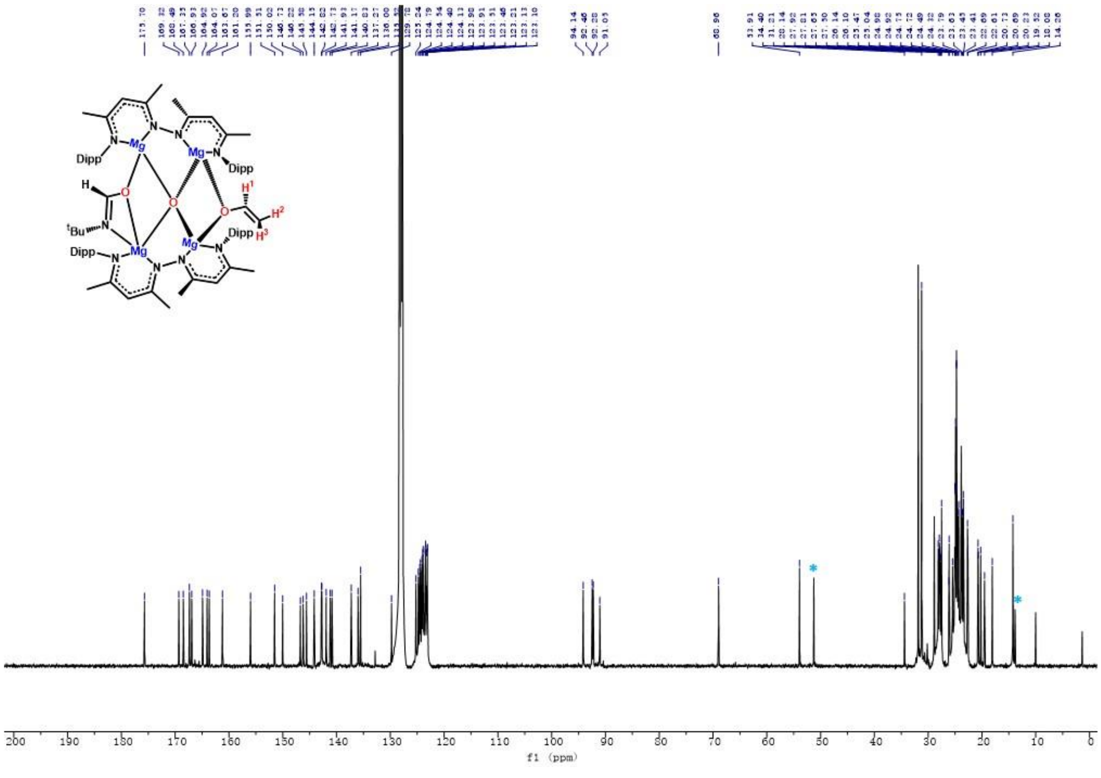

7) IR Spectra

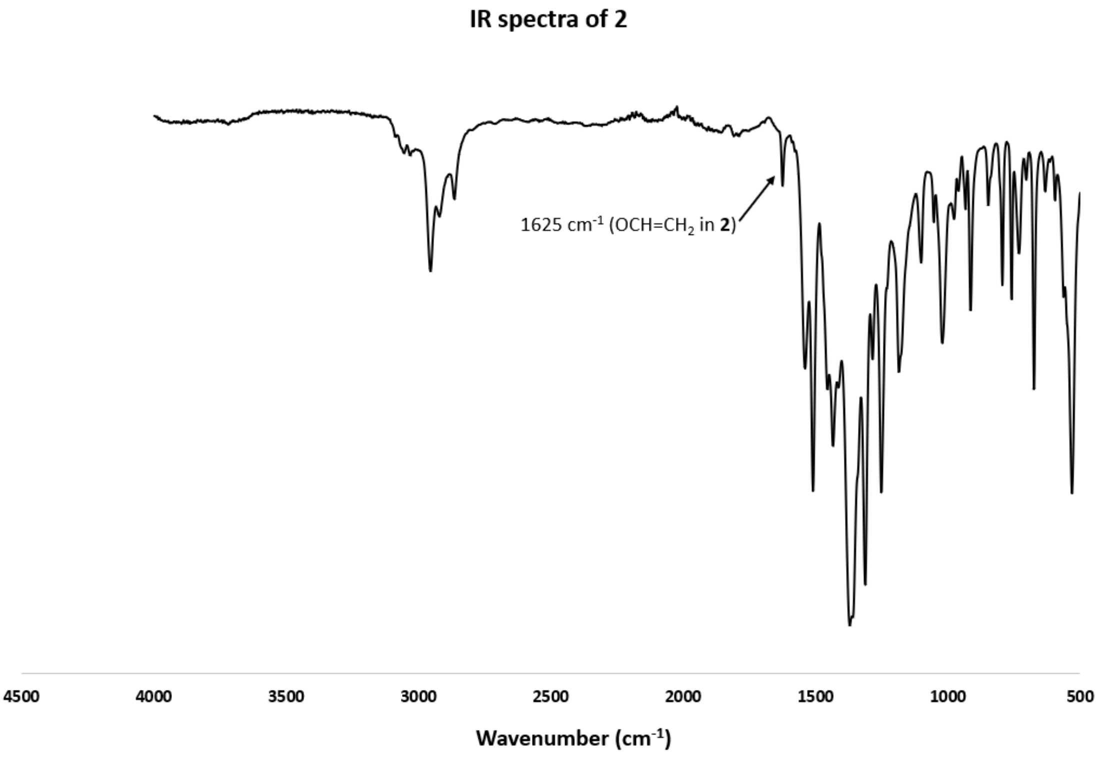

Figure S14. Infrared spectra of **2**

7) XYZ Coordinates

CO.log

SCF (wB97x) = -113.298696921
E(SCF)+ZPE(0 K)= -113.293576
H(298 K)= -113.290271
G(298 K)= -113.312697
Lowest Frequency = 2247.9227cm-1

C	-3.722813	0.240642	0.000000
O	-2.594942	0.240642	0.000000

INT-1.log

SCF (wB97x) = -3205.82525504
E(SCF)+ZPE(0 K)= -3204.262580
H(298 K)= -3204.168064
G(298 K)= -3204.391256
Lowest Frequency = 16.7043cm-1

Mg	8.067610	10.042540	0.565100
Mg	7.976732	6.812895	1.267642
N	8.044712	12.011726	1.064811
C	8.286649	12.435076	2.297584
C	8.353129	11.581924	3.413145
H	8.656993	12.045325	4.345036
C	8.010251	10.210866	3.517831
N	7.559914	9.500361	2.494230
C	8.497998	13.913581	2.539692
H	9.246137	14.315780	1.850431
H	8.811532	14.111365	3.565414
H	7.567385	14.459550	2.349909
C	8.195189	9.601446	4.891995
H	7.404447	9.936083	5.570831
H	9.145746	9.940363	5.310484
H	8.189020	8.512365	4.865640
N	7.260097	8.136084	2.668031
C	6.187475	7.765479	3.339408
C	5.935862	6.400877	3.659284
H	5.047122	6.223831	4.254705
C	6.842049	5.328765	3.546200
N	7.868745	5.342472	2.700961
C	5.190177	8.784708	3.835860
H	5.437069	9.116239	4.848849
H	4.192765	8.341731	3.867272
H	5.179998	9.662914	3.186324
C	6.648072	4.144418	4.466931
H	5.702895	4.212297	5.007240
H	7.462162	4.114210	5.199205
H	6.681065	3.199024	3.917566
C	7.892238	12.922799	-0.016013
C	8.964190	13.137518	-0.902994
C	8.761607	13.960815	-2.012619
H	9.584722	14.139603	-2.699829
C	7.525009	14.539780	-2.262483
H	7.383321	15.173052	-3.133096
C	6.463985	14.289442	-1.399759
H	5.490210	14.719925	-1.615152
C	6.624375	13.482653	-0.273753
C	10.310484	12.454278	-0.710630
H	10.243864	11.822724	0.180183
C	11.439577	13.462417	-0.467469
H	11.238751	14.079791	0.413841
H	12.388089	12.938383	-0.306714
H	11.569558	14.132189	-1.324732
C	10.637442	11.537128	-1.897526
H	9.829228	10.820296	-2.070102
H	10.782177	12.111824	-2.819368
H	11.553950	10.967827	-1.712094
C	5.428763	13.141890	0.603446
H	5.809506	12.747757	1.549724
C	4.598167	12.027726	-0.051762
H	3.764232	11.728043	0.592583
H	4.184164	12.354942	-1.011743
H	5.211513	11.143836	-0.250478
C	4.555593	14.357537	0.932116
H	5.149219	15.168371	1.366170
H	4.049566	14.750180	0.043705
H	3.778735	14.079930	1.651826
C	8.968348	4.454968	2.885753
C	9.013187	3.198856	2.252027
C	10.140669	2.396454	2.431282
H	10.187988	1.425301	1.947183
C	11.214119	2.825767	3.200963
H	12.086027	2.190177	3.323094
C	11.175394	4.081191	3.792768
H	12.027773	4.428214	4.370706

C	10.062282	4.910222	3.651139
C	7.891203	2.749278	1.331979
H	7.015992	3.361772	1.566282
C	7.483554	1.286208	1.533847
H	7.244842	1.081422	2.582094
H	6.600552	1.054058	0.929982
H	8.276793	0.595703	1.229266
C	8.275361	3.015292	-0.132038
H	8.553384	4.063695	-0.286189
H	9.141596	2.407824	-0.415819
H	7.449342	2.769519	-0.808740
C	10.059298	6.292234	4.284693
H	9.065601	6.717503	4.130288
C	11.060911	7.219615	3.584609
H	10.985715	8.239445	3.977767
H	12.092180	6.877136	3.718683
H	10.874671	7.256070	2.506633
C	10.310093	6.244600	5.795822
H	10.220833	7.248362	6.225111
H	9.586051	5.594152	6.296717
H	11.313926	5.873660	6.029707
Mg	10.310398	7.810877	-0.527659
Mg	7.106701	8.711852	-1.811256
H	9.752502	9.316359	0.322468
N	12.266897	7.845243	-1.024280
C	12.729821	8.156484	-2.225834
C	11.906595	8.369723	-3.348170
H	12.408813	8.708016	-4.246171
C	10.519938	8.131326	-3.482762
N	9.765429	7.681567	-2.492105
C	14.222787	8.292044	-2.420540
H	14.725845	7.359106	-2.148604
H	14.622476	9.069739	-1.762779
H	14.474055	8.538740	-3.452403
C	9.888313	8.399557	-4.828986
H	9.500443	7.480243	-5.277871
H	10.607244	8.846584	-5.515966
H	9.034893	9.075104	-4.711318
N	8.390383	7.469974	-2.759252
C	8.005743	6.267758	-3.145725
C	6.648404	5.948123	-3.418936
H	6.480078	4.950902	-3.807385
C	5.511909	6.776921	-3.346172
N	5.510075	7.975513	-2.772574

C	9.038739	5.179527	-3.319301
H	9.729895	5.414821	-4.134410
H	8.565540	4.219363	-3.527237
H	9.642895	5.096049	-2.409860
C	4.229256	6.261432	-3.958162
H	3.432292	6.209487	-3.209878
H	4.363171	5.273726	-4.399797
H	3.888034	6.951797	-4.736466
C	13.127341	7.477824	0.049174
C	13.560634	8.441176	0.982103
C	14.300747	8.012103	2.085635
H	14.642198	8.743967	2.812957
C	14.594923	6.668885	2.280516
H	15.167011	6.356202	3.148843
C	14.142103	5.726683	1.365329
H	14.357312	4.673906	1.528015
C	13.406427	6.108329	0.243423
C	13.232174	9.918924	0.824923
H	12.685211	10.037743	-0.114933
C	14.504036	10.774228	0.742998
H	15.170595	10.432329	-0.054631
H	14.249902	11.821393	0.549015
H	15.066799	10.740724	1.682123
C	12.317314	10.418835	1.952754
H	11.371532	9.870874	1.967041
H	12.798169	10.305540	2.931038
H	12.086081	11.480572	1.811957
C	12.906951	5.051839	-0.731276
H	12.350345	5.564852	-1.520607
C	11.943779	4.073250	-0.048741
H	11.534888	3.366582	-0.780037
H	12.448180	3.492383	0.729497
H	11.107744	4.596491	0.425491
C	14.070059	4.303953	-1.396415
H	14.748398	4.992924	-1.909227
H	14.656252	3.745267	-0.658469
H	13.692219	3.587753	-2.133492
C	4.376043	8.839596	-2.796435
C	3.387630	8.747329	-1.795540
C	2.362236	9.692628	-1.776868
H	1.598795	9.636375	-1.006120
C	2.308680	10.714067	-2.716845
H	1.504510	11.442637	-2.685504
C	3.299526	10.808289	-3.684762

H	3.267782	11.621060	-4.405373
C	4.347011	9.887111	-3.738733
C	3.460645	7.678436	-0.718492
H	4.176618	6.929132	-1.060127
C	2.124580	6.961007	-0.495377
H	1.717849	6.574629	-1.435041
H	2.259665	6.118973	0.191133
H	1.374735	7.625637	-0.053840
C	3.993188	8.258469	0.600316
H	4.996109	8.683977	0.485238
H	3.334859	9.051756	0.970860
H	4.053701	7.480465	1.370167
C	5.453795	10.058548	-4.768532
H	6.120584	9.194764	-4.683445
C	6.282593	11.319530	-4.473295
H	7.117350	11.404389	-5.177797
H	5.670433	12.223127	-4.567998
H	6.691253	11.310420	-3.456543
C	4.910343	10.074375	-6.202372
H	5.734363	10.129198	-6.921086
H	4.331014	9.170560	-6.415127
H	4.260596	10.938537	-6.377673
C	6.074661	5.477555	0.231608
H	7.441118	10.356549	-1.227812
H	9.554764	6.443170	0.426312
O	5.260265	4.778679	-0.104746
H	7.414146	8.128459	-0.023346

INT-2.log

SCF (wB97x) = -3205.81255096
E(SCF)+ZPE(0 K)= -3204.248000
H(298 K)= -3204.154136
G(298 K)= -3204.376150
Lowest Frequency = 14.6137cm-1

Mg	7.546234	9.990642	0.553687
Mg	8.221268	6.464748	1.632757
N	8.010057	11.905394	0.913811
C	8.503827	12.342105	2.069674
C	8.728487	11.520195	3.185152
H	9.225787	11.994030	4.022588
C	8.435165	10.146244	3.364603
N	7.786784	9.417885	2.468178
C	8.892071	13.798204	2.192018
H	9.696222	14.033777	1.486624
H	9.227067	14.040909	3.200671
H	8.048255	14.444133	1.932743
C	8.922236	9.497472	4.638969
H	8.101551	9.287015	5.331374
H	9.642654	10.139864	5.146332
H	9.388169	8.537715	4.403240
N	7.557214	8.053929	2.769287
C	6.546852	7.774918	3.589806
C	6.344421	6.497320	4.153000
H	5.480587	6.405670	4.800220
C	7.217866	5.384223	4.127457
N	8.216456	5.271612	3.269727
C	5.562082	8.861028	3.966221
H	5.999408	9.589857	4.654671
H	4.682092	8.428634	4.443506
H	5.250654	9.414691	3.075398
C	7.000747	4.301853	5.159599
H	6.068823	4.450771	5.705852
H	7.828466	4.306178	5.877303
H	6.991266	3.313799	4.690668
C	7.755446	12.806024	-0.162925
C	8.752905	13.051128	-1.126866
C	8.459161	13.897710	-2.196560
H	9.220069	14.093922	-2.946996
C	7.204966	14.479393	-2.328123
H	6.993886	15.138390	-3.164643
C	6.213385	14.186598	-1.401337
H	5.221326	14.612064	-1.526763
C	6.461780	13.342073	-0.318437
C	10.095387	12.340718	-1.068698
H	10.171016	11.853648	-0.093276
C	11.291320	13.288122	-1.203351
H	11.276473	14.062543	-0.430150
H	12.227043	12.727503	-1.108716
H	11.307231	13.787807	-2.177532
C	10.150279	11.240059	-2.136912
H	9.322963	10.531798	-2.016532
H	10.072343	11.664517	-3.144232
H	11.083895	10.671089	-2.082847
C	5.330720	12.987862	0.634513
H	5.732706	12.298409	1.383566
C	4.197429	12.263990	-0.108078

H	3.429882	11.925580	0.596697
H	3.711553	12.922803	-0.835667
H	4.571064	11.392808	-0.654646
C	4.807610	14.219883	1.383852
H	5.606859	14.713607	1.945476
H	4.381514	14.953943	0.691239
H	4.022329	13.933333	2.090914
C	9.184793	4.235872	3.388831
C	9.057496	3.064315	2.620803
C	10.063673	2.099470	2.704771
H	9.977832	1.189210	2.117380
C	11.177725	2.291101	3.509859
H	11.952118	1.531674	3.560057
C	11.308243	3.469995	4.234875
H	12.196598	3.628328	4.839307
C	10.325027	4.457536	4.187284
C	7.891706	2.864402	1.665837
H	7.187568	3.689151	1.807833
C	7.127530	1.563250	1.936862
H	6.772962	1.519034	2.971491
H	6.258665	1.492028	1.275431
H	7.754488	0.682341	1.760325
C	8.376568	2.924735	0.209455
H	8.917588	3.857132	0.014091
H	9.057643	2.094691	-0.011602
H	7.527744	2.878997	-0.478033
C	10.523656	5.783515	4.905199
H	9.545293	6.265269	4.992249
C	11.419761	6.695624	4.053758
H	11.560290	7.671875	4.531491
H	12.406891	6.245609	3.912203
H	10.995104	6.857318	3.056810
C	11.085483	5.630232	6.322036
H	11.097686	6.601505	6.827044
H	10.478992	4.942215	6.919250
H	12.114365	5.255027	6.316328
Mg	10.065307	7.513446	-0.639860
Mg	6.976892	8.493204	-1.867708
H	8.518388	8.598974	-0.320696
N	12.044841	7.817336	-0.946176
C	12.573849	8.158714	-2.119905
C	11.830399	8.337792	-3.298200
H	12.377623	8.732529	-4.145431
C	10.455195	8.096647	-3.526741
N	9.673706	7.519575	-2.629811
C	14.061999	8.420194	-2.205062
H	14.626947	7.560988	-1.832798
H	14.334695	9.271979	-1.573039
H	14.371692	8.629435	-3.229183
C	9.871914	8.564158	-4.839902
H	9.457624	7.740719	-5.427334
H	10.625153	9.076939	-5.438363
H	9.043332	9.252950	-4.642378
N	8.296160	7.398327	-2.938228
C	7.905765	6.336865	-3.640379
C	6.583823	6.158497	-4.090002
H	6.404938	5.258716	-4.665285
C	5.478480	7.022514	-3.939847
N	5.491068	8.090711	-3.158532
C	8.914458	5.265801	-3.985130
H	9.657614	5.623474	-4.703715
H	8.418486	4.391939	-4.407839
H	9.463513	4.968029	-3.086983
C	4.230260	6.703273	-4.728740
H	3.374519	6.583015	-4.058313
H	4.349189	5.794027	-5.318251
H	3.992170	7.533083	-5.402212
C	12.903957	7.561509	0.165778
C	13.285024	8.609058	1.026787
C	14.117179	8.317748	2.108453
H	14.418228	9.114922	2.781721
C	14.565759	7.024602	2.342915
H	15.221849	6.818616	3.183060
C	14.144684	5.993183	1.515309
H	14.468470	4.976490	1.720370
C	13.299071	6.235046	0.431521
C	12.756290	10.022677	0.837851
H	12.359343	10.102614	-0.179379
C	13.838966	11.096307	0.993626
H	14.686973	10.908317	0.327805
H	13.428226	12.082644	0.756173
H	14.221474	11.144580	2.018280
C	11.596164	10.278346	1.809945
H	10.753769	9.601035	1.626345
H	11.913979	10.125111	2.847210
H	11.221874	11.302772	1.724485
C	12.820236	5.065960	-0.415906
H	12.100471	5.452425	-1.144115

C	12.100023	4.017375	0.443057
H	11.662473	3.237714	-0.189642
H	12.783985	3.527285	1.143517
H	11.296347	4.470415	1.030963
C	13.971178	4.438099	-1.213183
H	14.447041	5.172601	-1.870511
H	14.740513	4.036185	-0.544688
H	13.603194	3.615046	-1.834505
C	4.391547	8.995855	-3.111772
C	3.357354	8.807806	-2.175313
C	2.340363	9.762072	-2.108706
H	1.533559	9.629963	-1.392684
C	2.353133	10.887295	-2.922568
H	1.555287	11.620291	-2.852225
C	3.406476	11.084248	-3.806969
H	3.432298	11.983466	-4.416473
C	4.439910	10.153423	-3.913287
C	3.370589	7.643970	-1.195735
H	4.206359	6.986683	-1.452838
C	2.090045	6.803046	-1.257331
H	1.905859	6.427880	-2.268695
H	2.173182	5.942123	-0.587036
H	1.211888	7.382403	-0.951378
C	3.615530	8.156162	0.232949
H	4.511525	8.784850	0.267746
H	2.771174	8.759449	0.586064
H	3.753699	7.318279	0.924280
C	5.627459	10.426598	-4.824436
H	6.231377	9.515142	-4.862095
C	6.505588	11.542634	-4.236815
H	7.404752	11.693756	-4.844915
H	5.961840	12.492321	-4.199229
H	6.819291	11.323937	-3.209898
C	5.205691	10.756087	-6.260686
H	6.087654	10.869847	-6.899394
H	4.580260	9.962345	-6.680617
H	4.639562	11.692248	-6.312148
C	6.643727	6.151853	0.104209
H	6.453682	9.963140	-0.912633
H	9.861761	6.460633	0.808879
O	5.971926	5.229125	-0.294560
H	6.299352	7.193504	-0.399291

INT-3.log

SCF (wB97x) = -3205.84244242
E(SCF)+ZPE(0 K)= -3204.277086
H(298 K)= -3204.183142
G(298 K)= -3204.404383
Lowest Frequency = 18.0688cm-1

Mg	7.524228	9.871268	0.675363
Mg	8.245242	6.525546	1.793541
N	7.951162	11.830779	0.922201
C	8.451333	12.344537	2.046962
C	8.735475	11.598900	3.200073
H	9.240120	12.135954	3.993861
C	8.494908	10.228688	3.461677
N	7.819499	9.449781	2.634990
C	8.788468	13.819542	2.092628
H	9.594477	14.047972	1.387270
H	9.101484	14.124232	3.091473
H	7.927918	14.423048	1.790671
C	9.075395	9.651362	4.731681
H	8.303454	9.410489	5.468254
H	9.777460	10.351485	5.185614
H	9.591456	8.715639	4.500722
N	7.652800	8.094102	2.998335
C	6.687734	7.811538	3.867465
C	6.524659	6.534007	4.446112
H	5.706834	6.442825	5.150802
C	7.399875	5.427120	4.364805
N	8.340524	5.318837	3.442698
C	5.710339	8.889256	4.284698
H	6.169538	9.620726	4.955820
H	4.854182	8.449625	4.797623
H	5.361342	9.442767	3.408104
C	7.254914	4.344471	5.410161
H	6.362607	4.492928	6.019290
H	8.129813	4.349649	6.069509
H	7.214370	3.355334	4.944815
C	7.674654	12.699918	-0.176838
C	8.689811	13.012795	-1.103089
C	8.393866	13.880447	-2.153906
H	9.167187	14.132997	-2.873540
C	7.121706	14.418885	-2.303950
H	6.910918	15.099856	-3.122929
C	6.114184	14.052748	-1.422658

H	5.110468	14.444409	-1.563835
C	6.365109	13.183639	-0.358891
C	10.058794	12.357977	-1.014265
H	10.165709	11.939229	-0.009928
C	11.220913	13.332250	-1.229775
H	11.169443	14.177481	-0.536322
H	12.174240	12.817438	-1.072465
H	11.234651	13.734053	-2.248223
C	10.138246	11.192875	-2.009548
H	9.356596	10.446568	-1.825091
H	10.006735	11.548783	-3.037660
H	11.103621	10.681579	-1.953231
C	5.226191	12.770404	0.559149
H	5.626821	12.045644	1.274689
C	4.105244	12.082697	-0.232191
H	3.340320	11.689591	0.446593
H	3.611511	12.779254	-0.917874
H	4.492092	11.251736	-0.829634
C	4.692112	13.957515	1.370982
H	5.482840	14.414014	1.974713
H	4.280723	14.731987	0.714285
H	3.893869	13.632495	2.046351
C	9.322129	4.292238	3.510399
C	9.151239	3.102344	2.779171
C	10.175108	2.153460	2.795284
H	10.057017	1.232004	2.231434
C	11.346348	2.374089	3.506838
H	12.135065	1.627897	3.504593
C	11.511296	3.562302	4.208733
H	12.438903	3.739729	4.745140
C	10.512224	4.535244	4.224664
C	7.903495	2.862794	1.944775
H	7.192477	3.660296	2.180047
C	7.220393	1.530574	2.275517
H	6.984586	1.459365	3.341821
H	6.286726	1.432666	1.712348
H	7.854138	0.676064	2.015482
C	8.229573	2.955625	0.446423
H	8.714367	3.907872	0.206170
H	8.913750	2.153742	0.146718
H	7.318698	2.869035	-0.155875
C	10.734511	5.862609	4.933232
H	9.757465	6.335271	5.069724
C	11.578595	6.787233	4.045172

H	11.735181	7.760955	4.523242
H	12.560398	6.347143	3.848039
H	11.102411	6.954108	3.073363
C	11.366281	5.707864	6.320493
H	11.397505	6.677226	6.828362
H	10.793843	5.013970	6.944025
H	12.395679	5.338428	6.262833
Mg	10.006759	7.339871	-0.571039
Mg	6.650336	8.077832	-1.619241
H	8.499606	8.363515	0.016418
N	11.962191	7.721685	-0.935382
C	12.433682	8.124503	-2.115562
C	11.638517	8.345102	-3.249709
H	12.143485	8.790652	-4.098073
C	10.254076	8.111142	-3.430241
N	9.508717	7.490426	-2.531903
C	13.912437	8.416931	-2.253499
H	14.508085	7.557082	-1.934352
H	14.197657	9.253082	-1.606251
H	14.173976	8.665432	-3.282270
C	9.621795	8.641549	-4.696572
H	9.310400	7.842783	-5.375498
H	10.315975	9.293810	-5.227322
H	8.719457	9.204030	-4.440401
N	8.133557	7.331426	-2.820590
C	7.793957	6.347163	-3.648949
C	6.496928	6.207876	-4.183202
H	6.355151	5.367096	-4.851458
C	5.416133	7.112584	-4.086955
N	5.369222	8.092522	-3.199875
C	8.829694	5.324804	-4.064384
H	9.549262	5.735858	-4.778252
H	8.348491	4.462502	-4.527315
H	9.403595	4.994737	-3.193668
C	4.286247	6.954045	-5.078682
H	3.317824	6.957401	-4.570515
H	4.387099	6.034391	-5.655954
H	4.283428	7.801145	-5.773360
C	12.876607	7.463896	0.130602
C	13.272238	8.504638	0.994511
C	14.169174	8.215498	2.022985
H	14.484691	9.006838	2.696478
C	14.661586	6.929570	2.207496
H	15.365720	6.725150	3.008311

C	14.223832	5.902968	1.382723
H	14.583985	4.891359	1.549366
C	13.321062	6.144675	0.345535
C	12.690409	9.903627	0.860634
H	12.257235	9.993064	-0.139910
C	13.736911	11.013224	1.007275
H	14.570437	10.872000	0.312142
H	13.281093	11.987356	0.803232
H	14.148560	11.056019	2.020933
C	11.553111	10.096243	1.873068
H	10.736101	9.385019	1.706863
H	11.911898	9.944155	2.897242
H	11.129526	11.103214	1.813001
C	12.835985	4.985835	-0.511973
H	12.099888	5.381515	-1.218804
C	12.139733	3.917829	0.342799
H	11.714504	3.134235	-0.294212
H	12.838452	3.437197	1.035353
H	11.331206	4.351122	0.938793
C	13.976309	4.380862	-1.341380
H	14.439895	5.132427	-1.988116
H	14.757441	3.965235	-0.695362
H	13.601229	3.571485	-1.976320
C	4.354211	9.088513	-3.264831
C	3.197792	8.971274	-2.472361
C	2.259916	10.005409	-2.502712
H	1.362967	9.928424	-1.893923
C	2.461085	11.135620	-3.282849
H	1.723882	11.932748	-3.289396
C	3.619707	11.249938	-4.041915
H	3.784240	12.146755	-4.632137
C	4.579188	10.237948	-4.048447
C	2.980017	7.773548	-1.561852
H	3.780197	7.053894	-1.755410
C	1.646246	7.067946	-1.835751
H	1.565385	6.762452	-2.883656
H	1.555990	6.172290	-1.212796
H	0.792354	7.716057	-1.610321
C	3.089115	8.191033	-0.087673
H	4.046602	8.684076	0.108127
H	2.290637	8.891974	0.181865
H	3.013120	7.316533	0.566923
C	5.876462	10.406667	-4.824242
H	6.333341	9.418357	-4.929238
C	6.845572	11.285543	-4.022077
H	7.805961	11.392620	-4.539067
H	6.429722	12.286072	-3.872377
H	7.036834	10.869492	-3.027197
C	5.669261	10.966689	-6.235240
H	6.616822	10.961736	-6.783582
H	4.945657	10.369296	-6.798595
H	5.310302	12.001194	-6.216705
C	6.490372	5.942920	0.544089
H	6.296942	9.605335	-0.643378
H	9.803299	6.217897	0.836091
O	6.066271	6.533262	-0.471296
H	5.833637	5.062002	0.787327

INT-4.log

SCF (wB97x) = -3205.83577996
E(SCF)+ZPE(0 K)= -3204.267716
H(298 K)= -3204.175054
G(298 K)= -3204.391955
Lowest Frequency = 15.2661cm-1

Mg	7.675190	10.046308	0.332041
Mg	8.593052	6.510713	1.764506
N	8.053019	11.982363	0.728013
C	8.543037	12.402552	1.889045
C	8.782591	11.566395	2.993540
H	9.265319	12.040846	3.838771
C	8.488462	10.195662	3.170127
N	7.879713	9.448071	2.258834
C	8.865864	13.870595	2.060598
H	9.476626	14.236200	1.230544
H	9.388619	14.056806	2.999054
H	7.942498	14.459616	2.054812
C	8.914627	9.566712	4.477269
H	8.055760	9.299577	5.100415
H	9.552921	10.246234	5.042638
H	9.457988	8.638931	4.278928
N	7.616219	8.102790	2.618453
C	6.526021	7.872575	3.334732
C	6.255646	6.623198	3.942605
H	5.327320	6.571855	4.498575
C	7.109417	5.508614	4.073149
N	8.225867	5.350905	3.376406

C	5.527047	8.985421	3.561210
H	5.911393	9.730413	4.264920
H	4.593383	8.587513	3.960421
H	5.324293	9.510963	2.623496
C	6.728926	4.456625	5.090208
H	5.760787	4.667953	5.544915
H	7.485617	4.418127	5.881301
H	6.699908	3.463728	4.632237
C	7.794324	12.913675	-0.321403
C	8.737142	13.080904	-1.353840
C	8.459007	13.999261	-2.369125
H	9.186426	14.151223	-3.162096
C	7.270036	14.713808	-2.387097
H	7.071439	15.424053	-3.183818
C	6.320291	14.490871	-1.397169
H	5.372411	15.019812	-1.438916
C	6.556929	13.589085	-0.360601
C	10.027918	12.277908	-1.400394
H	10.004503	11.560559	-0.576487
C	11.263304	13.163950	-1.202551
H	11.238439	13.674545	-0.234385
H	12.173977	12.557155	-1.242993
H	11.334874	13.928298	-1.984050
C	10.136280	11.470036	-2.698538
H	9.269818	10.812716	-2.822480
H	10.200234	12.120112	-3.578336
H	11.026930	10.832538	-2.684378
C	5.465757	13.286224	0.654557
H	5.925954	12.751486	1.490452
C	4.421391	12.349986	0.024962
H	3.651551	12.076714	0.755037
H	3.925219	12.833422	-0.823779
H	4.879438	11.428881	-0.350527
C	4.805478	14.546561	1.224041
H	5.548327	15.233449	1.641827
H	4.238191	15.089783	0.460892
H	4.104309	14.277206	2.020227
C	9.117432	4.269541	3.619604
C	8.985501	3.070928	2.891766
C	9.951267	2.077237	3.061822
H	9.866300	1.149702	2.501996
C	11.029446	2.262232	3.916969
H	11.776428	1.482547	4.029225
C	11.153642	3.454239	4.620914
H	12.006201	3.599222	5.278415
C	10.207109	4.470451	4.490165
C	7.849676	2.865988	1.900691
H	7.165726	3.714435	1.998550
C	7.050288	1.588323	2.185673
H	6.670986	1.574622	3.211994
H	6.195600	1.515000	1.505647
H	7.662290	0.691064	2.045195
C	8.377530	2.867028	0.458695
H	8.924540	3.789973	0.238389
H	9.061017	2.027590	0.289467
H	7.551415	2.781768	-0.255386
C	10.380603	5.790858	5.224809
H	9.431184	6.330643	5.162308
C	11.448239	6.646757	4.529738
H	11.559014	7.617369	5.025841
H	12.420678	6.144900	4.549626
H	11.207068	6.828911	3.476462
C	10.705886	5.605321	6.710989
H	10.718036	6.575725	7.217480
H	9.962155	4.971743	7.204133
H	11.689560	5.147451	6.859804
O	7.784191	6.877988	-0.933656
Mg	9.938803	7.670405	-0.957903
Mg	7.143625	8.484992	-2.107574
H	8.850214	9.252891	-0.898832
N	12.000327	7.733712	-1.103540
C	12.668413	8.083559	-2.200286
C	12.067166	8.379265	-3.433661
H	12.730726	8.759295	-4.201060
C	10.706797	8.282368	-3.807954
N	9.790758	7.718778	-3.042310
C	14.175714	8.238504	-2.141389
H	14.638830	7.389031	-1.632871
H	14.445467	9.132974	-1.570912
H	14.600136	8.327590	-3.142047
C	10.314087	8.896063	-5.133478
H	9.975084	8.149994	-5.856740
H	11.152071	9.442382	-5.567472
H	9.478341	9.584847	-4.978959
N	8.451716	7.749553	-3.507699
C	8.049867	6.771159	-4.298524
C	6.709532	6.641918	-4.746125
H	6.548173	5.887323	-5.506529

C	5.570865	7.361467	-4.347728
N	5.555075	8.215275	-3.328585
C	9.026488	5.700932	-4.731475
H	9.860688	6.100843	-5.312248
H	8.525458	4.934063	-5.322849
H	9.462769	5.237764	-3.840580
C	4.295985	7.130470	-5.127701
H	3.470399	6.869609	-4.459518
H	4.417843	6.342288	-5.871318
H	4.005123	8.052861	-5.642211
C	12.730203	7.490782	0.096460
C	13.151404	8.562899	0.905978
C	13.876274	8.282268	2.066451
H	14.212129	9.099626	2.697958
C	14.169577	6.977130	2.432782
H	14.744115	6.778068	3.332446
C	13.687295	5.924536	1.664983
H	13.875098	4.905076	1.986336
C	12.950115	6.158120	0.504378
C	12.771904	10.002816	0.589442
H	12.435910	10.049556	-0.451640
C	13.943003	10.980569	0.743921
H	14.812167	10.665197	0.158231
H	13.648189	11.979672	0.408500
H	14.260148	11.072288	1.787899
C	11.590628	10.431610	1.471124
H	10.712182	9.801162	1.296924
H	11.845905	10.351650	2.533710
H	11.305628	11.469726	1.273358
C	12.388483	4.997393	-0.301611
H	11.469417	5.356144	-0.778711
C	12.005854	3.794785	0.566560
H	11.448700	3.065217	-0.029414
H	12.885395	3.278679	0.966220
H	11.378546	4.088484	1.414489
C	13.340030	4.577852	-1.430288
H	13.511783	5.399224	-2.131121
H	14.307862	4.266560	-1.021423
H	12.922193	3.736504	-1.993656
C	4.374754	8.936747	-2.992263
C	3.522229	8.460888	-1.977650
C	2.420395	9.236212	-1.610653
H	1.752056	8.876973	-0.832297
C	2.169375	10.461595	-2.212874
H	1.309853	11.052645	-1.911771
C	3.035886	10.935484	-3.190479
H	2.853600	11.908039	-3.639017
C	4.145775	10.191957	-3.590443
C	3.790467	7.149586	-1.253980
H	4.696252	6.712874	-1.687505
C	2.654307	6.138961	-1.457159
H	2.479282	5.944399	-2.519631
H	2.897010	5.187454	-0.972555
H	1.716134	6.503850	-1.025177
C	4.052436	7.386813	0.241526
H	4.886416	8.081717	0.380489
H	3.172930	7.815061	0.734604
H	4.293850	6.447502	0.751364
C	5.140612	10.768082	-4.585304
H	5.780375	9.950310	-4.928079
C	6.039632	11.795341	-3.882409
H	6.824455	12.161991	-4.552314
H	5.457443	12.657307	-3.541181
H	6.523104	11.374715	-2.993397
C	4.473127	11.379002	-5.821789
H	5.232985	11.691201	-6.545299
H	3.810450	10.658882	-6.311977
H	3.879651	12.264420	-5.570115
C	7.341357	6.131398	-0.017431
H	6.329333	9.516686	-0.771931
H	6.343324	5.712205	-0.293235
H	10.019970	7.011665	0.791825

INT-5.log

SCF (wB97x) = -3205.89924850
E(SCF)+ZPE(0 K)= -3204.326013
H(298 K)= -3204.233850
G(298 K)= -3204.450488
Lowest Frequency = 13.2082cm-1

Mg	7.497456	9.980730	0.611413
Mg	8.497817	6.731893	1.788753
N	7.991331	11.936164	0.851466
C	8.512259	12.457068	1.961383
C	8.798077	11.717449	3.117926
H	9.320414	12.253291	3.900907
C	8.548986	10.351889	3.387755

N	7.850885	9.568047	2.582344
C	8.878566	13.925675	1.986060
H	9.707574	14.122337	1.297418
H	9.174961	14.243891	2.985875
H	8.039672	14.541292	1.650426
C	9.145771	9.774831	4.650978
H	8.377386	9.447517	5.356970
H	9.785512	10.506230	5.145659
H	9.736806	8.888089	4.400043
N	7.703651	8.215637	2.983096
C	6.684984	7.924637	3.773992
C	6.456088	6.625409	4.289284
H	5.612308	6.536784	4.962796
C	7.247466	5.469765	4.141680
N	8.239162	5.363198	3.268083
C	5.707147	9.008500	4.171769
H	6.183653	9.779497	4.783998
H	4.870731	8.589720	4.732008
H	5.321681	9.512477	3.279745
C	6.945544	4.301695	5.054190
H	6.034914	4.468326	5.630541
H	7.776185	4.153025	5.752824
H	6.844951	3.375351	4.481107
C	7.724985	12.784979	-0.264748
C	8.743947	13.064681	-1.197085
C	8.448977	13.876905	-2.292544
H	9.225261	14.099944	-3.018659
C	7.175945	14.402734	-2.473015
H	6.964471	15.040070	-3.326087
C	6.169653	14.084996	-1.571462
H	5.167246	14.472703	-1.731134
C	6.416705	13.264126	-0.469916
C	10.116784	12.425181	-1.065221
H	10.239561	12.107169	-0.027387
C	11.277413	13.368668	-1.392723
H	11.220132	14.290821	-0.806181
H	12.230114	12.877740	-1.168094
H	11.297227	13.644320	-2.452254
C	10.179506	11.165158	-1.936519
H	9.422660	10.436047	-1.626417
H	9.989645	11.406001	-2.988577
H	11.155068	10.674028	-1.878417
C	5.276677	12.902403	0.468526
H	5.657076	12.157429	1.174621
C	4.100629	12.272245	-0.289654
H	3.341222	11.912654	0.413388
H	3.614216	12.994331	-0.954360
H	4.431046	11.426212	-0.898820
C	4.825176	14.114404	1.294349
H	5.651265	14.523557	1.884421
H	4.447433	14.912406	0.645463
H	4.022262	13.833059	1.983788
C	9.095164	4.227923	3.265819
C	8.872028	3.182223	2.350787
C	9.774145	2.116755	2.318526
H	9.612662	1.302330	1.617087
C	10.881626	2.089013	3.154559
H	11.575220	1.254647	3.114098
C	11.110478	3.145486	4.029049
H	11.994935	3.132862	4.659523
C	10.233013	4.226699	4.098775
C	7.708833	3.222981	1.371908
H	7.105848	4.104786	1.609664
C	6.797979	1.995779	1.495189
H	6.416657	1.886781	2.515147
H	5.941671	2.086792	0.819129
H	7.329089	1.073433	1.236327
C	8.220645	3.388348	-0.067284
H	8.870482	4.265421	-0.152490
H	8.800507	2.513194	-0.381691
H	7.385394	3.514148	-0.764315
C	10.542441	5.421329	4.987956
H	9.623226	6.003444	5.097741
C	11.577857	6.322919	4.298904
H	11.759423	7.231255	4.884649
H	12.532325	5.798992	4.183568
H	11.254262	6.617734	3.294897
C	11.005194	5.024361	6.393514
H	11.113742	5.915857	7.019473
H	10.286186	4.354398	6.875546
H	11.976823	4.519191	6.376833
O	8.205871	8.350528	-0.310814
Mg	10.040705	7.635960	-0.683123
Mg	7.038854	8.701559	-1.947793
H	6.379508	10.089486	-0.894881
N	12.053283	7.891273	-0.983533
C	12.625586	8.239834	-2.133329
C	11.921571	8.461804	-3.327280

H	12.504236	8.847071	-4.155056
C	10.547503	8.267424	-3.593840
N	9.714578	7.723532	-2.723035
C	14.122450	8.464288	-2.176763
H	14.657597	7.590755	-1.794667
H	14.398854	9.306230	-1.533287
H	14.463951	8.672226	-3.191184
C	10.028000	8.741979	-4.932151
H	9.636570	7.919747	-5.537157
H	10.812858	9.246586	-5.496185
H	9.195434	9.435642	-4.776594
N	8.350455	7.637010	-3.109882
C	7.968850	6.547185	-3.754878
C	6.631744	6.321201	-4.158451
H	6.473639	5.433652	-4.758800
C	5.495037	7.118531	-3.933576
N	5.475281	8.166876	-3.117737
C	8.981353	5.470555	-4.073561
H	9.767176	5.831641	-4.742391
H	8.499569	4.608035	-4.534514
H	9.480144	5.150462	-3.153202
C	4.235058	6.746557	-4.683146
H	3.387015	6.646030	-4.000676
H	4.359759	5.816552	-5.238443
H	3.976786	7.542563	-5.389859
C	12.882844	7.603639	0.143236
C	13.267286	8.630394	1.025855
C	14.061163	8.307761	2.127598
H	14.362052	9.091284	2.817262
C	14.471122	7.001942	2.360165
H	15.096925	6.769896	3.216534
C	14.051347	5.991372	1.506001
H	14.345209	4.964801	1.707479
C	13.242054	6.266373	0.403285
C	12.780352	10.058189	0.838057
H	12.394510	10.148796	-0.181179
C	13.889547	11.103350	0.999464
H	14.737998	10.890899	0.341495
H	13.506376	12.099273	0.753608
H	14.264984	11.145143	2.027200
C	11.619580	10.343207	1.799808
H	10.769419	9.675994	1.616218
H	11.928541	10.192383	2.840268
H	11.263800	11.373250	1.705848
C	12.756248	5.120454	-0.470688
H	12.044764	5.532154	-1.193230
C	12.020921	4.059406	0.358890
H	11.565764	3.307112	-0.294520
H	12.698565	3.533901	1.039636
H	11.230230	4.509432	0.966378
C	13.903958	4.498223	-1.277014
H	14.385903	5.240331	-1.921077
H	14.669089	4.080746	-0.613099
H	13.532360	3.687814	-1.913000
C	4.303755	8.972822	-3.009674
C	3.354251	8.716084	-2.002624
C	2.240644	9.552300	-1.903736
H	1.497583	9.359881	-1.134192
C	2.075421	10.635252	-2.756277
H	1.205192	11.277431	-2.661580
C	3.045059	10.908193	-3.713074
H	2.932135	11.777568	-4.354846
C	4.167803	10.092710	-3.854222
C	3.543544	7.594339	-0.993420
H	4.470892	7.072400	-1.248282
C	2.406599	6.566051	-1.037004
H	2.305620	6.123482	-2.032518
H	2.596559	5.756993	-0.324244
H	1.445886	7.022642	-0.775309
C	3.704994	8.168211	0.423293
H	4.488239	8.933406	0.440153
H	2.776679	8.637093	0.768017
H	3.970984	7.379592	1.135778
C	5.260290	10.459796	-4.846616
H	5.938133	9.605228	-4.926224
C	6.071934	11.648432	-4.309558
H	6.922088	11.873200	-4.963150
H	5.449964	12.547525	-4.244619
H	6.452393	11.460886	-3.299179
C	4.720955	10.749028	-6.251598
H	5.548588	10.926055	-6.946006
H	4.131498	9.908591	-6.631064
H	4.084127	11.639901	-6.267787
C	7.381759	7.113588	0.002319
H	6.345432	7.440881	0.197309
H	7.359082	6.483734	-0.902776
H	10.156477	6.808815	0.973377

INT-6.log

SCF (wB97x) = -3319.20153207
E(SCF)+ZPE(0 K)= -3317.621692
H(298 K)= -3317.526125
G(298 K)= -3317.751427
Lowest Frequency = 8.8212cm-1

Mg	7.627063	10.109488	0.595630
Mg	8.812337	6.977957	1.931346
N	8.044511	12.076587	0.752728
C	8.521575	12.660290	1.850479
C	8.740014	11.993301	3.062809
H	9.168004	12.593653	3.857270
C	8.476615	10.647460	3.421267
N	7.945579	9.760189	2.596956
C	8.883626	14.128766	1.800848
H	9.709819	14.287669	1.099552
H	9.182106	14.501122	2.781122
H	8.043141	14.724650	1.434390
C	8.824077	10.280683	4.847737
H	8.083463	10.711139	5.529846
H	9.794193	10.705516	5.115173
H	8.841747	9.202487	5.000462
N	7.802968	8.417544	3.041371
C	6.703336	8.094835	3.697146
C	6.416383	6.776178	4.131506
H	5.527516	6.686218	4.744057
C	7.114746	5.572981	3.907589
N	8.174018	5.453875	3.117690
C	5.677611	9.158569	4.018512
H	6.058285	9.871080	4.755849
H	4.763829	8.711371	4.410260
H	5.434618	9.732696	3.119289
C	6.600165	4.348567	4.635265
H	5.648015	4.544870	5.128972
H	7.327459	4.043396	5.394976
H	6.483331	3.500797	3.956233
C	7.771750	12.854457	-0.411685
C	8.774070	13.048969	-1.381921
C	8.467785	13.777820	-2.532283
H	9.234644	13.932498	-3.286586
C	7.197171	14.301655	-2.731596
H	6.975169	14.872148	-3.628188
C	6.204955	14.066661	-1.788780
H	5.202800	14.449539	-1.962075
C	6.463823	13.331447	-0.631345
C	10.142953	12.408600	-1.230051
H	10.216740	12.032964	-0.207611
C	11.305004	13.382049	-1.447790
H	11.233112	14.247024	-0.781209
H	12.257215	12.877599	-1.252029
H	11.337580	13.754375	-2.477134
C	10.260701	11.201745	-2.168063
H	9.462065	10.478132	-1.972374
H	10.179620	11.505976	-3.217669
H	11.215923	10.681974	-2.044996
C	5.333402	13.042839	0.344248
H	5.732857	12.384044	1.121685
C	4.172906	12.306808	-0.341604
H	3.407289	12.033782	0.392989
H	3.690598	12.934190	-1.099181
H	4.516993	11.392555	-0.833722
C	4.847624	14.322664	1.037498
H	5.662686	14.820150	1.572387
H	4.439852	15.033881	0.310585
H	4.058005	14.092480	1.760359
C	8.744369	4.175655	2.851567
C	8.279037	3.422645	1.750477
C	8.868107	2.184011	1.490843
H	8.505052	1.587373	0.658263
C	9.913691	1.703393	2.267885
H	10.358252	0.737170	2.050051
C	10.399616	2.478355	3.311730
H	11.238930	2.116740	3.900035
C	9.836434	3.718702	3.615100
C	7.180860	3.932238	0.824927
H	6.959515	4.966084	1.103839
C	5.883408	3.125526	0.964814
H	5.483716	3.181304	1.981529
H	5.120070	3.512073	0.280959
H	6.045900	2.069010	0.723963
C	7.653957	3.944383	-0.636322
H	8.601483	4.484077	-0.732503
H	7.805632	2.929964	-1.021077
H	6.918938	4.438449	-1.281302
C	10.444711	4.565273	4.721359
H	9.854259	5.484206	4.793835

C	11.884484	4.958308	4.360049
H	12.317765	5.603245	5.132135
H	12.524441	4.073278	4.270332
H	11.924055	5.490446	3.404428
C	10.394698	3.864752	6.084931
H	10.783806	4.523539	6.868100
H	9.371956	3.583946	6.353807
H	11.001184	2.952462	6.086463
O	8.493181	8.579443	-0.317763
Mg	10.288770	7.801432	-0.615778
Mg	7.252076	8.711937	-1.922214
H	6.470737	10.069037	-0.888324
N	12.286528	8.044471	-0.951058
C	12.855766	8.311242	-2.122861
C	12.145123	8.435839	-3.326411
H	12.720625	8.761549	-4.184353
C	10.770211	8.204431	-3.571432
N	9.947743	7.718163	-2.659613
C	14.350345	8.542093	-2.179876
H	14.888477	7.705936	-1.724928
H	14.617311	9.435734	-1.605733
H	14.696342	8.669177	-3.206102
C	10.240307	8.573661	-4.938412
H	9.825352	7.711523	-5.466886
H	11.024318	9.016477	-5.553249
H	9.422876	9.293757	-4.828991
N	8.580875	7.582548	-3.027812
C	8.216791	6.429782	-3.564926
C	6.878133	6.121734	-3.905459
H	6.743792	5.188502	-4.438457
C	5.704446	6.857198	-3.663397
N	5.654414	7.955598	-2.917420
C	9.254432	5.360274	-3.821294
H	9.993772	5.676563	-4.562379
H	8.784679	4.439044	-4.166473
H	9.807844	5.153910	-2.900013
C	4.433877	6.345958	-4.306564
H	3.634798	6.231947	-3.569976
H	4.595475	5.392145	-4.809460
H	4.077458	7.073181	-5.044192
C	13.090621	7.835357	0.207547
C	13.433157	8.918085	1.039454
C	14.160232	8.665167	2.204391
H	14.428083	9.491009	2.857691
C	14.533205	7.374131	2.554168
H	15.098798	7.196295	3.463643
C	14.159654	6.310537	1.742447
H	14.430771	5.298371	2.030572
C	13.426700	6.515420	0.573478
C	12.973029	10.335904	0.732169
H	12.590438	10.348308	-0.292822
C	14.110987	11.360191	0.810988
H	14.949253	11.075582	0.167615
H	13.756215	12.346633	0.495043
H	14.493604	11.464106	1.831598
C	11.817739	10.734506	1.662651
H	10.956889	10.064425	1.549191
H	12.130497	10.693652	2.711650
H	11.479511	11.755720	1.461154
C	12.997851	5.317567	-0.262776
H	12.338766	5.686245	-1.055173
C	12.204069	4.294050	0.561972
H	11.842266	3.483153	-0.079105
H	12.817362	3.840059	1.347677
H	11.339199	4.758215	1.044794
C	14.202314	4.656358	-0.946843
H	14.737638	5.366360	-1.584748
H	14.911062	4.269909	-0.205979
H	13.877705	3.817369	-1.571186
C	4.412249	8.629041	-2.719075
C	3.596725	8.304514	-1.616627
C	2.406382	9.011088	-1.434702
H	1.764580	8.762896	-0.593300
C	2.035189	10.033941	-2.297026
H	1.107342	10.574370	-2.136760
C	2.871842	10.377203	-3.351174
H	2.597934	11.201554	-4.003846
C	4.065212	9.691073	-3.576769
C	4.003765	7.251277	-0.595984
H	4.986911	6.870732	-0.887648
C	3.040359	6.058304	-0.561905
H	2.990007	5.554105	-1.531484
H	3.369145	5.325938	0.183131
H	2.025942	6.374163	-0.294904
C	4.136869	7.874517	0.803324
H	4.751292	8.780417	0.764904
H	3.158359	8.158632	1.206139
H	4.599677	7.172046	1.506085

C	5.007749	10.145413	-4.679709
H	5.780993	9.379630	-4.789804
C	5.700941	11.451827	-4.262800
H	6.452299	11.753699	-5.000750
H	4.974058	12.266219	-4.168920
H	6.191388	11.357483	-3.287797
C	4.313234	10.294742	-6.037542
H	5.045251	10.543645	-6.812606
H	3.809428	9.368008	-6.329445
H	3.564447	11.093889	-6.025529
C	11.154396	8.110196	3.987081
C	7.734801	7.339493	0.105573
H	6.700517	7.641353	0.342427
H	7.672312	6.665322	-0.763401
O	11.883999	8.521673	4.743048
H	10.448489	7.056022	1.085267

INT-7.log

SCF (wB97x) = -3319.18904041

E(SCF)+ZPE(0 K)= -3317.609062

H(298 K)= -3317.514241

G(298 K)= -3317.735457

Lowest Frequency = 17.8941cm-1

Mg	8.067898	9.988386	0.611423
Mg	8.746793	6.858476	2.336015
N	8.366088	11.984080	0.800693
C	8.780037	12.584495	1.914384
C	9.005852	11.909674	3.123353
H	9.455842	12.493454	3.916952
C	8.666384	10.579720	3.468454
N	8.089047	9.737243	2.627323
C	9.054427	14.071835	1.887797
H	9.832240	14.299026	1.150524
H	9.375859	14.436675	2.863687
H	8.161591	14.624278	1.581357
C	8.974419	10.126566	4.876191
H	8.060285	9.953266	5.451435
H	9.576930	10.869524	5.399463
H	9.513460	9.174903	4.850945
N	7.710505	8.466368	3.135388
C	6.499379	8.378065	3.657931
C	5.942376	7.164372	4.120560
H	4.972998	7.254876	4.594735
C	6.442744	5.855270	4.007259
N	7.562493	5.533506	3.370915
C	5.626197	9.611435	3.744350
H	6.084574	10.401545	4.345234
H	4.652483	9.367372	4.169687
H	5.475636	10.028173	2.742432
C	5.625964	4.765292	4.670905
H	4.630632	5.122648	4.937426
H	6.125664	4.432794	5.587241
H	5.531791	3.887795	4.026927
C	7.910241	12.769533	-0.299298
C	8.782396	13.113818	-1.347476
C	8.278480	13.844393	-2.425683
H	8.939877	14.116013	-3.243497
C	6.944425	14.226074	-2.474089
H	6.571539	14.799873	-3.316904
C	6.084393	13.845486	-1.451922
H	5.033809	14.117946	-1.506856
C	6.541589	13.103570	-0.363182
C	10.227324	12.641998	-1.356116
H	10.474780	12.307410	-0.343721
C	11.222940	13.743645	-1.733495
H	11.112607	14.617974	-1.084542
H	12.248344	13.372878	-1.637242
H	11.092090	14.075411	-2.768655
C	10.373351	11.434916	-2.292601
H	9.750931	10.596817	-1.956898
H	10.058218	11.689000	-3.310828
H	11.406901	11.081007	-2.343183
C	5.562427	12.653449	0.711051
H	6.112005	12.013835	1.408382
C	4.421487	11.816694	0.117270
H	3.786409	11.416120	0.915598
H	3.782075	12.413474	-0.541967
H	4.808634	10.976821	-0.466370
C	5.021307	13.841127	1.517264
H	5.831664	14.403175	1.991783
H	4.463123	14.532016	0.875654
H	4.344541	13.492920	2.304592
C	7.974280	4.171592	3.300056
C	7.724890	3.433503	2.123886
C	8.179409	2.114592	2.055026
H	7.979029	1.527472	1.163321

C	8.885497	1.540367	3.103245
H	9.234563	0.514991	3.029430
C	9.151576	2.288501	4.243370
H	9.721484	1.841290	5.052795
C	8.704796	3.604188	4.364709
C	6.960652	4.032652	0.950312
H	6.940610	5.116987	1.089109
C	5.502383	3.556527	0.926197
H	4.963488	3.875521	1.823730
H	4.982606	3.968890	0.054652
H	5.447588	2.463773	0.867804
C	7.638240	3.748619	-0.397277
H	8.701300	4.009359	-0.366940
H	7.561730	2.691908	-0.675880
H	7.166520	4.333600	-1.195198
C	9.050628	4.415622	5.604616
H	8.418497	5.307613	5.608833
C	10.508426	4.893542	5.540687
H	10.750576	5.511709	6.412063
H	11.195683	4.040250	5.529207
H	10.699834	5.482130	4.638643
C	8.779803	3.654044	6.907712
H	8.928113	4.314800	7.767666
H	7.754795	3.271321	6.943448
H	9.458138	2.803086	7.030388
O	8.926557	8.402104	-0.265849
Mg	10.680660	7.772350	-0.910736
Mg	7.506259	8.461641	-1.790612
H	6.860154	9.882168	-0.809521
N	12.562661	8.303697	-1.440411
C	12.920685	8.712125	-2.658043
C	12.062034	8.745070	-3.766681
H	12.470162	9.184667	-4.668151
C	10.721008	8.308053	-3.859194
N	10.102446	7.684716	-2.871631
C	14.336039	9.199544	-2.878024
H	15.049119	8.380638	-2.740768
H	14.595588	9.968192	-2.143531
H	14.464625	9.605908	-3.881560
C	9.971826	8.609766	-5.135724
H	9.597694	7.699775	-5.612320
H	10.606342	9.142947	-5.843987
H	9.093971	9.224717	-4.910373
N	8.720400	7.411424	-3.042981
C	8.378859	6.229085	-3.529460
C	7.033227	5.833216	-3.696506
H	6.886310	4.873425	-4.176261
C	5.863385	6.551470	-3.381852
N	5.856741	7.672560	-2.672111
C	9.454546	5.243945	-3.921118
H	10.095972	5.639069	-4.713693
H	9.018246	4.303488	-4.258138
H	10.105598	5.045191	-3.063372
C	4.554136	5.993576	-3.893343
H	3.871719	5.784402	-3.064449
H	4.705831	5.077915	-4.465366
H	4.057199	6.730350	-4.532427
C	13.555557	8.208392	-0.416130
C	13.652600	9.217471	0.557725
C	14.585025	9.070260	1.586164
H	14.663646	9.838672	2.350143
C	15.404637	7.953274	1.655766
H	16.123185	7.852548	2.463040
C	15.286692	6.953962	0.697716
H	15.911746	6.069063	0.772767
C	14.364803	7.056712	-0.342744
C	12.725078	10.420496	0.543485
H	12.204037	10.427614	-0.418006
C	13.471313	11.753856	0.660093
H	14.213544	11.863812	-0.136833
H	12.765910	12.588626	0.589310
H	13.991536	11.845956	1.619017
C	11.668050	10.294789	1.647510
H	11.074384	9.378075	1.549050
H	12.136834	10.260502	2.636945
H	10.980079	11.143846	1.634933
C	14.186762	5.907311	-1.323342
H	13.621635	6.279377	-2.182337
C	13.349852	4.796413	-0.670852
H	13.133636	3.996635	-1.387662
H	13.882829	4.360255	0.180512
H	12.401992	5.191762	-0.288221
C	15.513297	5.354880	-1.856096
H	16.128479	6.147073	-2.294443
H	16.100365	4.871669	-1.068278
H	15.324731	4.603157	-2.629110
C	4.653009	8.419961	-2.509357
C	3.771665	8.147648	-1.446315

C	2.639457	8.950623	-1.296426
H	1.952624	8.755323	-0.477556
C	2.383456	10.006368	-2.161119
H	1.497850	10.619653	-2.026356
C	3.281619	10.289264	-3.181630
H	3.097944	11.136072	-3.837530
C	4.426380	9.514498	-3.367763
C	4.062654	7.059889	-0.423600
H	4.912321	6.471434	-0.786992
C	2.880053	6.104001	-0.224644
H	2.584401	5.626190	-1.163948
H	3.142638	5.318475	0.490121
H	2.003842	6.627316	0.171851
C	4.475497	7.687675	0.915191
H	5.300829	8.395588	0.781710
H	3.640871	8.241219	1.361042
H	4.796712	6.924900	1.631642
C	5.435097	9.897027	-4.440281
H	6.198507	9.113555	-4.467844
C	6.131529	11.215611	-4.070253
H	6.933673	11.447646	-4.779953
H	5.423999	12.051553	-4.081327
H	6.557851	11.182368	-3.061328
C	4.807331	9.971205	-5.836496
H	5.573217	10.189444	-6.587968
H	4.326813	9.025020	-6.103906
H	4.050777	10.760977	-5.896896
C	10.966158	6.688063	2.075405
C	8.267589	7.139124	0.236988
H	7.167402	7.297859	0.205318
H	8.447639	6.355677	-0.520089
O	12.004439	6.434007	2.594537
H	11.110651	6.835114	0.795532

INT-8.log

SCF (wB97x) = -3319.22011453

E(SCF)+ZPE(0 K)= -3317.635603

H(298 K)= -3317.541078

G(298 K)= -3317.761966

Lowest Frequency = 18.6658cm-1

Mg	8.075564	9.992145	0.623501
Mg	8.670528	6.844121	2.268158
N	8.365156	11.987114	0.832959
C	8.761831	12.590988	1.949478
C	8.972236	11.916961	3.162063
H	9.407755	12.502584	3.962449
C	8.639388	10.583637	3.499237
N	8.077491	9.736296	2.652193
C	9.035064	14.078542	1.924445
H	9.838165	14.302354	1.213772
H	9.322553	14.449017	2.908818
H	8.153263	14.628388	1.583718
C	8.948305	10.129578	4.907396
H	8.036546	9.955563	5.485857
H	9.551478	10.872921	5.429561
H	9.486760	9.177634	4.881107
N	7.707617	8.462231	3.160601
C	6.501968	8.372667	3.695383
C	5.942352	7.157453	4.155943
H	4.974975	7.249158	4.634099
C	6.440815	5.848654	4.037255
N	7.557519	5.534646	3.393282
C	5.630340	9.606358	3.799266
H	6.077377	10.375896	4.434603
H	4.647567	9.352739	4.197214
H	5.503264	10.055305	2.808691
C	5.626284	4.751136	4.691505
H	4.646417	5.115536	5.001905
H	6.146113	4.370273	5.577325
H	5.495345	3.902252	4.015661
C	7.943494	12.762992	-0.286785
C	8.847440	13.085904	-1.314011
C	8.377544	13.795927	-2.421338
H	9.063959	14.049984	-3.224222
C	7.047012	14.180862	-2.515738
H	6.700394	14.738819	-3.380159
C	6.156581	13.824263	-1.510704
H	5.109197	14.099166	-1.601666
C	6.578291	13.099528	-0.396576
C	10.294795	12.624623	-1.264609
H	10.502106	12.288097	-0.243718
C	11.289635	13.743561	-1.592598
H	11.135192	14.614276	-0.947732
H	12.315633	13.389688	-1.450718
H	11.199440	14.076445	-2.631795
C	10.503490	11.424823	-2.198671

H	9.907974	10.558419	-1.883567
H	10.212250	11.670766	-3.225927
H	11.550653	11.110627	-2.218637
C	5.565130	12.666989	0.652964
H	6.085838	12.016154	1.362268
C	4.421533	11.853680	0.032131
H	3.767067	11.456245	0.816076
H	3.802149	12.466042	-0.631943
H	4.805675	11.012497	-0.551486
C	5.028260	13.864234	1.448073
H	5.836969	14.407278	1.946876
H	4.503716	14.568532	0.792749
H	4.322174	13.529201	2.215148
C	7.994869	4.182973	3.333575
C	7.766390	3.428848	2.164428
C	8.254527	2.120605	2.108745
H	8.070163	1.520388	1.222127
C	8.971160	1.573931	3.163475
H	9.344418	0.556349	3.100831
C	9.216011	2.338596	4.298828
H	9.790556	1.908501	5.113501
C	8.738179	3.643881	4.406115
C	6.995729	3.996442	0.978622
H	6.897554	5.075685	1.129175
C	5.574432	3.423462	0.902363
H	4.998718	3.665233	1.801138
H	5.042844	3.837390	0.038568
H	5.594948	2.332993	0.797304
C	7.736506	3.780167	-0.348540
H	8.768320	4.140803	-0.284524
H	7.768699	2.721098	-0.626637
H	7.236529	4.319234	-1.161566
C	9.059275	4.489502	5.631216
H	8.293590	5.265292	5.715249
C	10.402713	5.210396	5.445648
H	10.633295	5.830820	6.318427
H	11.215546	4.487697	5.314266
H	10.381934	5.858210	4.564134
C	9.044461	3.693226	6.940150
H	9.134906	4.373805	7.792416
H	8.114704	3.126612	7.052070
H	9.879217	2.986977	7.001167
O	8.908740	8.416199	-0.280339
Mg	10.643548	7.642806	-0.871067
Mg	7.508248	8.518659	-1.825570
H	6.890197	9.952941	-0.839492
N	12.535261	8.226452	-1.406823
C	12.885488	8.696946	-2.602120
C	12.033843	8.742747	-3.715437
H	12.433664	9.223772	-4.599349
C	10.700433	8.289101	-3.821413
N	10.086048	7.632468	-2.853462
C	14.280112	9.253538	-2.792528
H	15.030619	8.464729	-2.687228
H	14.499978	10.000902	-2.023572
H	14.394729	9.709921	-3.776154
C	9.950308	8.616925	-5.091607
H	9.554512	7.717588	-5.571043
H	10.592046	9.142198	-5.799451
H	9.086788	9.249747	-4.859373
N	8.698748	7.388840	-3.032759
C	8.336642	6.209163	-3.505070
C	6.983505	5.839135	-3.670127
H	6.817739	4.876302	-4.137475
C	5.829016	6.587346	-3.372396
N	5.838263	7.728953	-2.693765
C	9.395073	5.202527	-3.887817
H	10.049323	5.589526	-4.674121
H	8.943135	4.271462	-4.230650
H	10.035996	4.989048	-3.026059
C	4.512244	6.036607	-3.874618
H	3.815159	5.875795	-3.047867
H	4.650789	5.095947	-4.408103
H	4.038447	6.757277	-4.548910
C	13.526458	8.104955	-0.386974
C	13.569677	9.046250	0.656699
C	14.473138	8.852991	1.704068
H	14.509764	9.573394	2.517156
C	15.321085	7.754948	1.725207
H	16.017943	7.617053	2.546230
C	15.269362	6.830390	0.688515
H	15.925471	5.964694	0.714507
C	14.380748	6.982351	-0.375059
C	12.632201	10.241336	0.684145
H	12.074735	10.245469	-0.256632
C	13.386785	11.573253	0.765848
H	14.078846	11.688226	-0.074521
H	12.682570	12.411850	0.746540

H	13.967394	11.651310	1.691025
C	11.616672	10.121175	1.825359
H	11.039325	9.191986	1.761298
H	12.115414	10.124056	2.801208
H	10.914596	10.959249	1.814204
C	14.309775	5.913067	-1.455861
H	13.684962	6.294168	-2.267853
C	13.627457	4.648610	-0.912805
H	13.506818	3.903673	-1.707144
H	14.228417	4.196965	-0.115287
H	12.646364	4.890173	-0.494720
C	15.684302	5.580655	-2.049897
H	16.191243	6.476673	-2.421851
H	16.341444	5.109273	-1.311404
H	15.575326	4.880977	-2.884734
C	4.628074	8.466486	-2.530960
C	3.763242	8.204450	-1.451462
C	2.610317	8.979926	-1.315367
H	1.935083	8.788408	-0.485723
C	2.320596	10.004864	-2.205944
H	1.419908	10.598138	-2.081594
C	3.204707	10.283273	-3.240241
H	2.995001	11.107541	-3.916663
C	4.365831	9.530713	-3.416587
C	4.095631	7.165162	-0.391786
H	4.987629	6.621006	-0.719947
C	2.974322	6.140626	-0.181752
H	2.734243	5.606110	-1.106036
H	3.273185	5.402411	0.569063
H	2.055727	6.619207	0.173831
C	4.436304	7.864556	0.931862
H	5.194514	8.639465	0.776621
H	3.551541	8.355947	1.353137
H	4.815806	7.155162	1.674399
C	5.358706	9.906746	-4.505374
H	6.118530	9.120159	-4.541732
C	6.063944	11.222208	-4.141137
H	6.858088	11.455358	-4.859413
H	5.357048	12.059061	-4.139974
H	6.501549	11.183090	-3.137336
C	4.714536	9.985305	-5.893570
H	5.474054	10.192720	-6.654440
H	4.219711	9.044362	-6.153221
H	3.967088	10.784101	-5.948231
C	10.771398	6.216949	1.817085
C	8.102443	7.240117	0.220137
H	7.030184	7.528904	0.191642
H	8.198029	6.436881	-0.529613
O	11.148259	6.414068	0.638995
H	11.526380	5.601854	2.371771

INT-9.log

SCF (wB97x) = -3319.24682417

E(SCF)+ZPE(0 K)= -3317.657784

H(298 K)= -3317.564673

G(298 K)= -3317.783507

Lowest Frequency = 11.2721cm-1

Mg	7.542853	9.801916	0.383524
Mg	8.701157	6.204895	2.096494
N	7.968821	11.750838	0.838146
C	8.308696	12.194895	2.044763
C	8.434222	11.374198	3.172587
H	8.788641	11.868824	4.068212
C	8.204875	9.988311	3.315446
N	7.737554	9.191096	2.360433
C	8.641449	13.659767	2.235564
H	9.579760	13.898442	1.722974
H	8.752335	13.906323	3.291832
H	7.873905	14.301896	1.797437
C	8.554768	9.403898	4.666963
H	7.663670	9.106329	5.228209
H	9.112520	10.124955	5.265009
H	9.152236	8.500012	4.534077
N	7.590211	7.832292	2.761226
C	6.493117	7.533584	3.439304
C	6.334229	6.323525	4.157268
H	5.377912	6.204299	4.651872
C	7.305687	5.350890	4.470994
N	8.484059	5.257213	3.866200
C	5.352425	8.526502	3.505884
H	5.602348	9.387588	4.132963
H	4.456710	8.055366	3.912258
H	5.128923	8.914404	2.507153
C	6.975106	4.379662	5.581893
H	5.956562	4.517770	5.945324
H	7.667079	4.523947	6.418584

H	7.098275	3.346472	5.244560	H	8.598084	0.434827	3.070186
C	7.811666	12.699108	-0.218350	C	9.224855	2.498431	1.303854
C	8.920352	13.079001	-0.996249	H	9.655169	3.459325	1.009629
C	8.729805	13.985749	-2.041219	H	10.027826	1.752177	1.311776
H	9.577569	14.285206	-2.651095	H	8.498618	2.212864	0.536075
C	7.474038	14.512357	-2.312538	C	10.386803	6.154099	5.833526
H	7.342928	15.223326	-3.122511	H	9.380936	6.551966	5.670658
C	6.382366	14.107637	-1.554535	C	11.370667	7.048315	5.064773
H	5.397017	14.502572	-1.785272	H	11.350658	8.075834	5.445297
C	6.524401	13.189038	-0.514732	H	12.393491	6.667883	5.161051
C	10.291102	12.466284	-0.760538	H	11.136614	7.076090	3.995117
H	10.279913	11.991040	0.223707	C	10.659663	6.206901	7.340215
C	11.429491	13.491732	-0.757995	H	10.502921	7.223735	7.714120
H	11.235063	14.302315	-0.048875	H	9.992154	5.534639	7.887892
H	12.369337	13.006727	-0.474341	H	11.690768	5.927495	7.580975
H	11.578778	13.939876	-1.745891	O	8.047050	8.258352	-0.809481
C	10.551806	11.368115	-1.797075	Mg	9.959011	7.575456	-1.120667
H	9.784803	10.585900	-1.749670	Mg	7.152018	9.050607	-2.440791
H	10.534683	11.775394	-2.814243	H	6.612019	10.335535	-1.235823
H	11.523124	10.889043	-1.649020	N	11.993159	7.819558	-1.229006
C	5.302924	12.733006	0.267163	C	12.673794	8.341283	-2.243033
H	5.608455	11.869639	0.866955	C	12.082710	8.793803	-3.434409
C	4.165086	12.277896	-0.653670	H	12.742721	9.291159	-4.134443
H	3.351298	11.841304	-0.064789	C	10.735026	8.685833	-3.838957
H	3.741584	13.112206	-1.223114	N	9.819126	8.055364	-3.122294
H	4.511748	11.523711	-1.364833	C	14.175863	8.477110	-2.124908
C	4.826436	13.816347	1.244417	H	14.637802	7.490006	-2.021437
H	5.607711	14.078117	1.964349	H	14.441355	9.043600	-1.226473
H	4.543253	14.728141	0.706723	H	14.603105	8.972368	-2.997426
H	3.952188	13.470260	1.805877	C	10.341706	9.345097	-5.140673
C	9.466149	4.327012	4.316196	H	9.971370	8.618501	-5.869053
C	9.539781	3.046234	3.735943	H	11.186097	9.878919	-5.577647
C	10.577789	2.196711	4.124932	H	9.524456	10.052037	-4.964482
H	10.649637	1.207598	3.680724	N	8.491497	8.048398	-3.629551
C	11.522638	2.594717	5.060447	C	8.139001	7.024587	-4.384936
H	12.323760	1.920832	5.348343	C	6.818397	6.844242	-4.864791
C	11.441045	3.863004	5.622426	H	6.688157	6.026018	-5.562211
H	12.188925	4.174654	6.345770	C	5.664597	7.587000	-4.572343
C	10.421580	4.744675	5.264337	N	5.604159	8.549278	-3.651424
C	8.547127	2.588115	2.678501	C	9.163770	5.976320	-4.753387
H	7.759264	3.343627	2.603632	H	10.010306	6.406371	-5.295279
C	7.876718	1.258803	3.048372	H	8.716353	5.191746	-5.363931
H	7.399969	1.309886	4.032372	H	9.572861	5.525771	-3.842922
H	7.108755	1.005515	2.310743	C	4.418446	7.252866	-5.362455

H	3.584108	7.020222	-4.694713
H	4.584744	6.408565	-6.031780
H	4.110517	8.117458	-5.959726
C	12.691489	7.265349	-0.113647
C	12.897427	8.040229	1.041902
C	13.456954	7.434721	2.167481
H	13.605275	8.018612	3.071670
C	13.804964	6.089817	2.159116
H	14.223153	5.627917	3.048251
C	13.606291	5.337002	1.008301
H	13.871703	4.283405	1.008304
C	13.056230	5.905907	-0.140879
C	12.426661	9.482220	1.097987
H	12.263107	9.814213	0.069249
C	13.441108	10.439734	1.728881
H	14.410664	10.381751	1.224548
H	13.078051	11.470634	1.656373
H	13.600384	10.224810	2.790865
C	11.079805	9.548986	1.825683
H	10.355262	8.852351	1.388903
H	11.194976	9.263380	2.878265
H	10.655309	10.557128	1.805984
C	12.797337	5.047479	-1.368995
H	12.528750	5.716165	-2.191752
C	11.599032	4.120084	-1.122262
H	11.329702	3.577221	-2.035257
H	11.834786	3.381567	-0.347512
H	10.734365	4.692078	-0.770285
C	14.034226	4.256132	-1.808766
H	14.890155	4.916646	-1.980307
H	14.328688	3.514008	-1.059111
H	13.827653	3.717557	-2.739352
C	4.397613	9.295284	-3.492056
C	3.446903	8.911870	-2.527397
C	2.296552	9.687282	-2.371939
H	1.555734	9.398352	-1.631033
C	2.089632	10.827696	-3.135629
H	1.190481	11.420728	-3.000034
C	3.051718	11.217489	-4.058865
H	2.901627	12.126815	-4.634253
C	4.213010	10.468986	-4.250492
C	3.674059	7.712494	-1.622332
H	4.597603	7.225407	-1.947467
C	2.549247	6.675360	-1.717739
H	2.415090	6.327711	-2.746489
H	2.779416	5.806571	-1.092954
H	1.593531	7.087485	-1.376598
C	3.874798	8.172679	-0.170478
H	4.650582	8.944449	-0.120210
H	2.954898	8.607729	0.235972
H	4.168154	7.334242	0.470152
C	5.286139	10.958268	-5.210455
H	6.006174	10.146415	-5.346212
C	6.037423	12.145666	-4.590206
H	6.873770	12.457915	-5.225203
H	5.370088	13.005506	-4.463610
H	6.428412	11.905088	-3.596021
C	4.733094	11.315782	-6.594341
H	5.551243	11.580195	-7.271922
H	4.185948	10.475445	-7.032803
H	4.053099	12.173225	-6.551897
O	9.608730	6.516211	0.438963
C	8.285947	5.929437	0.056162
H	8.442035	5.209616	-0.761170
C	7.344491	7.049240	-0.367152
H	6.681032	7.364985	0.453889
H	6.682741	6.684510	-1.171325

1.log

SCF (wB97x) = -3092.52231834
E(SCF)+ZPE(0 K)= -3090.969684
H(298 K)= -3090.879102
G(298 K)= -3091.093762
Lowest Frequency = -54.5723cm-1

Mg	8.754467	0.177451	4.134244
N	9.320613	-1.740026	4.569765
N	10.098900	0.334430	2.653005
C	9.575384	-4.023768	3.705644
C	9.772859	-2.527718	3.603910
C	10.420461	-2.040686	2.442782
C	10.637316	-0.710357	2.039955
C	11.519642	-0.475062	0.835790
C	10.374431	1.672689	2.255193
C	11.462903	2.355146	2.835164
C	11.654333	3.699609	2.517804
C	10.787197	4.367113	1.661288

C	9.699418	3.692404	1.123552	C	2.598314	-3.821878	8.597661
C	9.467499	2.346238	1.413752	C	3.272890	-2.734124	9.135613
C	12.365559	1.675021	3.853122	C	4.619118	-2.502164	8.845729
C	12.050578	2.189248	5.267142	C	5.290964	-5.400162	6.406438
C	13.853889	1.836900	3.523758	C	4.777271	-5.085120	4.992239
C	8.223128	1.660748	0.867476	C	5.128907	-6.888497	6.735706
C	8.186486	1.660179	-0.665339	C	5.304455	-1.257829	9.392284
C	6.951470	2.296733	1.446485	C	5.304516	-1.221302	10.925103
H	10.147078	-4.466764	4.525305	C	4.668655	0.013873	8.813168
H	9.866485	-4.514418	2.776592	H	11.432198	-3.181644	5.734078
H	8.520991	-4.235799	3.908941	H	11.479954	-2.901253	7.482822
H	10.808085	-2.802897	1.777924	H	11.201287	-1.555630	6.350645
H	11.002114	0.139619	0.093026	H	9.768479	-3.842839	8.481541
H	11.828015	-1.413008	0.373025	H	6.826091	-4.036861	10.166619
H	12.416391	0.078346	1.134197	H	8.378588	-4.862930	9.886398
H	12.485364	4.239604	2.963372	H	6.887093	-5.451040	9.125296
H	10.949714	5.414817	1.427714	H	2.726145	-5.519994	7.295543
H	9.008667	4.223722	0.473963	H	1.550560	-3.984422	8.830993
H	12.138502	0.605262	3.835848	H	2.741419	-2.043427	9.785134
H	12.265942	3.260185	5.352798	H	6.360730	-5.173173	6.424080
H	12.653597	1.664581	6.016233	H	3.706318	-5.300265	4.906219
H	10.993403	2.051051	5.516680	H	5.302070	-5.688261	4.243339
H	14.079203	1.482024	2.513057	H	4.915776	-4.027981	4.742722
H	14.462830	1.263756	4.230224	H	5.483471	-7.113866	7.746504
H	14.172332	2.882782	3.589764	H	5.702220	-7.497453	6.029390
H	8.243444	0.616535	1.194390	H	4.083023	-7.206864	6.669384
H	7.295990	1.133315	-1.023800	H	6.348771	-1.278131	9.065700
H	9.068145	1.165626	-1.084882	H	5.831228	-0.330819	11.283816
H	8.151877	2.679004	-1.065972	H	5.798951	-2.102981	11.344742
H	6.849248	3.341100	1.130630	H	4.285555	-1.186754	11.325396
H	6.961396	2.275642	2.541127	H	3.624191	0.116078	9.128709
H	6.056491	1.758195	1.117740	H	4.690084	0.004030	7.718533
H	8.668605	1.703082	5.129793	H	5.207096	0.908824	9.142149
H	6.965497	0.000067	3.773245	Mg	5.176500	-0.177477	4.134114
N	8.705471	-2.355210	5.689827	N	4.610311	1.739989	4.569750
N	6.631147	-3.133430	7.606704	N	3.832089	-0.334313	2.652849
C	10.989254	-2.610044	6.553831	C	4.355519	4.023780	3.705755
C	9.493205	-2.807494	6.655629	C	4.158041	2.527734	3.603951
C	9.006235	-3.455143	7.816762	C	3.510393	2.040780	2.442811
C	7.675929	-3.671962	8.219659	C	3.293568	0.710479	2.039900
C	7.440639	-4.554392	9.423747	C	2.411160	0.475207	0.835793
C	5.292865	-3.409039	8.004377	C	3.556511	-1.672585	2.255126
C	4.610556	-4.497507	7.424218	C	2.468040	-2.354948	2.835216
C	3.266022	-4.688965	7.741256	C	2.276626	-3.699470	2.518101

C	3.143752	-4.367111	1.661683
C	4.231503	-3.692480	1.123793
C	4.463423	-2.346263	1.413759
C	1.565321	-1.674615	3.852988
C	1.880303	-2.188373	5.267175
C	0.077007	-1.836701	3.523635
C	5.707764	-1.660857	0.867301
C	5.744277	-1.660527	-0.665519
C	6.979464	-2.296774	1.446296
H	3.783918	4.466734	4.525503
H	4.064329	4.514487	2.776761
H	5.409935	4.235791	3.908952
H	3.122711	2.803033	1.778034
H	2.928702	-0.139332	0.092922
H	2.102631	1.413163	0.373150
H	1.514505	-0.078341	1.134223
H	1.445596	-4.239390	2.963760
H	2.981240	-5.414857	1.428291
H	4.922233	-4.223901	0.474266
H	1.792279	-0.604842	3.835414
H	1.665249	-3.259351	5.353112
H	1.277059	-1.663677	6.016065
H	2.937410	-2.049784	5.516778
H	-0.148325	-1.482075	2.512851
H	-0.532004	-1.263460	4.229961
H	-0.241324	-2.882602	3.589868
H	5.687477	-0.616596	1.194062
H	6.634728	-1.133701	-1.024146
H	4.862569	-1.166063	-1.085062
H	5.778876	-2.679413	-1.065994
H	7.081647	-3.341187	1.130581
H	6.969637	-2.275527	2.540936
H	7.874420	-1.758295	1.117392
H	5.262452	-1.703025	5.129793
N	5.225504	2.355121	5.689812
N	7.299973	3.133402	7.606562
C	2.941766	2.609941	6.553936
C	4.437821	2.807390	6.655665
C	4.924863	3.455004	7.816781
C	6.255197	3.671850	8.219601
C	6.490509	4.554219	9.423731
C	8.638242	3.408965	8.004325
C	9.320643	4.497424	7.424263
C	10.665119	4.688903	7.741534

C	11.332692	3.821829	8.598057
C	10.658039	2.734061	9.135887
C	9.311863	2.502090	8.845774
C	8.640431	5.400009	6.406301
C	9.154555	5.084945	4.992261
C	8.802312	6.888362	6.735560
C	8.626447	1.257743	9.392195
C	8.626115	1.221172	10.925012
C	9.262350	-0.013940	8.813151
H	2.498777	3.181635	5.734271
H	2.451125	2.901058	7.482987
H	2.729718	1.555550	6.350650
H	4.162659	3.842657	8.481630
H	7.105202	4.036720	10.166504
H	5.552568	4.862608	9.886498
H	7.043910	5.450959	9.125282
H	11.205067	5.519925	7.295893
H	12.380405	3.984385	8.831566
H	11.189407	2.043365	9.785493
H	7.570677	5.172938	6.423668
H	10.225481	5.300321	4.906507
H	8.629818	5.687906	4.243172
H	9.016355	4.027751	4.742804
H	8.447512	7.113731	7.746276
H	8.229102	7.497250	6.029103
H	9.848184	7.206818	6.669453
H	7.582187	1.278040	9.065435
H	8.099366	0.330658	11.283596
H	8.131570	2.102819	11.344590
H	9.645005	1.186649	11.325487
H	10.306763	-0.116149	9.128859
H	9.241098	-0.004060	7.718512
H	8.723859	-0.908903	9.142013
Mg	7.142971	1.788948	6.125342
H	6.965583	-0.000014	6.486362
Mg	6.788010	-1.789001	6.125457

2.log

SCF (wB97x) = -3319.37318181
E(SCF)+ZPE(0 K)= -3317.783115
H(298 K)= -3317.690111
G(298 K)= -3317.905271
Lowest Frequency = 20.6677cm-1

Mg	8.010372	10.156070	0.537183
Mg	8.516997	7.208539	1.766035
N	8.313964	12.171041	0.782748
C	8.643407	12.780919	1.920181
C	8.785958	12.120667	3.149527
H	9.179706	12.708859	3.969473
C	8.463550	10.777646	3.454328
N	7.922088	9.970610	2.563649
C	8.914941	14.269603	1.903920
H	9.741807	14.499122	1.223553
H	9.163951	14.640064	2.898804
H	8.043613	14.816671	1.532928
C	8.794041	10.267985	4.837796
H	7.898986	10.019737	5.414425
H	9.368789	11.008777	5.394573
H	9.378190	9.345968	4.755381
N	7.600595	8.649727	2.959114
C	6.435793	8.487620	3.566285
C	5.958190	7.224168	3.971263
H	5.026121	7.228538	4.522370
C	6.504190	5.966262	3.689273
N	7.555863	5.766175	2.896036
C	5.527757	9.675134	3.799222
H	5.966580	10.421069	4.466136
H	4.574730	9.351704	4.218732
H	5.342758	10.181078	2.845586
C	5.807881	4.771835	4.305876
H	4.936045	5.077527	4.884945
H	6.489102	4.224086	4.964353
H	5.490571	4.069051	3.528590
C	8.008029	12.955319	-0.368363
C	9.003498	13.234479	-1.322049
C	8.649345	13.932113	-2.478510
H	9.407538	14.146464	-3.226783
C	7.344656	14.358963	-2.687882
H	7.086521	14.904417	-3.590376
C	6.369517	14.072015	-1.741381
H	5.345139	14.390665	-1.915867
C	6.674321	13.359564	-0.581673
C	10.433602	12.759315	-1.126846
H	10.532357	12.425061	-0.088947
C	11.463720	13.872048	-1.353097
H	11.249710	14.746003	-0.730068
H	12.468202	13.513104	-1.106126
H	11.481804	14.202179	-2.396995
C	10.732132	11.555683	-2.027551
H	10.084413	10.701448	-1.795893
H	10.578850	11.803129	-3.083755
H	11.767943	11.225258	-1.911561
C	5.567570	13.016845	0.405202
H	6.016418	12.437786	1.217923
C	4.493652	12.138552	-0.249950
H	3.741704	11.834103	0.486504
H	3.971875	12.673656	-1.051139
H	4.932341	11.237803	-0.688111
C	4.948401	14.276531	1.024722
H	5.702726	14.887501	1.530136
H	4.469411	14.899469	0.261220
H	4.184351	14.004455	1.760435
C	8.061666	4.437772	2.771845
C	7.666223	3.638012	1.685492
C	8.252502	2.381977	1.531230
H	7.968108	1.758218	0.688318
C	9.203127	1.920586	2.431433
H	9.655271	0.943033	2.293870
C	9.569770	2.711659	3.513450
H	10.307265	2.339449	4.218794
C	9.005719	3.972412	3.708969
C	6.650728	4.145641	0.675167
H	6.164619	5.021643	1.112268
C	5.547831	3.128408	0.365203
H	5.039110	2.801042	1.277451
H	4.802593	3.576577	-0.300789
H	5.941527	2.238926	-0.137711
C	7.341107	4.602689	-0.614122
H	8.104721	5.360921	-0.412293
H	7.848207	3.764482	-1.104893
H	6.621487	5.019498	-1.324092
C	9.405364	4.825015	4.907032
H	8.628519	5.581348	5.048027
C	10.718968	5.578546	4.654741
H	10.974480	6.204769	5.516238
H	11.545592	4.877929	4.490047
H	10.649218	6.225867	3.775204
C	9.489758	4.013952	6.205552
H	9.638899	4.684043	7.058083
H	8.573274	3.440923	6.377496

H	10.328574	3.309978	6.194692	C	13.298624	11.428039	1.117563
O	8.603745	8.309551	0.099024	H	14.080475	11.467751	0.352793
Mg	10.337736	7.526504	-0.539681	H	12.645642	12.297150	0.985920
Mg	7.382749	8.301209	-1.484306	H	13.782858	11.527625	2.094591
H	6.911499	10.037504	-0.977355	C	11.390669	10.106895	2.092510
N	12.312921	7.992983	-0.944008	H	10.773069	9.206012	2.010147
C	12.715138	8.572840	-2.074813	H	11.825730	10.110155	3.098398
C	11.931661	8.692511	-3.232580	H	10.738060	10.980220	2.011568
H	12.370785	9.262605	-4.042013	C	13.914431	5.577485	-0.792616
C	10.601655	8.268893	-3.438851	H	12.874447	5.562901	-1.132980
N	9.945092	7.543397	-2.550590	C	14.228972	4.195379	-0.209922
C	14.067366	9.257872	-2.123312	H	13.927318	3.418932	-0.919895
H	14.839465	8.691380	-1.601301	H	15.301513	4.066224	-0.025059
H	13.990939	10.229585	-1.620625	H	13.693586	4.023758	0.727242
H	14.385424	9.432277	-3.152065	C	14.803977	5.826617	-2.021117
C	9.904129	8.735979	-4.695662	H	14.473387	6.691302	-2.599383
H	9.543741	7.903543	-5.305009	H	15.845530	5.988951	-1.720857
H	10.568680	9.351960	-5.302048	H	14.776201	4.956936	-2.686267
H	9.022552	9.326707	-4.423415	C	4.545124	8.324723	-2.295047
N	8.573439	7.295115	-2.800740	C	3.650446	7.975897	-1.266900
C	8.247923	6.228110	-3.504473	C	2.520016	8.769699	-1.063126
C	6.902986	5.904300	-3.793378	H	1.824647	8.515682	-0.267875
H	6.751933	5.036586	-4.423881	C	2.267131	9.877974	-1.860606
C	5.747716	6.582938	-3.378396	H	1.379966	10.480761	-1.692459
N	5.745086	7.579540	-2.493987	C	3.164260	10.218825	-2.864366
C	9.327377	5.305217	-4.017994	H	2.975210	11.098434	-3.474181
H	9.938271	5.783302	-4.789157	C	4.317088	9.466752	-3.089733
H	8.893644	4.396021	-4.435518	C	3.917313	6.785551	-0.360113
H	10.008268	5.041281	-3.203070	H	4.706180	6.184269	-0.822654
C	4.432173	6.114622	-3.962619	C	2.692795	5.878928	-0.189684
H	3.764418	5.766673	-3.167315	H	2.297607	5.556461	-1.158061
H	4.580108	5.304625	-4.677474	H	2.962322	4.986700	0.384819
H	3.917091	6.937890	-4.465629	H	1.884572	6.382814	0.350539
C	13.245924	7.868911	0.129783	C	4.434001	7.255881	1.004581
C	13.341089	8.884466	1.098324	H	5.390278	7.787001	0.919137
C	14.220304	8.717444	2.169929	H	3.723456	7.940834	1.480433
H	14.294234	9.493439	2.927098	H	4.586401	6.413297	1.684153
C	15.001874	7.577150	2.280241	C	5.303518	9.905400	-4.162674
H	15.687533	7.462067	3.114203	H	6.141240	9.201715	-4.151999
C	14.898187	6.578137	1.319225	C	5.867156	11.299954	-3.858989
H	15.507511	5.686020	1.419974	H	6.633224	11.574586	-4.592685
C	14.021075	6.693010	0.239678	H	5.084637	12.066034	-3.897236
C	12.482097	10.134181	1.019784	H	6.315051	11.337615	-2.862091
H	11.985136	10.137556	0.045747	C	4.679830	9.852182	-5.563452

H 5.417997 10.133836 -6.321653
H 4.316503 8.847592 -5.801390
H 3.834015 10.543461 -5.647881
O 10.173641 6.343086 1.043650
C 10.803633 5.156691 1.106123
H 11.319588 4.952556 2.043839
C 10.831419 4.264765 0.112263
H 10.300203 4.434917 -0.820694
H 11.367482 3.331983 0.231137

TS1.log

SCF (wB97x) = -3205.80575720
E(SCF)+ZPE(0 K)= -3204.244684
H(298 K)= -3204.150871
G(298 K)= -3204.371743
Lowest Frequency = -321.4695cm-1

Mg	7.670742	9.991443	0.447081
Mg	8.226848	6.416044	1.601402
N	8.007732	11.915607	0.901993
C	8.448356	12.326899	2.084145
C	8.648798	11.472061	3.182080
H	9.100568	11.929095	4.053855
C	8.347765	10.096624	3.318205
N	7.759435	9.377353	2.371136
C	8.765193	13.792559	2.277517
H	9.433031	14.149039	1.487408
H	9.226486	13.978312	3.247883
H	7.848724	14.387920	2.208158
C	8.753550	9.435617	4.615481
H	7.891210	9.211970	5.250595
H	9.433346	10.077285	5.176832
H	9.243336	8.483072	4.403687
N	7.508009	8.012947	2.650700
C	6.461119	7.730358	3.422943
C	6.251513	6.456377	3.996163
H	5.354664	6.361016	4.596394
C	7.151068	5.365973	4.053841
N	8.201711	5.254716	3.257746
C	5.448926	8.809270	3.739010
H	5.818722	9.504065	4.499002
H	4.524698	8.363917	4.109442
H	5.233187	9.400096	2.844567
C	6.898290	4.304109	5.098763
H	5.956705	4.472180	5.622171
H	7.711324	4.309427	5.832760
H	6.885495	3.308190	4.646343
C	7.745782	12.837812	-0.152458
C	8.687887	12.994907	-1.186061
C	8.387522	13.859375	-2.240690
H	9.110193	13.994460	-3.041194
C	7.175248	14.532429	-2.293770
H	6.956075	15.198366	-3.122765
C	6.231034	14.325905	-1.294957
H	5.267263	14.822474	-1.361913
C	6.491747	13.478813	-0.218387
C	9.988302	12.207239	-1.206501
H	10.018572	11.590421	-0.304210
C	11.221157	13.116960	-1.176850
H	11.235772	13.736634	-0.274368
H	12.136154	12.515797	-1.194448
H	11.246689	13.785850	-2.043716
C	10.033073	11.258559	-2.410328
H	9.169422	10.585596	-2.411001
H	10.023688	11.811108	-3.356590
H	10.936288	10.639494	-2.389840
C	5.407075	13.188931	0.808092
H	5.877652	12.691450	1.660904
C	4.377420	12.212664	0.216028
H	3.625242	11.938996	0.964523
H	3.858783	12.662204	-0.637566
H	4.854673	11.297065	-0.148156
C	4.726782	14.456853	1.335971
H	5.458416	15.167291	1.733940
H	4.154754	14.967412	0.554072
H	4.026788	14.202922	2.138326
C	9.183175	4.241125	3.452036
C	9.083186	3.016088	2.766722
C	10.117256	2.087986	2.904253
H	10.055498	1.140347	2.376311
C	11.229398	2.360978	3.688464
H	12.026166	1.629051	3.778975
C	11.324996	3.582193	4.344443
H	12.206763	3.800834	4.939669
C	10.313530	4.536446	4.240106
C	7.914524	2.714978	1.842327
H	7.164483	3.498686	1.982646

C	7.239687	1.375305	2.159889
H	6.922394	1.327505	3.206250
H	6.355862	1.239079	1.528923
H	7.910863	0.529734	1.975621
C	8.368893	2.765661	0.375513
H	8.836673	3.727516	0.137505
H	9.107723	1.982601	0.171185
H	7.519079	2.621273	-0.299102
C	10.465601	5.899110	4.896109
H	9.474132	6.359210	4.932934
C	11.369196	6.790041	4.031715
H	11.469445	7.792728	4.461699
H	12.370261	6.358380	3.947615
H	10.982581	6.893274	3.011482
C	10.987015	5.825291	6.334783
H	10.965619	6.819943	6.791702
H	10.374585	5.155197	6.945909
H	12.022091	5.470302	6.378137
Mg	10.080958	7.525254	-0.774250
Mg	7.010229	8.326727	-1.846993
H	8.873473	8.864827	-0.412116
N	12.075638	7.727660	-1.004229
C	12.640207	8.080789	-2.159013
C	11.933210	8.300771	-3.351887
H	12.514523	8.699413	-4.174401
C	10.554811	8.110376	-3.627294
N	9.735532	7.516512	-2.780086
C	14.134979	8.315751	-2.199346
H	14.672585	7.449638	-1.803166
H	14.403485	9.167759	-1.566071
H	14.479918	8.511915	-3.214759
C	10.020690	8.663352	-4.928208
H	9.629288	7.882366	-5.585043
H	10.795960	9.212720	-5.462800
H	9.185575	9.338223	-4.713078
N	8.360411	7.454858	-3.114583
C	7.954854	6.446634	-3.880497
C	6.622955	6.296752	-4.324029
H	6.447280	5.457637	-4.986035
C	5.512819	7.135082	-4.086296
N	5.520285	8.110443	-3.193406
C	8.948943	5.389082	-4.306527
H	9.695115	5.782422	-5.002297
H	8.439923	4.552647	-4.786088
H	9.497616	5.024622	-3.432890
C	4.268020	6.899874	-4.910569
H	3.386943	6.823773	-4.266852
H	4.351087	5.996122	-5.515071
H	4.100792	7.751737	-5.578754
C	12.889777	7.496690	0.143900
C	13.242551	8.566605	0.991695
C	14.045470	8.302762	2.102264
H	14.327194	9.116795	2.763755
C	14.489536	7.016760	2.381106
H	15.124726	6.833754	3.242479
C	14.085506	5.962908	1.573533
H	14.400073	4.951393	1.815543
C	13.269540	6.176902	0.461473
C	12.721559	9.977203	0.755396
H	12.325016	10.027118	-0.264325
C	13.816113	11.043599	0.880356
H	14.660794	10.830268	0.217927
H	13.416351	12.028239	0.620085
H	14.201924	11.111566	1.902674
C	11.562293	10.278446	1.717015
H	10.719689	9.594058	1.566116
H	11.882800	10.180256	2.760221
H	11.186932	11.297073	1.577016
C	12.804315	4.988493	-0.365325
H	12.100456	5.362545	-1.114775
C	12.064866	3.962345	0.504639
H	11.634921	3.170669	-0.118318
H	12.734061	3.485114	1.227965
H	11.252094	4.429276	1.069314
C	13.968701	4.338179	-1.123984
H	14.458418	5.055482	-1.789963
H	14.724189	3.952233	-0.430629
H	13.611326	3.500433	-1.731780
C	4.428643	9.013218	-3.058319
C	3.476085	8.814099	-2.041451
C	2.467438	9.765290	-1.876107
H	1.723432	9.621531	-1.096866
C	2.407747	10.897151	-2.677665
H	1.617539	11.627670	-2.533047
C	3.379499	11.102343	-3.650093
H	3.350046	12.006883	-4.250966
C	4.402518	10.176758	-3.852950
C	3.554701	7.621549	-1.100725

H 4.390083 6.993968 -1.423511
C 2.290175 6.756126 -1.153769
H 2.085261 6.417528 -2.174119
H 2.409542 5.872039 -0.519526
H 1.411178 7.306788 -0.800689
C 3.850946 8.075745 0.337112
H 4.776688 8.659304 0.374343
H 3.042416 8.701877 0.731267
H 3.965600 7.211355 0.999888
C 5.522610 10.470095 -4.839323
H 6.071487 9.539688 -5.009296
C 6.504577 11.475223 -4.217535
H 7.376415 11.627010 -4.864112
H 6.023785 12.446752 -4.061958
H 6.856177 11.138465 -3.237076
C 5.017064 10.960195 -6.200047
H 5.854412 11.069198 -6.896844
H 4.300560 10.256372 -6.635174
H 4.526133 11.936391 -6.125668
C 6.572666 5.702213 0.308084
H 6.550763 9.872222 -0.967252
H 9.661185 6.315589 0.519381
O 5.709693 5.137239 -0.218440
H 6.882214 7.085402 -0.402577

TS2.log

SCF (wB97x) = -3205.80945274
E(SCF)+ZPE(0 K)= -3204.243553
H(298 K)= -3204.150932
G(298 K)= -3204.367918
Lowest Frequency = -103.8609cm-1

Mg 7.501420 9.931242 0.594296
Mg 8.281940 6.459988 1.728933
N 7.962652 11.862592 0.906748
C 8.486039 12.329382 2.038325
C 8.756586 11.536390 3.163990
H 9.273589 12.035198 3.974483
C 8.493333 10.162938 3.378379
N 7.825460 9.407863 2.519393
C 8.865168 13.791281 2.121316
H 9.670096 14.012897 1.412336
H 9.197948 14.061125 3.123786
H 8.019391 14.426609 1.843931
C 9.038508 9.544573 4.644772
H 8.245944 9.302829 5.359091
H 9.743805 10.220368 5.129405
H 9.540029 8.603858 4.403006
N 7.624474 8.050411 2.867570
C 6.629009 7.782168 3.705137
C 6.432505 6.509044 4.285512
H 5.585981 6.430392 4.956866
C 7.292416 5.388862 4.242842
N 8.273179 5.262887 3.364181
C 5.652617 8.873196 4.088694
H 6.107084 9.612535 4.754537
H 4.784428 8.448707 4.593800
H 5.320331 9.413715 3.197444
C 7.083184 4.307574 5.278065
H 6.167404 4.470173 5.847288
H 7.928024 4.296563 5.975404
H 7.046468 3.320451 4.808187
C 7.698080 12.749174 -0.180464
C 8.701318 13.012469 -1.133681
C 8.408744 13.865376 -2.198082
H 9.173907 14.076572 -2.939910
C 7.149704 14.435919 -2.334767
H 6.939685 15.101812 -3.166134
C 6.152449 14.122419 -1.421386
H 5.157605 14.539947 -1.550997
C 6.400352 13.270764 -0.343566
C 10.047515 12.310585 -1.068089
H 10.133229 11.846444 -0.082516
C 11.241691 13.254778 -1.235226
H 11.225072 14.057061 -0.490965
H 12.177689 12.698703 -1.118180
H 11.260087 13.718178 -2.227126
C 10.094008 11.185649 -2.110258
H 9.266972 10.479969 -1.971121
H 10.008642 11.588472 -3.125944
H 11.028606 10.619258 -2.051106
C 5.268245 12.904797 0.602889
H 5.665756 12.191470 1.331723
C 4.122484 12.215308 -0.151638
H 3.357558 11.862673 0.548980
H 3.635929 12.899543 -0.854820
H 4.483997 11.357046 -0.725767

C	4.766840	14.123688	1.387887	H	14.317009	9.266202	-1.562482
H	5.574904	14.586406	1.963171	H	14.354863	8.617989	-3.215652
H	4.354993	14.884374	0.715624	C	9.855425	8.653563	-4.812749
H	3.976276	13.831467	2.086765	H	9.459488	7.851686	-5.441272
C	9.226799	4.212141	3.465180	H	10.606871	9.203018	-5.380224
C	9.064177	3.041818	2.701597	H	9.014650	9.319910	-4.592049
C	10.059152	2.063876	2.759037	N	8.274699	7.428886	-2.961241
H	9.947781	1.155674	2.172794	C	7.903913	6.399857	-3.718928
C	11.194789	2.240472	3.537391	C	6.592066	6.236818	-4.203086
H	11.960732	1.471486	3.567159	H	6.433069	5.372385	-4.835752
C	11.356826	3.415039	4.263058	C	5.480327	7.084602	-4.027799
H	12.260378	3.559355	4.848222	N	5.457803	8.088104	-3.162686
C	10.386102	4.415938	4.240112	C	8.922311	5.347796	-4.093502
C	7.869557	2.860516	1.778878	H	9.687330	5.737644	-4.770819
H	7.175124	3.685977	1.962531	H	8.437128	4.497910	-4.573776
C	7.108521	1.557606	2.050246	H	9.442865	5.002301	-3.195390
H	6.783260	1.497622	3.093619	C	4.274300	6.839314	-4.904592
H	6.221387	1.497793	1.412035	H	3.357813	6.809891	-4.309664
H	7.727049	0.678059	1.841442	H	4.370177	5.908072	-5.463544
C	8.309626	2.946733	0.309176	H	4.162885	7.663103	-5.618143
H	8.872653	3.866448	0.117570	C	12.884643	7.536747	0.167542
H	8.958765	2.102975	0.047740	C	13.285674	8.586057	1.017150
H	7.441914	2.942895	-0.356357	C	14.134698	8.294922	2.085539
C	10.613729	5.734838	4.962840	H	14.453533	9.093504	2.748786
H	9.644579	6.232511	5.062011	C	14.578755	6.999789	2.318883
C	11.516258	6.638572	4.109941	H	15.249335	6.794145	3.147642
H	11.672614	7.610287	4.592088	C	14.133211	5.966096	1.507070
H	12.496590	6.176651	3.960342	H	14.452008	4.948016	1.712969
H	11.089081	6.811450	3.115906	C	13.270281	6.208816	0.437342
C	11.186425	5.561721	6.373189	C	12.755114	9.999233	0.831537
H	11.223453	6.529224	6.884109	H	12.361945	10.082839	-0.186670
H	10.572209	4.882280	6.972356	C	13.830845	11.077999	0.997180
H	12.207259	5.165498	6.354631	H	14.686731	10.893588	0.340463
Mg	10.044003	7.462638	-0.643082	H	13.417215	12.061711	0.753505
Mg	6.915630	8.392700	-1.816809	H	14.202191	11.130370	2.025782
H	8.452999	8.499367	-0.283857	C	11.589030	10.243808	1.799104
N	12.022505	7.794458	-0.941623	H	10.753252	9.559451	1.610542
C	12.554297	8.153857	-2.109345	H	11.904346	10.090355	2.837235
C	11.812922	8.359198	-3.283546	H	11.206761	11.265293	1.714961
H	12.362295	8.769723	-4.121867	C	12.764893	5.043058	-0.398052
C	10.434940	8.138505	-3.515323	H	12.012470	5.434424	-1.089893
N	9.647975	7.543952	-2.634877	C	12.089448	3.978443	0.476490
C	14.043536	8.412331	-2.191277	H	11.626279	3.207278	-0.148330
H	14.606834	7.553824	-1.815239	H	12.806710	3.479062	1.135905

H	11.311632	4.418467	1.107469
C	13.885125	4.435414	-1.252573
H	14.321009	5.181036	-1.924885
H	14.688783	4.039195	-0.622048
H	13.499557	3.612321	-1.863158
C	4.374006	9.012736	-3.141343
C	3.319583	8.844263	-2.225776
C	2.301223	9.799588	-2.200935
H	1.474830	9.677880	-1.505552
C	2.335755	10.910484	-3.032821
H	1.537183	11.645154	-2.995446
C	3.411688	11.091738	-3.893876
H	3.454105	11.981399	-4.516084
C	4.444270	10.156793	-3.961380
C	3.297138	7.686696	-1.239519
H	4.191284	7.077805	-1.406865
C	2.079418	6.776255	-1.441399
H	2.048143	6.370918	-2.457177
H	2.114737	5.933965	-0.743241
H	1.143035	7.318241	-1.267783
C	3.352633	8.207175	0.205328
H	4.200149	8.887092	0.338730
H	2.439231	8.753110	0.467895
H	3.465631	7.375317	0.908601
C	5.657946	10.412313	-4.842686
H	6.234620	9.484328	-4.892681
C	6.555624	11.482151	-4.202313
H	7.479413	11.615548	-4.776898
H	6.042859	12.448513	-4.159883
H	6.826093	11.228853	-3.171220
C	5.283694	10.796364	-6.278292
H	6.184874	10.885176	-6.893588
H	4.632896	10.043358	-6.733505
H	4.763096	11.759048	-6.319428
C	6.705074	6.415105	0.183616
H	6.394927	9.870888	-0.863374
H	9.895115	6.443440	0.844166
O	6.345682	5.718597	-0.754248
H	6.136755	7.455648	0.200749

TS3.log

SCF (wB97x) = -3205.81604066
E(SCF)+ZPE(0 K)= -3204.249711
H(298 K)= -3204.157686
G(298 K)= -3204.372818
Lowest Frequency = -627.1500cm-1

Mg	7.707910	9.839498	0.632807
Mg	8.551244	6.810752	1.948162
N	7.980588	11.852117	0.805542
C	8.482015	12.448970	1.887631
C	8.851780	11.779267	3.063538
H	9.357402	12.377908	3.810875
C	8.638987	10.430258	3.410441
N	7.969934	9.590659	2.635627
C	8.659430	13.953138	1.892046
H	9.149395	14.308337	0.983035
H	9.233887	14.278419	2.759781
H	7.677637	14.438177	1.928213
C	9.218351	9.939032	4.716270
H	8.441618	9.678664	5.440696
H	9.865462	10.696719	5.159410
H	9.796426	9.027319	4.534842
N	7.785211	8.278757	3.124013
C	6.757596	8.047014	3.918478
C	6.515007	6.768966	4.477813
H	5.681268	6.710079	5.166907
C	7.287794	5.602449	4.338563
N	8.261287	5.457114	3.444994
C	5.798133	9.163175	4.261129
H	6.274897	9.937196	4.869554
H	4.934745	8.779057	4.805222
H	5.457645	9.651263	3.342504
C	6.991179	4.460740	5.285421
H	6.104741	4.661280	5.887909
H	7.841157	4.307499	5.959367
H	6.848858	3.524560	4.738296
C	7.627016	12.678702	-0.303904
C	8.558119	12.906977	-1.335656
C	8.213730	13.797353	-2.354969
H	8.931519	14.006675	-3.143389
C	6.970745	14.413974	-2.382637
H	6.722985	15.105330	-3.182311
C	6.030483	14.112558	-1.404711
H	5.043894	14.563578	-1.456295
C	6.337086	13.245644	-0.356271
C	9.912696	12.215130	-1.361902

H	9.915660	11.469213	-0.562712
C	11.066513	13.187833	-1.089354
H	10.986705	13.641491	-0.096655
H	12.024260	12.659598	-1.142056
H	11.087370	13.995408	-1.829443
C	10.133032	11.469619	-2.682621
H	9.319441	10.763237	-2.870965
H	10.193952	12.157532	-3.533434
H	11.065483	10.895750	-2.650504
C	5.287545	12.889347	0.685640
H	5.806943	12.459379	1.546877
C	4.339445	11.817190	0.127246
H	3.606867	11.507451	0.880487
H	3.793351	12.192918	-0.743600
H	4.876777	10.920968	-0.204135
C	4.497799	14.102823	1.188344
H	5.165395	14.892241	1.547306
H	3.862578	14.529631	0.405094
H	3.842358	13.808807	2.014085
C	9.098538	4.307355	3.463116
C	8.821442	3.219828	2.613233
C	9.727763	2.158655	2.568699
H	9.528830	1.314536	1.913594
C	10.882323	2.167645	3.340029
H	11.578986	1.336471	3.288832
C	11.144595	3.248048	4.175214
H	12.052958	3.253115	4.771191
C	10.264243	4.326951	4.255533
C	7.571310	3.192167	1.747447
H	6.979258	4.078758	1.994927
C	6.703222	1.959590	2.032276
H	6.439201	1.892445	3.092093
H	5.775733	2.004994	1.452518
H	7.220581	1.033734	1.758726
C	7.925895	3.274701	0.256490
H	8.507381	4.173713	0.033460
H	8.519266	2.407942	-0.055663
H	7.018590	3.303196	-0.356034
C	10.580121	5.522146	5.142201
H	9.666848	6.115271	5.239757
C	11.636252	6.413262	4.476809
H	11.828943	7.309496	5.077205
H	12.581392	5.873386	4.362887
H	11.331077	6.734077	3.475164

C	11.016044	5.114378	6.553988
H	11.129353	6.002187	7.184288
H	10.279648	4.454445	7.023334
H	11.979141	4.592899	6.549292
O	8.072256	6.770189	-0.952867
Mg	9.997414	7.580230	-0.957948
Mg	7.228339	8.322918	-1.999600
H	8.556773	9.345218	-1.003119
N	12.045493	7.790892	-1.120032
C	12.710966	8.148707	-2.220185
C	12.111819	8.454520	-3.448450
H	12.772719	8.835972	-4.217248
C	10.750908	8.347153	-3.818000
N	9.849147	7.782027	-3.041198
C	14.220560	8.265853	-2.165028
H	14.661719	7.325464	-1.820415
H	14.529027	9.035520	-1.451826
H	14.633706	8.509869	-3.144115
C	10.344611	8.933507	-5.150818
H	10.041345	8.164120	-5.866200
H	11.165221	9.505069	-5.585878
H	9.481230	9.590408	-5.010948
N	8.511782	7.772831	-3.503946
C	8.120251	6.758229	-4.246062
C	6.765008	6.590244	-4.641587
H	6.593784	5.821822	-5.385801
C	5.632473	7.309034	-4.234869
N	5.606679	8.162863	-3.206097
C	9.106193	5.696697	-4.673286
H	9.950719	6.105696	-5.232025
H	8.619505	4.932080	-5.279467
H	9.522044	5.226302	-3.775890
C	4.370521	7.097838	-5.042986
H	3.493469	6.991815	-4.400939
H	4.456036	6.220424	-5.684899
H	4.192138	7.970329	-5.681538
C	12.798121	7.543312	0.067735
C	13.151348	8.609180	0.918524
C	13.874346	8.328916	2.079073
H	14.152095	9.141617	2.744490
C	14.242418	7.030058	2.402762
H	14.809168	6.832749	3.307640
C	13.855881	5.983256	1.577193
H	14.112961	4.963535	1.850849

C	13.129740	6.217688	0.409029
C	12.727547	10.041574	0.626494
H	12.342521	10.078690	-0.397791
C	13.892093	11.035171	0.721701
H	14.721784	10.749926	0.067490
H	13.559639	12.037294	0.433239
H	14.281576	11.104557	1.742733
C	11.585720	10.457388	1.562833
H	10.732048	9.778291	1.469352
H	11.907937	10.432025	2.610235
H	11.244491	11.473773	1.344230
C	12.672263	5.044688	-0.443550
H	12.276831	5.451351	-1.379058
C	11.535784	4.289799	0.261866
H	11.154488	3.474128	-0.361872
H	11.869428	3.860970	1.212803
H	10.700372	4.960091	0.491516
C	13.818778	4.095344	-0.810113
H	14.634054	4.632530	-1.304511
H	14.231311	3.596883	0.073491
H	13.462203	3.315085	-1.490368
C	4.453459	8.972829	-2.994520
C	3.501973	8.629895	-2.013910
C	2.415043	9.484045	-1.810684
H	1.668278	9.225795	-1.064678
C	2.267676	10.651284	-2.546049
H	1.416806	11.302775	-2.371442
C	3.219132	10.985757	-3.502771
H	3.105585	11.905881	-4.068536
C	4.316058	10.160313	-3.746198
C	3.604214	7.345366	-1.205714
H	4.559685	6.875051	-1.456732
C	2.492779	6.356015	-1.585463
H	2.517973	6.110537	-2.650988
H	2.602606	5.423889	-1.021593
H	1.503985	6.770646	-1.360472
C	3.591467	7.609668	0.305817
H	4.390469	8.299948	0.588712
H	2.634216	8.031923	0.630697
H	3.743984	6.674577	0.855682
C	5.365800	10.557096	-4.774550
H	5.948316	9.667279	-5.025283
C	6.333314	11.581598	-4.171939
H	7.135849	11.830526	-4.874788
H	5.808485	12.505456	-3.913123
H	6.800906	11.214563	-3.251896
C	4.758145	11.085927	-6.078654
H	5.545341	11.239421	-6.823458
H	4.028509	10.383262	-6.493375
H	4.254230	12.047716	-5.935740
C	7.329473	6.731999	0.122934
H	6.566629	8.414919	0.045302
H	6.291252	6.419523	-0.109415
H	9.968831	7.450447	0.941066

TS4.log

SCF (wB97x) = -3319.18539558
E(SCF)+ZPE(0 K)= -3317.606435
H(298 K)= -3317.511777
G(298 K)= -3317.734145
Lowest Frequency = -125.1561cm-1

Mg	8.087176	10.026586	0.585182
Mg	8.655263	6.864225	2.224573
N	8.366797	12.021372	0.804829
C	8.767219	12.623948	1.919178
C	8.990166	11.945919	3.128545
H	9.428683	12.531150	3.927501
C	8.668891	10.610541	3.462502
N	8.105672	9.761244	2.615903
C	9.031065	14.112995	1.897973
H	9.833393	14.342828	1.188442
H	9.315695	14.483137	2.883261
H	8.146181	14.657874	1.557469
C	8.994172	10.153682	4.866274
H	8.090051	9.965233	5.451986
H	9.592154	10.902038	5.387008
H	9.545636	9.209492	4.832109
N	7.744211	8.485272	3.127328
C	6.544063	8.389703	3.675354
C	5.990516	7.170723	4.133146
H	5.022966	7.259743	4.611293
C	6.485693	5.861071	4.012824
N	7.606023	5.541060	3.374238
C	5.671309	9.620783	3.792180
H	6.117296	10.382549	4.437524
H	4.688353	9.361901	4.185965

H	5.546634	10.081871	2.806992
C	5.660092	4.768972	4.660154
H	4.647517	5.114337	4.872007
H	6.117922	4.462362	5.606278
H	5.611189	3.880096	4.026794
C	7.939556	12.795012	-0.314616
C	8.841093	13.120263	-1.343046
C	8.365132	13.822137	-2.453036
H	9.049290	14.077842	-3.257348
C	7.031580	14.196299	-2.548304
H	6.680274	14.747803	-3.414968
C	6.144418	13.838561	-1.540664
H	5.095181	14.106233	-1.631801
C	6.572036	13.122059	-0.423547
C	10.292163	12.670331	-1.293249
H	10.500617	12.328606	-0.274293
C	11.277544	13.800720	-1.610886
H	11.114521	14.665156	-0.959696
H	12.306453	13.454829	-1.470277
H	11.185582	14.140819	-2.647576
C	10.513200	11.478791	-2.234885
H	9.923167	10.605557	-1.928835
H	10.225205	11.730029	-3.261776
H	11.562326	11.171094	-2.252301
C	5.563540	12.689769	0.630758
H	6.089223	12.044862	1.341776
C	4.420848	11.868321	0.018978
H	3.770105	11.474452	0.807796
H	3.797216	12.474028	-0.647206
H	4.805194	11.024333	-0.560263
C	5.024076	13.889037	1.421215
H	5.831853	14.438757	1.914151
H	4.494135	14.587152	0.763672
H	4.322061	13.555177	2.192518
C	8.049655	4.188632	3.321158
C	7.866352	3.448322	2.133044
C	8.379392	2.150844	2.069031
H	8.231402	1.563655	1.167271
C	9.074587	1.596551	3.134810
H	9.471845	0.588826	3.063002
C	9.260072	2.340213	4.293137
H	9.812861	1.906028	5.121519
C	8.752893	3.634319	4.412056
C	7.098671	4.009054	0.941755
H	6.976959	5.085461	1.093758
C	5.686770	3.413047	0.860152
H	5.108619	3.636409	1.762333
H	5.148063	3.826675	0.000678
H	5.725959	2.324227	0.744835
C	7.849181	3.811638	-0.382594
H	8.870840	4.200646	-0.316490
H	7.909062	2.753954	-0.660680
H	7.336236	4.336668	-1.196743
C	8.991975	4.419593	5.694671
H	8.364572	5.314127	5.666431
C	10.448541	4.894325	5.785929
H	10.611502	5.462980	6.707675
H	11.138938	4.043854	5.785358
H	10.709248	5.534614	4.939668
C	8.604896	3.621660	6.947094
H	8.665871	4.258908	7.834917
H	7.585377	3.230149	6.875978
H	9.275806	2.771325	7.108195
O	8.902752	8.442867	-0.321591
Mg	10.641892	7.619763	-0.915225
Mg	7.515149	8.569926	-1.868884
H	6.889841	9.990197	-0.867029
N	12.527741	8.246863	-1.443115
C	12.896854	8.722047	-2.629781
C	12.050585	8.802015	-3.745691
H	12.462755	9.285622	-4.622649
C	10.710506	8.369237	-3.860837
N	10.089506	7.706136	-2.903391
C	14.308488	9.239258	-2.807104
H	15.033596	8.428033	-2.689968
H	14.542622	9.982765	-2.038539
H	14.447508	9.688376	-3.791000
C	9.966816	8.719053	-5.129005
H	9.569663	7.827617	-5.622145
H	10.613493	9.251383	-5.827155
H	9.104988	9.352404	-4.891899
N	8.704631	7.456410	-3.089432
C	8.354530	6.270161	-3.553732
C	7.004923	5.878966	-3.704735
H	6.849108	4.916934	-4.177184
C	5.843251	6.603739	-3.382876
N	5.843218	7.739364	-2.692890
C	9.424270	5.277442	-3.940865

H	10.073133	5.675203	-4.726248
H	8.983340	4.341695	-4.285393
H	10.068184	5.070479	-3.079538
C	4.528548	6.037143	-3.872253
H	3.841570	5.865872	-3.039350
H	4.673617	5.099610	-4.409518
H	4.039456	6.753309	-4.540556
C	13.501956	8.103938	-0.411543
C	13.571929	9.059179	0.618588
C	14.476226	8.861424	1.664053
H	14.534691	9.593502	2.465330
C	15.294391	7.741143	1.700801
H	15.991733	7.599313	2.520611
C	15.205055	6.794444	0.687059
H	15.830821	5.907476	0.730993
C	14.315346	6.952192	-0.375164
C	12.648904	10.264780	0.636119
H	12.097853	10.270830	-0.308072
C	13.409813	11.591961	0.727500
H	14.111986	11.703373	-0.104835
H	12.710246	12.434266	0.699761
H	13.979982	11.667843	1.659356
C	11.624151	10.144792	1.768939
H	11.040998	9.220195	1.685353
H	12.114843	10.131590	2.748718
H	10.927475	10.987432	1.760867
C	14.189205	5.860382	-1.427169
H	13.592345	6.256370	-2.253119
C	13.427278	4.658352	-0.848773
H	13.267574	3.894232	-1.617711
H	13.990518	4.201334	-0.027500
H	12.454651	4.969831	-0.453024
C	15.542840	5.429497	-2.004220
H	16.101990	6.284428	-2.397618
H	16.168724	4.939383	-1.250984
H	15.394776	4.715508	-2.820706
C	4.625260	8.458745	-2.509958
C	3.780546	8.184447	-1.417873
C	2.625156	8.951881	-1.257167
H	1.965431	8.750871	-0.417294
C	2.312473	9.978677	-2.137506
H	1.410892	10.566091	-1.993438
C	3.173345	10.264702	-3.189350
H	2.943485	11.088130	-3.860072
C	4.335519	9.520414	-3.390663
C	4.129997	7.131603	-0.377351
H	5.024267	6.600928	-0.721111
C	3.017515	6.094339	-0.180228
H	2.770987	5.580514	-1.114367
H	3.328794	5.340225	0.549549
H	2.099623	6.558524	0.195703
C	4.471863	7.804922	0.958845
H	5.241219	8.572726	0.825253
H	3.590917	8.297313	1.386673
H	4.840655	7.077840	1.689289
C	5.301686	9.899969	-4.502352
H	6.047607	9.102983	-4.576641
C	6.039791	11.197139	-4.136994
H	6.809272	11.434861	-4.880117
H	5.345360	12.042867	-4.089714
H	6.515185	11.131383	-3.151642
C	4.618941	10.016393	-5.869298
H	5.361527	10.215680	-6.648738
H	4.092289	9.092667	-6.127966
H	3.891921	10.835296	-5.891428
C	10.889667	6.326821	2.271459
C	8.128440	7.261868	0.192510
H	7.051849	7.536772	0.187988
H	8.212637	6.457542	-0.555278
O	11.933352	5.984649	2.590498
H	10.874321	6.444848	0.475025

TS5.log

SCF (wB97x) = -3319.18214038
E(SCF)+ZPE(0 K)= -3317.600392
H(298 K)= -3317.506276
G(298 K)= -3317.726309
Lowest Frequency = -136.5703cm-1

Mg	7.848352	9.924280	0.634766
Mg	8.598293	6.777367	2.280320
N	8.317200	11.875991	0.834297
C	8.904043	12.374398	1.921109
C	9.162149	11.625092	3.078181
H	9.757628	12.116536	3.837428
C	8.695239	10.332592	3.421576
N	7.915019	9.609096	2.635267

C	9.319859	13.827958	1.927292
H	10.003429	14.036294	1.098314
H	9.807446	14.100176	2.863638
H	8.445823	14.471491	1.784854
C	9.107187	9.774789	4.761546
H	8.236881	9.568652	5.391273
H	9.769279	10.464264	5.285473
H	9.628800	8.823937	4.613289
N	7.530069	8.328991	3.109296
C	6.345029	8.224137	3.673819
C	5.810919	6.983866	4.102272
H	4.854057	7.042512	4.606237
C	6.339697	5.692784	3.945315
N	7.463099	5.410238	3.289176
C	5.497795	9.460510	3.867560
H	6.023378	10.215524	4.459279
H	4.555156	9.217152	4.358740
H	5.281061	9.919291	2.896418
C	5.561975	4.562300	4.587540
H	4.574903	4.893475	4.911866
H	6.102480	4.186783	5.463411
H	5.449644	3.718338	3.902233
C	7.872802	12.736863	-0.211401
C	8.715602	13.047358	-1.295125
C	8.215364	13.833630	-2.334247
H	8.856837	14.075340	-3.177439
C	6.908237	14.301969	-2.314744
H	6.534897	14.913697	-3.130225
C	6.075792	13.964042	-1.255624
H	5.045372	14.309730	-1.254130
C	6.532678	13.175460	-0.199175
C	10.120186	12.476450	-1.383063
H	10.356610	12.032475	-0.412852
C	11.188141	13.532829	-1.681479
H	11.178192	14.333805	-0.935705
H	12.181968	13.073827	-1.675710
H	11.043679	13.990418	-2.665724
C	10.164013	11.353017	-2.424766
H	9.421928	10.582535	-2.190289
H	9.930524	11.730783	-3.426472
H	11.147544	10.874115	-2.466722
C	5.574302	12.782783	0.915713
H	6.130463	12.171527	1.632728
C	4.416257	11.929654	0.375670
H	3.775607	11.589446	1.197234
H	3.789921	12.500969	-0.318233
H	4.785495	11.051488	-0.163082
C	5.055539	14.009889	1.675985
H	5.881474	14.598391	2.087684
H	4.468733	14.665571	1.023301
H	4.410846	13.701435	2.505539
C	7.918853	4.064425	3.198643
C	7.686502	3.329282	2.017570
C	8.183206	2.026166	1.934036
H	7.994470	1.440645	1.038553
C	8.916906	1.466967	2.971051
H	9.296499	0.453287	2.886777
C	9.175090	2.216775	4.112663
H	9.770563	1.782845	4.910713
C	8.687808	3.516443	4.247442
C	6.908574	3.917647	0.847839
H	6.825605	4.995319	1.015298
C	5.481392	3.359595	0.781292
H	4.906969	3.623317	1.674814
H	4.956625	3.763568	-0.090905
H	5.490775	2.267445	0.694242
C	7.628736	3.709220	-0.491907
H	8.674288	4.028069	-0.431327
H	7.616962	2.657698	-0.799187
H	7.143451	4.290352	-1.284357
C	9.036549	4.344367	5.475802
H	8.345850	5.190722	5.517289
C	10.453644	4.923018	5.340828
H	10.699491	5.551591	6.203310
H	11.195837	4.119065	5.282268
H	10.552698	5.534504	4.437444
C	8.886723	3.565556	6.787523
H	9.028722	4.235877	7.640942
H	7.894011	3.111964	6.870809
H	9.629188	2.765397	6.875615
O	8.783207	8.408688	-0.274509
Mg	10.622717	8.029633	-0.828840
Mg	7.436913	8.477148	-1.827347
H	6.679081	9.835152	-0.815598
N	12.548850	8.461771	-1.294019
C	12.975206	8.704591	-2.533941
C	12.160213	8.679444	-3.676577
H	12.631359	9.012114	-4.593484

C	10.806648	8.292530	-3.802236
N	10.114433	7.775248	-2.802198
C	14.418206	9.104160	-2.757701
H	15.099400	8.517921	-2.138076
H	14.554788	10.153782	-2.477310
H	14.699722	8.991067	-3.805107
C	10.137350	8.521917	-5.136997
H	9.787813	7.589601	-5.588067
H	10.816323	9.014415	-5.833315
H	9.251392	9.149403	-4.995285
N	8.744717	7.486981	-3.043107
C	8.440201	6.285467	-3.509692
C	7.110169	5.865972	-3.746906
H	7.008183	4.900757	-4.227857
C	5.906594	6.546233	-3.475054
N	5.842679	7.654748	-2.751997
C	9.542501	5.289794	-3.787189
H	10.234577	5.643350	-4.555944
H	9.130381	4.330327	-4.100175
H	10.135549	5.141638	-2.878449
C	4.632080	5.956561	-4.033884
H	3.946160	5.685175	-3.226246
H	4.830523	5.072203	-4.639914
H	4.116322	6.699687	-4.650227
C	13.493989	8.383143	-0.221925
C	13.884139	9.533729	0.487906
C	14.754934	9.385253	1.568065
H	15.060496	10.263554	2.129921
C	15.228300	8.137264	1.947950
H	15.904986	8.043549	2.791736
C	14.827403	7.008319	1.246007
H	15.200413	6.033695	1.546558
C	13.954985	7.106342	0.161487
C	13.373321	10.924071	0.137667
H	12.777886	10.842876	-0.778007
C	14.525608	11.901831	-0.135018
H	15.213683	11.519737	-0.894924
H	14.137012	12.866642	-0.477063
H	15.108392	12.089172	0.772861
C	12.460403	11.476121	1.242562
H	11.599336	10.828008	1.421134
H	13.003961	11.558123	2.189971
H	12.101538	12.477185	0.978094
C	13.554907	5.852328	-0.604829

H	12.668693	6.096302	-1.199388
C	13.186014	4.680179	0.312317
H	12.775196	3.855794	-0.279798
H	14.057528	4.290613	0.848625
H	12.437534	4.976230	1.053649
C	14.653592	5.441568	-1.596092
H	14.850274	6.234307	-2.323452
H	15.589304	5.226327	-1.068360
H	14.359400	4.542443	-2.147785
C	4.620836	8.362898	-2.559392
C	3.754291	8.032020	-1.499295
C	2.630675	8.832135	-1.282191
H	1.957163	8.592512	-0.463850
C	2.366504	9.939401	-2.077174
H	1.488909	10.550032	-1.888692
C	3.244459	10.272236	-3.100129
H	3.052879	11.155153	-3.704133
C	4.381167	9.504027	-3.351644
C	4.043291	6.880644	-0.546685
H	4.912921	6.333866	-0.927753
C	2.869818	5.896433	-0.452863
H	2.611959	5.478609	-1.430942
H	3.118414	5.068753	0.217893
H	1.973483	6.382867	-0.054352
C	4.405688	7.407952	0.849819
H	5.212105	8.146937	0.799981
H	3.544864	7.895561	1.321365
H	4.734246	6.596990	1.507850
C	5.368286	9.945314	-4.421763
H	6.147394	9.179638	-4.486886
C	6.043991	11.267210	-4.023341
H	6.827427	11.535898	-4.740775
H	5.320401	12.088952	-3.996569
H	6.492118	11.213683	-3.024740
C	4.715860	10.049491	-5.805196
H	5.462705	10.312546	-6.561463
H	4.254906	9.100476	-6.096127
H	3.938053	10.820483	-5.825469
C	10.673047	7.382314	1.822598
C	8.180928	7.097494	0.165209
H	7.077083	7.211358	0.120656
H	8.417570	6.356686	-0.614505
O	11.221496	8.455156	1.945347
H	11.019055	6.803624	0.793425

TS6.log

SCF (wB97x) = -3319.20042056
E(SCF)+ZPE(0 K)= -3317.615445
H(298 K)= -3317.521825
G(298 K)= -3317.740610
Lowest Frequency = -276.6315cm-1

Mg	8.202602	9.913570	0.682994
Mg	8.776368	6.844391	2.685395
N	8.439154	11.923596	0.865521
C	8.853608	12.541252	1.966518
C	9.075172	11.881212	3.187559
H	9.518154	12.474720	3.977789
C	8.690736	10.572283	3.552176
N	8.135341	9.718027	2.704617
C	9.108415	14.031424	1.926379
H	9.870945	14.265581	1.176077
H	9.439133	14.406516	2.895219
H	8.202263	14.568303	1.631136
C	8.918063	10.155288	4.986379
H	7.971985	10.010098	5.515802
H	9.501145	10.906278	5.519837
H	9.446636	9.197960	5.021998
N	7.688271	8.477723	3.233021
C	6.449525	8.441519	3.703370
C	5.836679	7.264319	4.181023
H	4.856196	7.400439	4.619982
C	6.293760	5.934811	4.114323
N	7.421701	5.566142	3.521851
C	5.613990	9.702000	3.705859
H	6.084257	10.513488	4.267310
H	4.625855	9.509907	4.124214
H	5.493073	10.061119	2.678393
C	5.427285	4.889501	4.784336
H	4.429689	5.276000	4.995928
H	5.886945	4.591566	5.733416
H	5.342657	3.985559	4.177773
C	7.951931	12.690206	-0.235889
C	8.807396	13.054867	-1.288805
C	8.276614	13.755586	-2.373583
H	8.926969	14.040513	-3.195878
C	6.929173	14.086942	-2.422351
H	6.532853	14.636301	-3.270805
C	6.086782	13.690667	-1.391100
H	5.027603	13.927570	-1.444363
C	6.573502	12.980082	-0.294068
C	10.265644	12.634450	-1.289335
H	10.511512	12.291327	-0.279669
C	11.226253	13.777262	-1.633131
H	11.067064	14.641019	-0.980111
H	12.262437	13.445115	-1.513733
H	11.105485	14.113101	-2.668363
C	10.466652	11.449922	-2.241274
H	9.882325	10.576148	-1.924508
H	10.148838	11.702917	-3.258730
H	11.515043	11.146011	-2.286719
C	5.618062	12.520257	0.796410
H	6.191605	11.901197	1.492476
C	4.488167	11.653114	0.227510
H	3.871117	11.248709	1.038385
H	3.827640	12.227246	-0.430639
H	4.885911	10.815536	-0.351786
C	5.062533	13.704364	1.597969
H	5.866980	14.289628	2.054203
H	4.479327	14.375467	0.957663
H	4.405594	13.349392	2.399139
C	7.770578	4.184234	3.449522
C	7.473467	3.457531	2.278027
C	7.858538	2.117379	2.211824
H	7.624147	1.538940	1.323136
C	8.544627	1.511513	3.256156
H	8.840214	0.469501	3.182762
C	8.863449	2.249674	4.388386
H	9.420921	1.778690	5.193164
C	8.485625	3.587522	4.505996
C	6.734829	4.095239	1.109139
H	6.816420	5.179279	1.224147
C	5.239850	3.749668	1.133783
H	4.745507	4.157661	2.020589
H	4.742367	4.162384	0.250048
H	5.090578	2.664281	1.129710
C	7.339735	3.723272	-0.250738
H	8.422028	3.885578	-0.260717
H	7.152619	2.674354	-0.505564
H	6.898235	4.336227	-1.044545
C	8.899252	4.390477	5.729549

H	8.340666	5.330853	5.716381
C	10.393044	4.740822	5.651138
H	10.687716	5.384080	6.487333
H	11.005875	3.833574	5.688246
H	10.631418	5.255927	4.714226
C	8.571079	3.680461	7.048092
H	8.792781	4.335754	7.896395
H	7.513987	3.401455	7.098616
H	9.163277	2.768329	7.176047
O	9.012637	8.260444	-0.148172
Mg	10.836779	7.728431	-0.893397
Mg	7.574700	8.345678	-1.657733
H	6.999188	9.824689	-0.735565
N	12.649741	8.423512	-1.538580
C	12.906773	8.893999	-2.757176
C	11.991067	8.882855	-3.820708
H	12.313672	9.376258	-4.729045
C	10.679150	8.362012	-3.846428
N	10.152584	7.698609	-2.832069
C	14.257249	9.520670	-3.035287
H	15.051921	8.769432	-2.996657
H	14.493975	10.268529	-2.271905
H	14.278646	9.993826	-4.017530
C	9.844835	8.625797	-5.078656
H	9.497078	7.697865	-5.540641
H	10.408713	9.196398	-5.817078
H	8.947082	9.189678	-4.802543
N	8.783335	7.348788	-2.940840
C	8.474924	6.158745	-3.424605
C	7.135645	5.722790	-3.564121
H	7.005296	4.752340	-4.027258
C	5.959081	6.434874	-3.269083
N	5.941711	7.565519	-2.571109
C	9.576205	5.220355	-3.855282
H	10.142919	5.628887	-4.697021
H	9.171351	4.250065	-4.144042
H	10.288801	5.082711	-3.035783
C	4.656450	5.874155	-3.793740
H	3.946565	5.710215	-2.977236
H	4.810652	4.933543	-4.323270
H	4.190576	6.590022	-4.478686
C	13.710271	8.352905	-0.586116
C	13.754880	9.273494	0.475716
C	14.763398	9.150378	1.433098
H	14.804636	9.853652	2.260298
C	15.707252	8.136733	1.350902
H	16.487195	8.053743	2.101608
C	15.639666	7.218022	0.309693
H	16.366742	6.412676	0.264907
C	14.647256	7.301369	-0.665950
C	12.680126	10.334832	0.634268
H	12.126405	10.381809	-0.308156
C	13.242734	11.735619	0.894741
H	13.931048	12.039625	0.099832
H	12.429960	12.468474	0.942990
H	13.782994	11.786697	1.845706
C	11.695302	9.924022	1.739455
H	11.294909	8.917447	1.570135
H	12.196704	9.907898	2.714070
H	10.870019	10.639943	1.809262
C	14.536001	6.230541	-1.742003
H	13.937298	6.633961	-2.562743
C	13.778386	5.013417	-1.189078
H	13.624664	4.265566	-1.975296
H	14.348626	4.543780	-0.379542
H	12.806252	5.306213	-0.779589
C	15.889806	5.818539	-2.330143
H	16.454855	6.686850	-2.684655
H	16.510438	5.290194	-1.598777
H	15.739689	5.140912	-3.176525
C	4.764225	8.364752	-2.502189
C	3.816660	8.174692	-1.479196
C	2.725271	9.042389	-1.411467
H	1.989003	8.913911	-0.623227
C	2.573296	10.082256	-2.319297
H	1.719160	10.748545	-2.246889
C	3.531463	10.278346	-3.304932
H	3.424146	11.107963	-3.998741
C	4.637116	9.434471	-3.411811
C	4.003184	7.103020	-0.416467
H	4.791948	6.423978	-0.758136
C	2.735652	6.272320	-0.185024
H	2.389953	5.802609	-1.111349
H	2.925902	5.483821	0.548877
H	1.916370	6.885809	0.203516
C	4.484043	7.735859	0.896654
H	5.377460	8.349270	0.733591
H	3.713202	8.391276	1.318827

H	4.726467	6.971871	1.642530
C	5.699046	9.705103	-4.468608
H	6.421106	8.883929	-4.430422
C	6.456298	11.006066	-4.161672
H	7.267411	11.162839	-4.882038
H	5.790351	11.873769	-4.215090
H	6.885491	11.004653	-3.153560
C	5.112925	9.727746	-5.885205
H	5.909811	9.855346	-6.625234
H	4.586364	8.794799	-6.108675
H	4.404379	10.552817	-6.015762
O	11.114906	6.517837	0.571561
C	10.196381	6.084729	1.369054
H	9.841762	5.062897	1.073635
C	8.412426	7.032043	0.384600
H	7.360210	7.244989	0.671700
H	8.377577	6.271892	-0.408649

TS7.log

SCF (wB97x) = -3319.23194602
E(SCF)+ZPE(0 K)= -3317.645586
H(298 K)= -3317.552473
G(298 K)= -3317.770815
Lowest Frequency = -462.9779cm-1

Mg	7.661875	10.042294	0.404192
Mg	8.979649	6.739599	2.353151
N	8.024186	12.025372	0.729179
C	8.482411	12.547736	1.858039
C	8.763758	11.794041	3.009996
H	9.237700	12.339020	3.816762
C	8.548870	10.423940	3.261158
N	7.946693	9.592442	2.416952
C	8.776450	14.030378	1.927155
H	9.632955	14.273293	1.288859
H	9.003918	14.344432	2.946167
H	7.930616	14.613378	1.553827
C	9.060992	9.901538	4.587509
H	8.254158	9.543638	5.232759
H	9.611840	10.677695	5.118867
H	9.729586	9.051039	4.418846
N	7.743176	8.271942	2.919777
C	6.652170	8.090263	3.652576
C	6.341598	6.881096	4.315615
H	5.464734	6.919503	4.950329
C	7.008107	5.644502	4.280717
N	8.049708	5.391610	3.498193
C	5.671526	9.229477	3.824319
H	6.101010	10.051572	4.404498
H	4.766312	8.886246	4.325457
H	5.404217	9.644609	2.848195
C	6.518740	4.573552	5.230960
H	5.506045	4.777539	5.581418
H	7.185041	4.552138	6.101421
H	6.552223	3.579880	4.782402
C	7.725562	12.873628	-0.379922
C	8.735723	13.213169	-1.300062
C	8.396361	13.976944	-2.418394
H	9.166587	14.241033	-3.137974
C	7.090906	14.402561	-2.627512
H	6.843890	15.000531	-3.499346
C	6.100686	14.045630	-1.721346
H	5.076817	14.364607	-1.896440
C	6.391788	13.269556	-0.599176
C	10.159398	12.710489	-1.125995
H	10.248618	12.321008	-0.110777
C	11.217225	13.806851	-1.289249
H	11.013355	14.661518	-0.636617
H	12.206651	13.410870	-1.036088
H	11.266895	14.177053	-2.318713
C	10.434476	11.542153	-2.078546
H	9.752393	10.709048	-1.879686
H	10.300176	11.841797	-3.124203
H	11.454106	11.160431	-1.969631
C	5.273369	12.866043	0.349517
H	5.695961	12.163396	1.075010
C	4.131549	12.147638	-0.382200
H	3.380904	11.798171	0.335220
H	3.622876	12.812450	-1.088944
H	4.503283	11.284429	-0.940580
C	4.753760	14.074604	1.140264
H	5.556022	14.552110	1.711502
H	4.325574	14.827427	0.469081
H	3.972452	13.767813	1.843581
C	8.522299	4.061126	3.302771
C	7.890209	3.249517	2.335236
C	8.396598	1.968505	2.117745

H	7.914497	1.321547	1.390476
C	9.520102	1.510982	2.797171
H	9.902046	0.512638	2.607232
C	10.164155	2.343295	3.702210
H	11.059905	1.993113	4.207855
C	9.681054	3.625880	3.969283
C	6.697748	3.750701	1.528987
H	6.666075	4.838072	1.644836
C	5.372471	3.177582	2.050956
H	5.179980	3.468669	3.086464
H	4.534446	3.536381	1.444055
H	5.377148	2.082893	2.002035
C	6.847722	3.467067	0.029300
H	7.809892	3.833512	-0.334914
H	6.766489	2.398172	-0.196665
H	6.054355	3.979316	-0.527129
C	10.433400	4.544193	4.919272
H	9.807802	5.427149	5.087473
C	11.750982	5.016142	4.282812
H	12.266851	5.729443	4.934525
H	12.424198	4.168815	4.110846
H	11.596086	5.498326	3.310877
C	10.681220	3.897770	6.287114
H	11.151569	4.614564	6.967878
H	9.743498	3.559867	6.738717
H	11.347116	3.031891	6.209297
O	8.477043	8.591270	-0.607696
Mg	10.362577	8.011057	-0.755301
Mg	7.461951	8.916853	-2.248909
H	6.611027	10.252407	-1.210674
N	12.368823	8.368176	-0.922999
C	13.019859	8.729088	-2.025512
C	12.411211	8.897730	-3.278773
H	13.043386	9.294878	-4.063864
C	11.076018	8.614080	-3.657390
N	10.210644	8.052377	-2.838003
C	14.502493	9.027849	-1.941770
H	15.036696	8.217716	-1.438564
H	14.672626	9.932011	-1.347000
H	14.935485	9.177026	-2.931478
C	10.651498	8.995066	-5.057175
H	10.338061	8.122931	-5.637531
H	11.460498	9.497160	-5.588803
H	9.786599	9.664708	-5.009957
N	8.886696	7.848009	-3.317299
C	8.621927	6.665787	-3.836335
C	7.312358	6.256427	-4.205448
H	7.262378	5.332868	-4.769336
C	6.080250	6.861284	-3.921071
N	5.931327	7.928774	-3.136710
C	9.739278	5.667263	-4.036660
H	10.527493	6.058917	-4.685151
H	9.360819	4.737073	-4.461314
H	10.208969	5.452411	-3.070966
C	4.850141	6.234235	-4.539546
H	4.140557	5.923617	-3.767908
H	5.106072	5.369776	-5.152637
H	4.331450	6.968542	-5.164640
C	13.086021	8.097987	0.279652
C	13.363235	9.131014	1.192979
C	13.988944	8.812533	2.400982
H	14.203552	9.602017	3.116177
C	14.338818	7.504051	2.704918
H	14.833039	7.273539	3.644038
C	14.042468	6.488922	1.803023
H	14.300829	5.463523	2.052012
C	13.401378	6.759367	0.594916
C	12.933361	10.563912	0.922393
H	12.668991	10.641283	-0.136098
C	14.044410	11.584687	1.193786
H	14.961385	11.329458	0.653639
H	13.725502	12.582802	0.875399
H	14.290442	11.646182	2.259131
C	11.675900	10.897818	1.734541
H	10.820176	10.284486	1.427520
H	11.838356	10.721644	2.803541
H	11.393445	11.948101	1.614346
C	13.055773	5.619682	-0.351446
H	12.351471	6.011722	-1.091611
C	12.368261	4.449763	0.362997
H	12.039581	3.704233	-0.369235
H	13.042817	3.942571	1.061677
H	11.493432	4.801444	0.915469
C	14.297336	5.145341	-1.119869
H	14.739263	5.957898	-1.704499
H	15.062236	4.770477	-0.430284
H	14.037746	4.335379	-1.809922
C	4.635123	8.469576	-2.889150

C	3.862338	8.006527	-1.804861
C	2.634656	8.618008	-1.544092
H	2.031677	8.267162	-0.710493
C	2.174598	9.672870	-2.320748
H	1.218893	10.138445	-2.100591
C	2.955017	10.138243	-3.370739
H	2.606481	10.979863	-3.963372
C	4.186994	9.555309	-3.668517
C	4.348374	6.893819	-0.887739
H	5.302060	6.532579	-1.281432
C	3.383857	5.702034	-0.847728
H	3.205979	5.293389	-1.846573
H	3.795577	4.901822	-0.224141
H	2.414134	5.985811	-0.425291
C	4.597008	7.426147	0.531771
H	5.248551	8.305891	0.507659
H	3.658923	7.731091	1.008469
H	5.064597	6.664558	1.166033
C	5.047022	10.137386	-4.779545
H	5.904706	9.472070	-4.915710
C	5.583983	11.517878	-4.370826
H	6.273052	11.909398	-5.127306
H	4.765844	12.238069	-4.257419
H	6.106790	11.479533	-3.409312
C	4.306339	10.205070	-6.120252
H	4.977578	10.561644	-6.908284
H	3.928014	9.221036	-6.414186
H	3.454451	10.892173	-6.076918
O	10.213617	6.961990	0.927160
C	9.202483	6.214960	0.183147
H	9.612823	5.525431	-0.557222
C	8.045215	6.984676	-0.133191
H	7.389242	7.329026	0.671074
H	7.445693	6.534045	-0.930503

8) References

[S1] Intemann J.; Spielmann J.; Sirsch P.; Harder S. *Chem. Eur. J.*, **2013**, *19*, 8478 – 8489.

[S2] O.V. Dolomanov, L.J. Bourhis, R.J. Gildea, J.A.K. Howard, H. Puschmann, J. Appl. Cryst., 2009, 42, 339-341.

[S3] SHELXTL v5.1, Bruker AXS, Madison, WI, 1998.

[S4] SHELX-2013, G.M. Sheldrick, Acta Cryst., 2015, C71, 3-8.

[S5] A.L. Spek (2003, 2009) PLATON, A Multipurpose Crystallographic Tool, Utrecht University, Utrecht, The Netherlands. See also A.L. Spek, *Acta. Cryst.*, 2015, **C71**, 9-18.

[S6] D. J. Frisch, M. J.; Trucks, G. W.; Schlegel, H. B.; Scuseria, G. E.; Robb, M. A.; Cheeseman, J. R.; Scalmani, G.; Barone, V.; Mennucci, B.; Petersson, G. A.; Nakatsuji, H.; Caricato, M.; Li, X.; Hratchian, H. P.; Izmaylov, A. F.; Bloino, J.; Zheng, G.; Sonnenb, 2009.

[S7] E. D. Glendening, C. R. Landis, F. Weinhold, J. Comput. Chem. 2013, 34, 1429–1437.

[S8] T. A. Keith, AIMALL (Version 19.10.12). TK Gristmill Software, Overland Park KS, USA., 2019.

[S9] F. Cortés-Guzmán and R. F. W. Bader, Coord. Chem. Rev., 2005, 249, 633–662.

.
